# Implications of virus-induced stress granules in tauopathies

**DOI:** 10.1186/s40035-026-00538-4

**Published:** 2026-02-12

**Authors:** Snigdda Sharma, Alex Vandenakker, Claudia Cortés-Pérez, Sarah Milne, Renée N. Douville

**Affiliations:** 1 Division of Neurodegenerative & Neurodevelopmental Disorders, St. Boniface Hospital Albrechtsen Research Centre, 351 Taché Ave, Winnipeg, MB R2H 2A6 Canada; 2 Department of Microbiology, University of Manitoba, Winnipeg, MB R3T 2N2 Canada; 3 Neuroscience Program, University of Winnipeg, 599 Portage Avenue, Winnipeg, MB R3B 2G3 Canada; 4 Department of Pharmacology and Therapeutics, University of Manitoba, 753 McDermot Ave, Winnipeg, MB R3E 0T6 Canada

**Keywords:** Tauopathy, Microtubule-associated protein tau, Virus, Liquid–liquid phase separation, Stress granule, G3BP1, Neurodegenerative disease, Dementia, Endogenous retroviruses, Transposable elements

## Abstract

Tauopathies are characterized by aberrant tau structure and function, which is associated with neurodegenerative dementias, such as Alzheimer’s disease, Pick’s disease, and frontotemporal dementia, as well as the motor neuron disease amyotrophic lateral sclerosis. Consistent association of these neurodegenerative conditions with viruses suggests an interplay between viral activity and the development of tauopathy. In this review, we explore how tau dysregulation may facilitate viral activity, and conversely, how viruses may drive tauopathy. We further discuss how stress granules (SGs) are a likely hub for the interactions between tau and viral components, leading to tau deregulation. Within the network of SG proteins analyzed, 15 proteins were identified to be both tau interactors and implicated in viral processes, having dual functionality. These SG proteins are further discussed in terms of their relationship with tauopathy, viral replication, and neurodegeneration. Concrete examples of synergistic and competing effects between tau and viruses are highlighted, revealing both pathological and protective mechanisms. This dichotomy underscores a complexity that is both disease- and virus-specific, within the context of SG biology and tau pathology. While the viral involvement in tauopathies could be considered detrimental, it may provide insights into antiviral therapeutics to target the accumulation and misfolding of tau in these neurodegenerative diseases.

**Graphical abstract:**

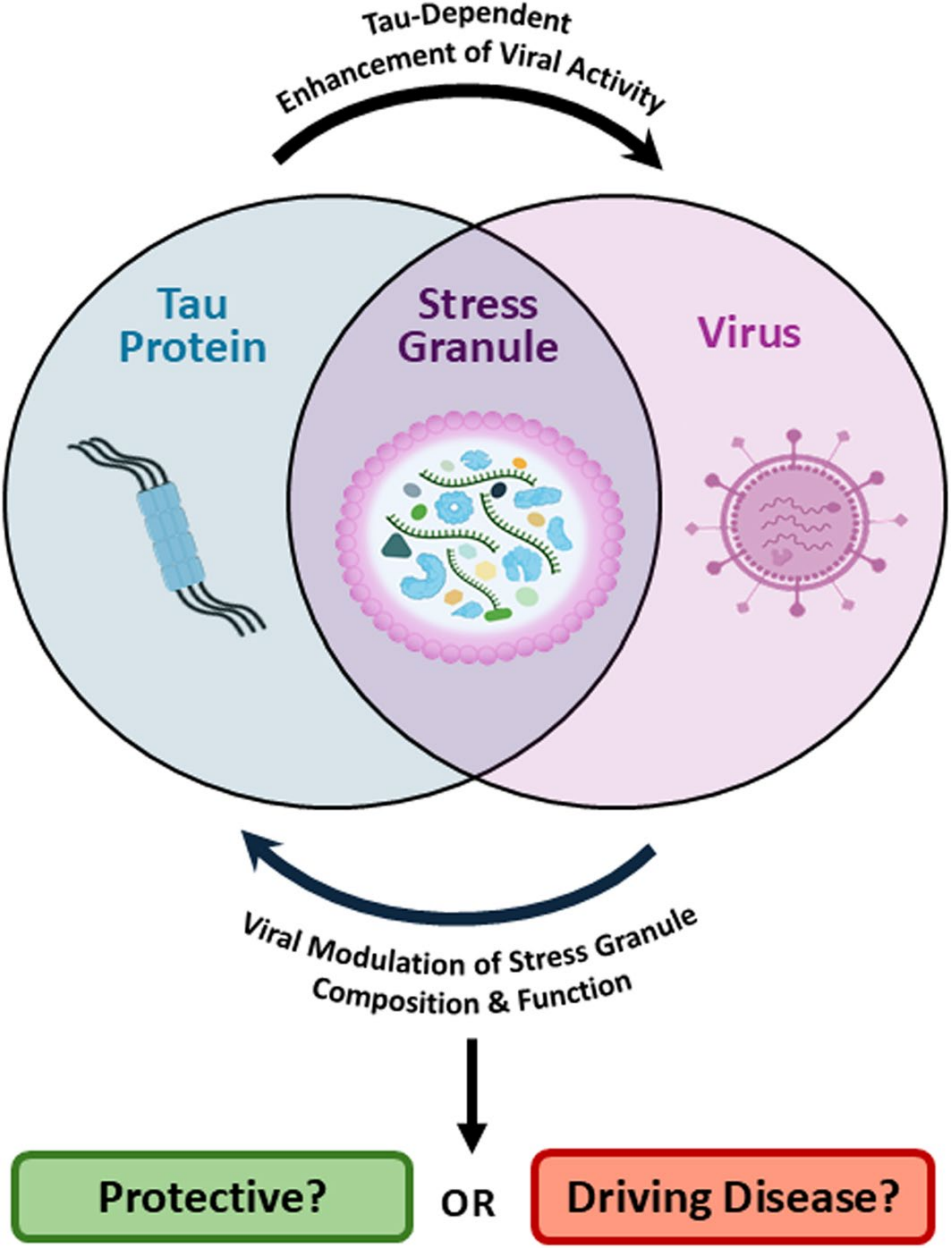

**Supplementary Information:**

The online version contains supplementary material available at 10.1186/s40035-026-00538-4.

## Introduction

Modulation of tau expression and function is often associated with viral infection. While tau aggregation and loss of function are hallmarks of several neurological diseases, the preceding events that precipitate these changes remain unclear. This review explores how viruses impact tauopathies and alter cellular pathways linked to neurodegenerative pathology, with a focus on stress granules (SGs) as a central hub for these interactions.

### States of tau within the cell

Tau is a protein widely expressed throughout the body. While frequently associated with the plasma membrane and thought of as a cytosolic protein, tau can also be found within nuclear speckles [1, 2], as well as being secreted from cells [3, 4]. Human tau protein is encoded by *MAPT* (microtubule-associated protein tau) gene, which is located on chromosome 17q21 and is made up of 16 exons [5]. Alternative splicing at exons 2, 3, and 10 causes changes in the number of N-terminal inserts as well as microtubule-binding repeats in six different isoforms of tau [5]. The number of N-terminal inserts varies from 0N to 1N to 2N, and the microtubule-binding repeats vary from 3R to 4R [5]. Thus, the six different isoforms include 3R0N, 3R1N, 3R2N, 4R0N, 4R1N, and 4R2N, with protein sizes of 352–441 amino acids [6]. Despite these differences, each tau isoform consists of four parts: the N-terminal domain, the proline-rich domain (PRD), the microtubule-binding domain (MTBD), and the C-terminal domain [6]. In healthy human brains, the 3R and 4R isoforms exist in a 1:1 ratio, but in disease states this ratio can shift, favouring some isoforms over others [7]. The human DEAD-box (DDX) proteins destabilize splice sites, resulting in alternative splicing of specific tau exons in a helicase-dependent manner [8]. Given that alternative splicing leading to *MAPT* exon 10 exclusion gives rise to 3R forms of tau [9], DDX5-mediated stimulation of *MAPT* exon 10 splicing shifts the typical ratio of tau 3R and 4R isoforms[8]. Indeed, proteomics analysis of Alzheimer’s disease (AD) brain tissue has demonstrated that DDX proteins exhibit the highest increased expression in AD, implying DDX protein activity in driving neurodegeneration [10].

Moreover, several post-translational modifications (PTMs) can occur once tau has been translated, including phosphorylation, truncation, nitration, glycation, glycosylation, ubiquitination, and polyamination [6], many of which are altered in disease states.

The complicated diversity of tau isoforms and modifications is linked to its cellular distribution patterns. For instance, tau found in the neuronal soma shows elevated levels of phosphorylation, while there is a progressive decrease in phosphorylation as tau approaches the axon’s growth cone [11]. The locations of phosphorylated tau proteins within cells are determined by their distinct patterns of phosphorylation upon synthesis, rather than by changes in phosphorylation during intracellular transit [12–14].

Tau fragments induce both cytotoxic and apoptotic processes, as well as driving neurological deficits. The extent of dysfunction induced by proteolytic tau fragments varies based on the specific fragment (reviewed by Boyarko and Hook [15]). Both tau fragments and their oligomers interact with a wide range of cellular pathways to promote toxicity. Aggregation of oligomers into neurofibrillary tangles (NFTs) has long been a hallmark of AD pathology, primarily composed of truncated forms of tau [16]. Caspase 2-mediated cleavage of tau leads to the generation of a toxic NFT (tau_1–314_), which is elevated in the brains of AD patients compared to elderly people without cognitive impairments [17]. In addition, the N-terminal tau_1-401_ fragment is used as a cerebrospinal fluid (CSF) biomarker for AD [18]. Thus, tau aggregation is directly tied to its propensity to drive neuropathology.

#### Cellular events driving tau aggregation

Tau proteins are continually phosphorylated and dephosphorylated to maintain regulation of microtubule assembly. However, if the balance is tipped in favour of phosphorylation, the affinity of tau for microtubules declines, leading to increased cytosolic tau that is more prone to aggregation [19, 20]. The capacity of tau to stabilize microtubules is compromised by its abnormal phosphorylation, which can remove other microtubule-associated proteins (MAP) and exacerbate microtubule destabilisation [21]. The combined impacts of kinase activation and phosphatase inhibition result in the hyperphosphorylation and improper folding of cytosolic tau. This has been supported by the observation that protein phosphatase 2A (PP2A) interacts with microtubules and tau, and that this interaction is diminished in the brains of individuals with AD [22, 23]. Several studies have suggested that tau propagation in the brain might occur in a manner similar to prion transmission. This phenomenon, known as tau seeding, occurs when abnormal tau from one cell affects native tau in another, leading to the formation of new tau aggregates, and is backed by numerous experimental studies [24–26]. Together, this results in the spread of tau aggregates.

According to Hernández et al. (2023), tau self-aggregation can also occur through ionic and/or hydrophobic interactions. Addition of negative charges through phosphorylation may affect protein–protein interactions, which in turn may favour the progression of tau aggregation [27]. Microtubule-binding repeats are the primary domain of tau that is involved in its self-aggregation [27]. Additionally, post-translational acetylation of lysine residues promotes tau aggregation [28]. However, not all tau proteins can self-aggregate. A recently discovered novel tau isoform generated by retention of intron 12, called w-tau, does not aggregate. This isoform is different from other isoforms in that it is devoid of exon 13 and contains an 18-amino-acid sequence corresponding to intron 12 following exon 12 [29]. Healthy people have higher brain levels of w-tau than AD patients, suggesting a possible treatment strategy to address tau aggregation toxicity [29].

Heat shock proteins (HSPs) play a crucial role in various cellular maintenance tasks, including proper folding of newly created polypeptides, reconfiguration of unstable proteins, assembly of protein complexes, elimination of misfolded proteins, and disassembly of protein aggregates [30–33]. HSPs are vital for preserving normal physiological conditions of tau protein and preventing abnormal phosphorylation and buildup of tau, thus playing a role in the pathological processes associated with tau protein in AD [34]. A number of molecular chaperones, such as HSP70 and HSP90, are known to associate with the microtubule-binding region of tau and assist in maintaining its solubility [35]. Specifically, HSP90 stabilizes oligomeric tau species while preventing the formation of high-molecular-weight aggregates and fibrils [36]. Inhibition of HSP90 results in decreased ptau levels in tau-overexpressing HeLa cells, suggesting that HSP90 plays a critical role in driving tauopathy [37]. Similarly, HSPB1 alleviates tau-related pathology in vivo [32, 38]. Another study assessed the specific mechanisms by which HSPB1 affects tau aggregation, and it found that HSPB1 intervenes early in tau fibril formation by interacting with prefibrillar species [33]. Thus, a loss of HSP activity can have dire consequences leading to tau accumulation.

Similar to the prion-like templated aggregation of tau, trans-neuronal spreading of misfolded tau proteins has been implicated as a hallmark of tau aggregation in neurodegenerative diseases [39]. Expression of human tau (hTau) protein carrying pro-aggregant mutations increases tau phosphorylation and misfolding while also causing astrocyte reactivity in mouse brain [39]. Additionally, the pro- and anti-aggregant conformations of htau are distributed identically in neurons of the entorhinal cortex (EC) and accumulate in the outer mononuclear layer near their axon terminals [39]. In AD patients, the EC is the first brain region to show degeneration, primarily impacted by tau buildup, which significantly affects memory and cognitive functions [40]. During early stages of AD, the EC undergoes degeneration, leading to cell death of 60% of layer II neurons [41]. Furthermore, this region is among the first to exhibit the accumulation of Aβ plaques, along with the development of NFTs as part of the normal aging process [42, 43].

### Diseases characterized by tau inclusions

Tau aggregation is associated with numerous diseases referred to as tauopathies, which include AD, frontotemporal dementia (FTD), frontotemporal dementia with parkinsonism-17, Pick disease (PiD), progressive supranuclear palsy (PSP), argyrophilic grain disease and corticobasal degeneration and select cases of amyotrophic lateral sclerosis (ALS) [6]. Many viral infections also result in tauopathy (discussed below). Tauopathies can be divided into molecular categories based on the distribution of tau isoforms [6] (Box 1). Moreover, research has suggested that assembled tau conformers may play a role in influencing the patterns of spread in the brain, much like prion strains [24, 44, 45]. Thus, the deposition of specific tau conformations helps explain the differences in neuropathological and clinical presentation within human tauopathies [46].

Box 1. Differentially expressed tau isoforms in neurodegenerative diseases Primary tauopathies can be classified according to the predominant tau isoforms present in their cytoplasmic inclusions: 3-Repeat (3R), 4-Repeat (4R), and 3R + 4R (termed PART) [287]. *MAPT* mRNA can be spliced to produce six distinct isoforms, featuring three or four microtubule-binding repeats (3R or 4R) [24]. The difference between 3R and 4R tau isoforms relies on the presence or absence of *MAPT* exon 10, which codes for the second repetition in the microtubule-binding region of the protein [288, 289]. The 3R and 4R tau isoforms are balanced in healthy adult human brains, but exhibit selective increases and accumulations of certain isoforms in various neurodegenerative conditions [288, 289]. Age- and NFT-related tauopathy dementia is linked to 3R and 4R tau, whereas PiD is linked to 3R tau. Progressive supranuclear palsy, corticobasal degeneration, argyrophilic grain disease, and globular glial tauopathy are correlated to 4R tau [6].

#### Tau in AD

AD is the most common neurodegenerative disease, affecting 50 million people around the world and that number is expected to increase to 152 million by 2050 [47]. AD is normally characterized by memory decline along with other symptoms including visuospatial abnormalities, navigation difficulties, executive problems, language disturbance, impaired daily life activities, and personality changes [47, 48].

Neuropathologically, amyloid and tau abnormalities may be present decades before structural changes start and symptoms appear in patients [49]. Senile plaques composed of aggregates of β-amyloid are found extracellularly (reviewed elsewhere [50]), whereas phosphorylated tau-associated NFTs are found intracellularly [49, 51]. In addition to amyloid plaques and NFTs, neuropil threads and tau tangles, reactive microglia and astrocytes, eosinophilic Hirano bodies, granulovacuolar degeneration, and cerebral amyloid angiopathy may also be present [49]. The presence of these abnormalities leads to synaptic and neuronal loss, resulting in characteristic gross neuroanatomy changes, such as enlarged sulci and shrinking gyri [49]. Currently, there are no known causes for AD pathology, but certain risk factors, such as aging, genetic factors, head injuries, vascular diseases, infections, and environmental factors can contribute to the disease [47].

AD pathology is associated with hyperphosphorylation and aggregation of all forms of tau isoforms [52]. Tau is predominantly present in the axon compartment of neurons. Under pathological conditions, tau detached from axonal microtubules can abnormally localize to presynaptic and postsynaptic compartments of the neuron and induce synaptic dysfunction through reduced synaptic mobility and altered release of presynaptic vesicles [53, 54]. Hyperphosphorylated tau also accumulates in the somatodendritic compartment of AD neurons, and its abnormal localization in dendritic spines may disrupt glutamate receptor trafficking and result in synaptic dysfunction [6]. Synaptic loss is predominantly caused by tau damage to the synaptic compartment, which can spread from one neuron to another across the brain [6]. This is clinically observed as cognitive decline in AD patients [55]. A recent study has revealed an interaction between tau and Ras GTPase-activating protein-binding protein 2 (G3BP2), a fundamental element of SGs, in the brains of AD patients when compared to control brains [56]. The binding of G3BP2 to tau occurs independently of the formation of NFTs in the AD brains and in neurons, with G3BP2 binding occurring only after tau release from microtubules [56]. A significant increase in tau pathology was detected upon the loss of G3BP2 in a human neuron-based tau seeding assay and in human cerebral organoids, indicating that G3BP2 serves as a protective barrier against tau aggregation in tauopathies [56].

Tau deregulation has an impact on genomic stability. A dangerous kind of DNA damage linked to aging is DNA double-strand breaks (DSBs) [57]. DSBs are the most common type of damage and can result in cell death if left untreated. DSBs are caused by a variety of endogenous biological activities in cells [57]. In AD patients, many DSBs were observed to colocalize with phosphorylated tau in neurons [57]. Under normal circumstances, cytoskeletal proteins like microtubule polymers contribute to DNA repair processes when damage occurs, by creating a connection with the nuclear membrane to facilitate the repairs. Tau deregulation in AD could have a role in the failure of microtubule reorganization to facilitate DNA repair processes. On the other hand, severe DNA damage might lead to microtubule disintegration and ptau buildup in the soma, which in turn worsen DNA damage and ultimately result in the death of neural cells [57].

#### Tau in PiD

PiD is an uncommon neurodegenerative disorder that is characterized by clinical symptoms of dementia, degeneration of the frontotemporal region, and presence of intracellular structures known as Pick bodies, which test positive for tau [58]. PiD acts as a neuropathological term to describe cases that show atrophy in the frontotemporal lobes and Pick body pathology as confirmed by autopsy, since a diagnosis of this tauopathy can only be made after death [58]. Importantly, PiD is recognized as the only primary 3R tauopathy. However, our knowledge about PiD is still limited, as it is a rare neurodegenerative disease [59]. Consequently, studying PiD could significantly improve our understanding of 3R-specific tau pathology and provide crucial insights into related diseases.

#### Tau in FTD

FTD, which falls in the category of frontotemporal lobar degeneration (FTLD), is a progressive neurological disorder characterized by declined language ability, changes in behavior, and deficits in executive functioning [60]. Formation of tau filaments and buildup of abnormal tau proteins are key characteristics of FTD [46]. Compared to several other neurodegenerative diseases, FTDs show a more significant genetic component due to mutations in various genes, especially the *MAPT* gene [46]. Mutations in the *MAPT* gene can disrupt normal functions of the tau protein, which can independently lead to dementia and neurodegeneration [46]. Many of these mutations result in overproduction of wild-type 4R tau, causing it to aggregate into filamentous structures [46]. It is suggested that a twisted or half-ribbon-shaped tau filament could account for the diverse behavioral manifestations observed in behavioural-variant FTD [46].

#### Tau in ALS

ALS is a motor neuron disease characterized by TAR DNA-binding protein 43 (TDP-43) pathology [61]. Several studies have shown comorbid TDP-43 and tau pathology in both AD and ALS patients [62]. Behrouzi et al*.* observed tau pathology in 24 of 41 (59%) ALS patients, 7 of 16 (44%) FTD-ALS patients, and 10 of 23 (43%) FTD patients [63]. In one study, similar tau pathology was observed in ALS patients as compared to AD, although in some ALS cases tau pathology did not exhibit as NFTs or neuropils in the frontal cortex, but as a fine granular or clumped aggregation [62, 63]. Additionally, ALS patients with cognitive impairment demonstrate a wider range of neuronal and glial tau pathology than those without cognitive impairment [64].

Neuropathologically, lower motor neurons from ALS patients have high levels of phosphorylated tau epitopes in the cytoplasm and nucleus, while those from healthy controls show nearly no immunoreactivity [62]. A study revealed a limited co-occurrence of tau and TDP-43 within the EC, amygdala, and the hippocampus of five ALS patients with cognitive impairments and one patient with AD [64]. Additionally, the presence of tau pathology is associated with a decline in nuclear TDP-43 immunoreactivity [64]. These results support the notion that alterations in tau metabolism are associated with ALS and cognitive disorders linked to ALS [64]. This supports the reasoning to consider select forms of ALS as tauopathies that share comorbid traits with TDP-43 pathology [64]. In addition, up to 57% of patients with AD also carry TDP-43 inclusions [65]. This suggests that the pathological processing of tau may potentially trigger the pathological transformation of TDP-43 [64].

## Main text

### Tau’s multifaceted roles in cell biology

Tau is a multifaceted protein that interfaces with many cellular pathways. While best known for its role within the microtubule network, it also plays essential roles in genomic stability, RNA metabolism, and SGs as reported recently.

#### Cellular architecture

Tau plays an established role in microtubule and actin cytoskeletal organization within cells, by acting as a molecular linker between these two polymer filaments [66]. Specifically in neurons, tau is involved in microtubule stability through regulation of axonal growth and transport [67, 68]. Tau does not actually stabilize axonal microtubules; instead, it is concentrated in the labile segment of the microtubule, facilitating its assembly while restricting the attachment of true stabilizers like MAP6 [69]. This allows the labile segment to grow to significant lengths without stabilization [69], thereby maintaining a dynamic microtubule component in the axon throughout the neuron’s lifespan [69]. If tau becomes hyperphosphorylated, it is unable to bind to microtubules due to conformational changes [70]. Disengagement of tau from interaction with microtubules increases its propensity to form tau oligomers, which can aggregate into NFTs [71, 72].

#### Genomic stability

Tau has a protective effect on genomic integrity when neurons are exposed to cellular stress. For instance, neurons exposed to heat shock or oxidative stress exhibit a transient increase in dephosphorylated tau within the nucleus, which has a protective effect on DNA [73]. Nuclear speckles of dephosphorylated tau are evident following heat shock and could bind genomic DNA [73], likely via its PRD and MTBD [74]. In this heat-shock model, tau-deficient neuronal cells had a greater amount of DNA breaks as measured by the Comet assay, which could be rescued by over-expression of tau (with or without a nuclear localization tag) [73]. Similarly, under conditions of hyperthermia, tau-knockout mice exhibit more neuronal DNA damage in comparison to wild-type controls, further demonstrating the protective role of tau in DNA damage response (DDR) [75]. However, hyperphosphorylated tau is unable to protect DNA from damage or participate in the DRR [76]. Cytogenetic analysis further confirmed that patients harboring *MAPT* mutations (including P301L and V363I) carry a greater load of copy number variants and exhibit aneuploidy in their peripheral blood mononuclear cells and primary skin fibroblasts, leading to genomic instability characterized by non-allelic homologous recombination [77]. Thus, native tau plays a role in maintaining genome integrity.

Moreover, in AD, tau co-localizes with the DNA damage marker phosphorylated histone 2AX (γH2AX), which marks sites of DSB [57]. In primary mouse cortical neurons treated with the DNA-damage-inducing drug Etoposide, a temporal shift in tau localization occurs; first, non-phosphorylated tau interacts with perinuclear microtubules and subsequently phosphorylated oligomeric tau accumulates surrounding the nucleus [57]. Tau knockout in this model accentuates DNA damage in neurons [57]. Additionally, inhibition of microtubule polymerization with 5HPP-33 under DSB conditions causes excessive accumulation of insoluble ptau in the cytoplasm, similar in presentation to NFTs [57]. It is postulated that tau is involved in DDR by facilitating the movement of microtubules, and that abundant ptau within the cytoplasm may lead to depolymerization of microtubules and inhibition of nuclear cytoplasmic transport, thus impeding DDR [57]. DNA damage has also been shown to lead to tau phosphorylation through activation of checkpoint kinases (Chk1 and Chk2) in both a transgenic *Drosophila* model and in in vitro kinase assays [78]. Work by Frost et al*.* shows global heterochromatin relaxation due to DNA damage in tau-transgenic *Drosophila* and mice and in AD patient brains [79]. This phenomenon is related to an enriched expression of pluripotency-associated genes such as *POU1F1*, *NOG*, and *NR5A2 *[79], as well as the expression of endogenous retroelements [80]. Thus, tau is an indispensable component in maintaining genomic stability, while DNA damage favours a disruption in tau activity.

#### RNA metabolism

Apart from the role in DDR, nuclear tau also maintains ribosomal RNA (rRNA) expression through relocalization to the nucleolus in response to stress [81]. Bou Samra et al*.* found that downregulation of tau expression reduces recruitment of upstream binding factor (UBTF) to rDNA (ribosomal DNA) loci, leading to decreased rDNA transcription, lower quantities of 45 S pre-RNA, and genomic instability. This highlights a role for tau in the regulation of rRNA synthesis [82]. Therefore, a reduction in ribonucleotide pool metabolism in cells lacking tau might be explained by diminished rRNA synthesis [82].

#### SGs and protein synthesis

SGs are dynamic, non-membrane-bound cellular compartments located within the cytoplasm [83]. SGs are also referred to as ribonucleoprotein granules (RNP) because they are made up of proteins and RNA condensates, formed through liquid–liquid phase separation (LLPS) [84, 85]. SG formation is a protective mechanism launched to combat various forms of cellular stress, leading to changes in protein translation priority [86]. Certain triggers leading to SG formation include translational arrest, tau-induced lysosomal damage, metabolic demands, or polysome disassembly [87]. On a larger scale, cellular stresses that invoke the formation of SGs include UV radiation, oxidative, osmotic, and heat stress, as well as viral infection [83, 87–89].

The general architecture of a mammalian SG includes a stable core surrounded by a dynamic shell-like structure, where proteins and RNA condense to form a dense liquid phase within a dilute liquid phase [84, 85]. The components of the SG spontaneously separate into either a dilute phase or a dense phase, which coexist stably [83]. To regulate LLPS, the cell will utilize PTMs to efficiently regulate the assembly and disassembly of these biomolecular condensates [87]. Proteomic analysis of SGs has shown a relatively stable inner core predominantly made up of various RNA-binding proteins (RBPs), as well as ATP-dependent helicases and protein remodelers [83, 85]. RBPs make up over half of all components within SGs [90] (Table S1). The fluid nature of SGs allows for the movement of proteins and RNA between the condensate and the surrounding cytoplasm [87]. Regulation of SG assembly and disassembly is governed by PTMs, such as phosphorylation, methylation, acetylation, ubiquitination and poly-ADP-ribosylation, and modification of key SG proteins by small ubiquitin-related modifiers [87].

An important cellular event precipitating SG formation is lysosomal damage. Calcium signaling mediates the initiation of SG formation in response to lysosomal damage [91]. Upon calcium leakage from damaged lysosomes, ALIX is activated by calcium, promotes the association of the innate immune sensor protein kinase R (PKR) with its activator PACT, which eventually leads to the phosphorylation of eIF2α and SG formation [91]. The activation of PKR upon lysosomal damage is not dependent on the detection of double-stranded (ds)RNA by PKR, but rather relies on its endogenous activator PACT [91] (see further discussion below). Galectin-3 (Gal3), a β-galactoside-binding cytosolic lectin, coordinates the autophagic responses to lysosomal injury and is essential for the effective recruitment of ALIX to damaged lysosomes [92]. HeLa cells expressing Gal3 showed a significant increase of ALIX on immunoprecipitation-purified lysosomes, whereas Gal3 KO HeLa cells did not show this increase, and only a minimal rise in lysosomal ALIX was observed in the absence of Gal3. This finding was further validated using additional lysosomal-damaging agents like tau oligomers [92, 93].

SGs typically function to regulate the initiation of translation, serving as a vital protective mechanism in eukaryotic cells [86]. SGs promote cellular survival when exposed to a stressor, by increasing survival gene translation and decreasing housekeeping gene translation [90]. SGs are utilized as sites of aggregation for non-translating mRNAs along with repressive translation factors, selectively inhibiting protein translation [90]. When an adaptive stress response is initiated, the shuffling of protein translation begins, with housekeeping mRNAs being stalled and shuttled to SGs [87]. Conversely, mRNAs responsible for coding stress-responsive proteins, such as HSPs, are not recruited to the SGs [87]. These stress-responsive transcripts are excluded to aid the expression of proteins vital in coping with cellular stress [87].

**Fig. 1:**
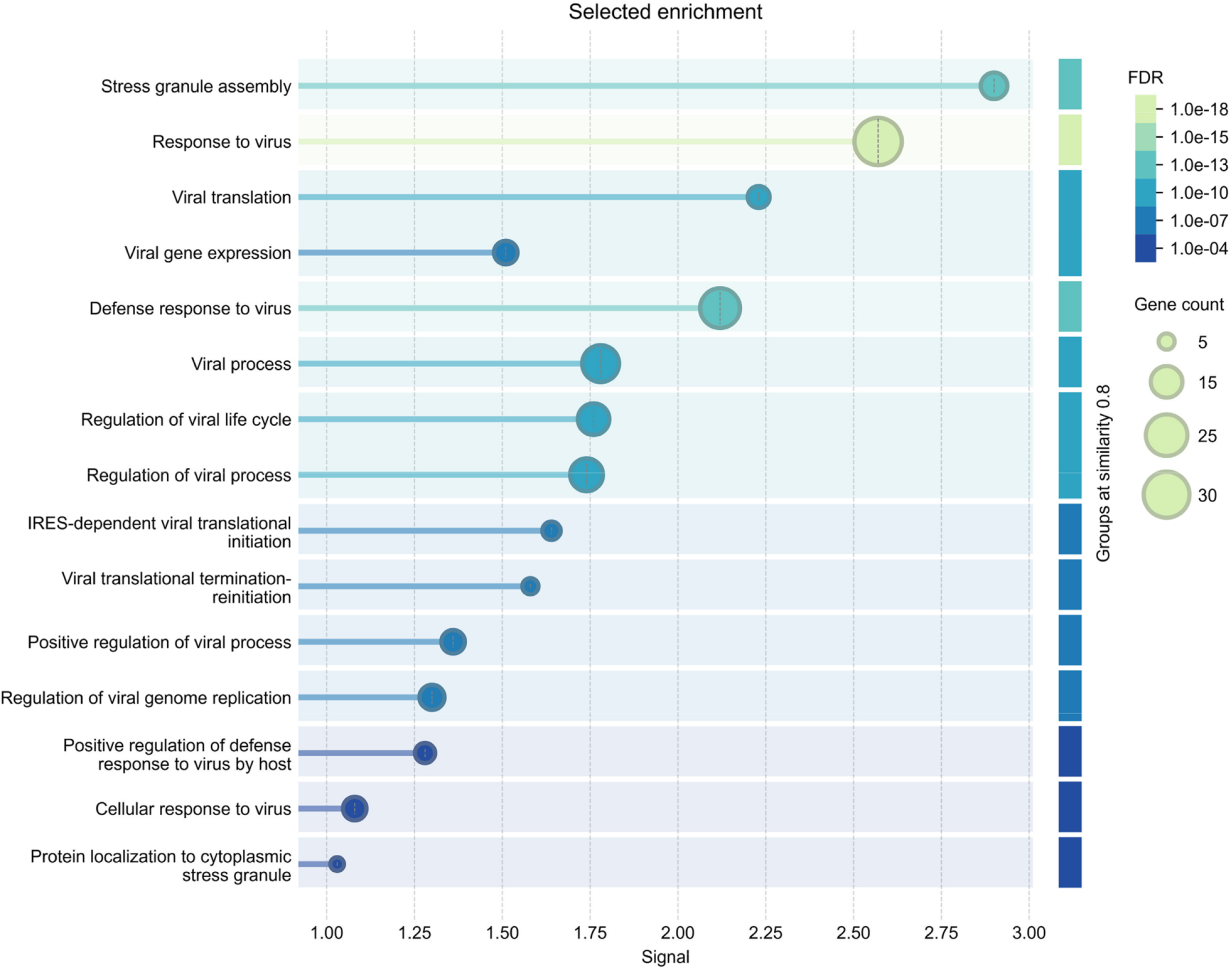
Enrichment of viral control pathways associated with the stress granule protein network. STRING analysis of Functional Enrichment Visualization of Biological Process (Gene Ontology) of 458 stress granule-associated proteins identified through the Mammalian Stress Granules Proteome (https://msgp.pt/) database. Listed are the top processes associated with viruses, as compared to the strongest signal, “stress granule assembly” within the network. The false-discovery rate (FDR) significance is represented by the colour map along with the gene count within the defined terms. Processes are sorted by signal strength, representing the weighted harmonic mean between the observed/expected ratio and -log(FDR)

**Fig. 2:**
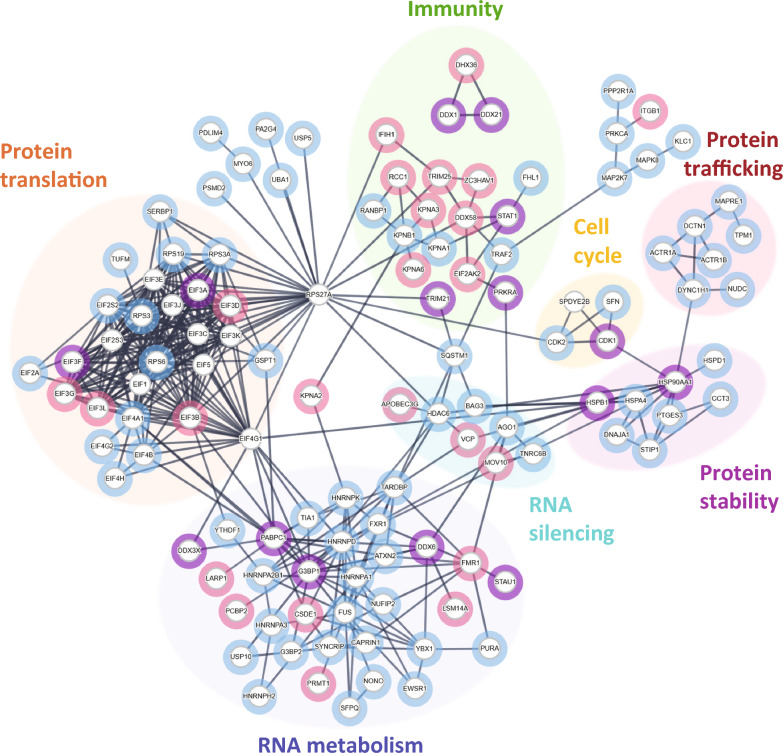
Identification of virus-associated and tau-associated proteins and pathways within the stress granule protein network. Interactome consisting of stress granule proteins known to have a functional association with tau and viruses. Proteins present in stress granules, identified through the Mammalian Stress Granules Proteome database, were categorized based on known tau interaction or viral association. A payload list was generated to colour the categorized nodes: tau interaction (blue), viral association (pink), and proteins that have dual functionality relating to both tau and viruses (purple, 15 proteins). The nodes were arranged into clusters based on dominant cellular processes. Highlighted clusters were related to immunity, protein translation, RNA silencing, RNA metabolism, protein stability, protein trafficking and regulation of cell cycle

A secondary function of SGs is to limit the interactions between the internal contents with the bulk cytosol, reducing their reactions [94, 95]. SGs modulate signalling pathways through sequestration of critical components of signalling pathways such as mTORC1, TRAF2, and RACK1 [96–98]. This functionality can be an essential survival mechanism during viral infection, as SGs will gather and activate antiviral proteins such as RIG-I, PKR, and RNaseL, enhancing innate immune responses [99–101]. Figure 1 highlights the SG pathways that are geared towards viral infection (for a full list, see Table S2). Surprisingly, tau-interacting proteins are often key components of the antiviral machinery in SGs (Table S3). Figure 2 highlights the distribution of SG proteins interacting with tau and virus-associated processes and their functional implications. Here, we highlight 15 proteins (Fig. 2, purple nodes) that putatively have dual functionality in mediating both tau and antiviral processes, yet many of these associations are not currently described in the literature. Below we will discuss how SG proteins control tauopathy and virus replication.

#### Tau consolidation into SGs

There is substantial evidence showing that tau is associated with SGs [51]. One piece of evidence is that tau oligomers, rather than tau fibrils, co-localize with RBPs including eukaryotic initiation factor 3 subunit B (EIF3η), T-cell intracellular antigen 1 (TIA1), ubiquitin-specific protease 10 (USP10), and poly(A)-binding protein (PABP) [102]. There is strong evidence supporting a connection between SGs and tau [102]. RBPs such as TIA1, EIF3η, and PABP exhibit a greater ability to co-localize with tau inclusions that are triggered by tau oligomers compared to those induced by tau fibrils [102]. TIA1-positive SGs are associated with tau oligomers, but not with fibrils, in the neuronal soma in the hippocampal CA3 region and the lateral EC [103, 104]. Phosphorylated tau increases the formation of SGs, while acetylated tau is less likely to co-localize with SGs [105]. Most studies indicate a relationship between tauopathy and SGs; however, some have reported contradictory findings [103].

##### eIF3 complex

The eIF3 complex is made up of multiple subunits that attaches to the 40S ribosomal subunit and is involved in various stages of the translation initiation process. The eIF3 complex in mammals consists of 13 different subunits [106]. The subunits eIF3A and eIF3F are linked to neurodegenerative diseases and viral infection, respectively (Fig. 2, purple nodes). eIF3A, a component of various types of SGs [107], forms local aggregations into non-membrane structures in neutrophils from AD patient blood samples [108]. Alternatively, eIF3F can inhibit human immunodeficiency virus (HIV) replication by preventing the maturation of viral RNAs [109]. Yet, how eIF3A and eIF3F modulate tauopathy, especially in the context of infection, remains unclear.

##### TIA1

TIA1 is an RBP that promotes SG formation [90]. TIA1 has been shown to co-localize with tau in the brains of patients with tauopathies [110], as well as in murine models of tauopathy [111]. In vitro, TIA1 co-partitions with tau under physiological conditions, and induces generation of tau oligomers. The generated tau oligomers are highly neurotoxic in primary hippocampal mouse neurons [112]. TIA1 tends to co-localize predominantly in the cytoplasm with hyperphosphorylated tau in SH-SY5Y neuronal cells, but not with SG marker G3BP1 [113]. In the absence of tau, TIA1 remains primarily in the nuclei of neurons from tau^−/−^ mice[111]. Using primary hippocampal neurons from either wild-type or tau^−/−^ mice, Vanderweyde et al*.* found that tau promotes somatodendritic localization of TIA1 [111]. Specifically, expression of mutant P301L tau leads to larger, but fewer TIA1^+^ SGs compared to wild-type tau [111], an observation commonly seen with disease-associated mutations in RBPs [114, 115]. Tau over-expression is associated with reduced protein synthesis, due to SG formation [111, 116, 117]. TIA1 knockout in neuronal cells leads to decreases of misfolded tau, whereas an increase of TIA1 leads to tau misfolding and ultimately neurodegeneration [111]. Overall, the interaction between tau and TIA1 enhances SG formation and tau misfolding, which in turn leads to neurodegeneration [51].

Zinc may be an important aspect underlying the effects of TIA1 on pathogenic tau condensation. Zinc is the most abundant trace metal in the brain but exhibits neurotoxic properties at high concentrations. When Zn^2+^ binds the RNA recognition motif 2 domain of TIA1, it promotes TIA1 multimerization and acts together with nucleic acids to induce LLPS, thus regulating the dynamic recruitment of TIA1 into SGs [118, 119]. Similarly, treatment of neuronal cells with zinc promotes LLPS of tau and increases the interaction of tau with G3BP1 in SGs [120]. Thus, physiologic changes in zinc concentration and localization (such as in conditions of cellular stress and viral infection [121]) may impact tau deposition in disease states. Given that many neurodegenerative diseases are characterized by either zinc excess or deficiency [122, 123], it remains to be determined whether zinc sequestration is an adaptive response against tauopathy.

More evidence for the role of SGs in AD is that they have different interactions with phosphorylated versus acetylated tau [105]. SG proteins are significantly linked to tau pathology in the brain tissues of patients with AD, frontotemporal lobar degeneration with ubiquitin-positive inclusions (FTLD-U) and ALS, and of relevant animal models [81, 111]. The TIA1 protein interaction network identified in the brains of both wild-type (WT) and tau knockout mice suggests a close interaction between TIA1, other RBPs, and hyperphosphorylated tauopathy [111]. TIA1 influences the distribution of internalized tau protein, enables tau misfolding, promotes the localization of pathological tau into granules, and decreases the degradation rate of tau, whereas overexpression of TIA1 leads to a reduction in the overall tau protein levels [111]. Evidence from neuronal cultures demonstrated that deletion or knockdown of TIA1 hinders tau misfolding and its associated toxicity [111]. Reducing TIA1 levels is linked to improved neuronal survival and memory, and an extended lifespan [103]. In addition, extracellular tau affects the distribution of TIA1, speeding up the formation of SGs, and tau internalization disrupts the typical disassembly process of SGs [105]. Thus, the diverse roles of TIA1 imply that its reduction may provide a degree of protection against tauopathy; however, the full mechanisms underlying these multiple pathways are still not well understood [103].

##### USP10

USP10 is another protein involved in SG formation, which is found to colocalize with tau aggregates in the neurons of AD patients [124]. In HT22 neuronal cells with USP10 depletion, TIA1^+^tau^+^ SGs fail to form under stress conditions [124]. However, the mutant variant USP10^C424A^ (lacking deubiquitinase activity) retains the ability to form tau-positive SGs, suggesting that the deubiquitinase function of USP10 is not essential for the formation of tau/TIA1-SGs [124]. Together, key SG proteins TIA1 and USP10 cooperate to facilitate pathogenic tau aggregation in neurons.

##### PABP

PABP associates with the 3′ poly(A) tail of mRNA, facilitating poly(A) lengthening and translation termination, as well as preventing nonsense-mediated mRNA decay (NMD) [125]. PABPC1 (Fig. 2, purple node) is an essential component of SG formation and is concentrated at sites of high mRNA [126]. Specifically, when PABPC1 binds to both the poly(A) tail and eIF4G, it stabilizes circularized mRNAs, thus preventing NMD-mediated mRNA degradation [125]. The interaction of its RNA-recognition motifs (RRMs) with the poly(A) tail blocks interaction with importins, preventing PABPC1-bound mRNA from going back into the nucleus [125].

In the prefrontal cortex of females with AD, mRNA transcripts are more likely to harbour retained introns compared to their male counterparts with AD [127]. Moreover, the increased intron retention is associated with lower mRNA abundance, pointing to NMD activity [127]. Indeed, proteomic analysis revealed that these intron-containing transcripts are enriched for PABPC1-binding motifs [127], which likely protect them from NMD. Notably, intron retention also causes the formation of truncated aggregation-prone Tau11i [128]. Given that RNA splicing is disrupted by tau aggregation [129], the accumulation of Tau11i may cause a feed-forward loop generating aberrant mRNA moieties in tauopathy.

##### Staufen-1 (STAU1)

STAU1 is a protein that binds RNA and features four double-stranded RNA-binding domains (dsRBDs) [130, 131]. STAU1 is critical for RNA processing, contributing to staufen-mediated mRNA decay and formation of SGs (Fig. 2, purple node) [132, 133]. STAU1 regulates neuronal differentiation and is implicated in the development of neurodegenerative conditions such as spinocerebellar ataxia, ALS, and FTD [134–136]. Notably, STAU1 is overexpressed in AD models, suggesting a potential link to tauopathy [134, 137, 138]. STAU1 facilitates tau phosphorylation by activating GADD45B and p38 MAPK signaling [137]. Silencing either *STAU1* or *GADD45B* leads to substantial decreases in the pS396 and pT181 phosphorylated forms of tau in SH-SY5Y cells [137]. Moreover, STAU1 is essential for retroviral virion assembly [139, 140]. STAU1 interacts with the HIV Gag protein, facilitating Gag oligomerization and viral RNA encapsidation, initial steps in virion assembly [140]. STAU1 also binds to the Gag protein of endogenous retrovirus-K (ERVK), enhancing virion production in HEK 293 T cells [139]. An accessory protein of ERVK called Rec, colocalizes with STAU1 in SGs in cells that express viral RNA and are treated with sodium arsenite to induce a stress response [139]. This suggests that STAU-1 is both a viral co-factor and an intermediate in pathogenic tau phosphorylation.

##### Cyclin-dependent kinase 1 (CDK1)

CDK1 has transitioned from being primarily regarded as a cell cycle regulator to a key contributor in viral infections, cellular stress responses, and neurodegenerative diseases, such as AD. In neurons, HDAC4 inhibits the activity of CDK1, thus preventing cell-cycle progression and inappropriate re-entry of neurons into the cell cycle [141]. However, CDK1 has other cellular roles, including aiding in the formation of mature SGs (Fig. 2, purple node) [142]. CDK1 phosphorylates the 5′TOP mRNA-binding protein LARP1, thereby positively regulating the translation of 5′TOP mRNAs, impacting the production of ribosomal proteins and other translation factors [143]. Additionally, CDK1 plays an important role in tau phosphorylation [144]. The CDK1 inhibitor, Olomoucine, reduces tau-induced neurotoxicity in *Drosophila* models of tauopathy, similar to the effects observed with genetic decrease of CDK1 function [145, 146]. Furthermore, CDK1, along with related cyclins, activates viral DNA synthesis and facilitates the replication of HIV-1 and Herpes simplex virus 1 (HSV-1). Therefore, several antiviral cascades converge on CDK repression to limit viral DNA synthesis [147].

#### Tau facilitates phase separation

The formation and stabilization of SGs and other RNA granules seem to rely on the biophysical mechanisms of LLPS and protein aggregation [148]. Similar to other RBP proteins, tau demonstrates a propensity to phase separate with RNA. Indeed, tau hyperphosphorylation is thought to encourage complexes of tau, RBPs (including TIA-1) and RNA to undergo LLPS and impede SG disassembly [105]. Moreover, cellular internalization of extracellular hyperphosphorylated tau enhances the effect of SG driver drugs like salubrinal, and delays the clearance of SGs [105]. Chronic tau deregulation likely impacts cellular health and neuropathological outcomes. During temporary stressful situations, SG components rapidly assemble and disassemble, resulting in dynamic structures influenced by the biophysical principles of phase separation [149, 150]. Over prolonged periods, such as those experienced during chronic stress, certain SG proteins can evolve into highly stable amyloid-like forms, similar as the protein aggregates seen in neurodegenerative diseases [151, 152].

The roles of LLPS and protein aggregation are critical aspects that highlight the significance of SGs in the pathophysiology of neurodegenerative diseases, particularly in tauopathies [148]. Condensation of tau-containing droplets and SGs may further permit the misfolding and aggregation of tau into the irreversible fibrils associated with neurodegeneration. One major question that remains is the nature of RNAs that tau associates with during these processes. In the following section, we will discuss whether viral RNAs (and other components) play a role in tau phase separation under stress conditions.

### Tau as a partner in viral infection

#### Tauopathy promotes the expression of genome-encoded transposable elements (TEs)

Accumulating evidence supports a role for tau in the regulation of TEs [153–155]. As mutagens and potentially mobile DNA sequences within the human genome, TEs represent both a risk to genomic integrity and a potential modulator of gene expression patterns [156]. Moreover, TE-encoded proteins play roles in neurodegeneration (recently reviewed in [157, 158]). Experiments both in vivo and in vitro have shown that tau is associated with dysregulation of several TEs, including endogenous retroviruses (ERVs – LTR (Long Terminal Repeat) retrotransposons), non-LTR retrotransposons (Long Interspersed Nuclear Elements [LINEs]) like L1 elements, Short Interspersed Nuclear Elements [SINEs] like Alu and SVAs), and DNA transposons [153, 157, 159]. TE-derived miRNAs are also differentially expressed in AD [160].

This dysregulation of TEs is likely associated with tau-mediated epigenetic changes, such as heterochromatin relaxation [79] and histone acetylation [161], potentially leading to de-repression of silenced TEs [162]. These tau-mediated changes in chromatin architecture are not on a small scale, but occur in genomic blocks, several megabases in size [161]. Modulation of heterochromatin dynamics by tau can lead to oxidative stress and subsequent DNA damage causing DNA relaxation, measured as reduced levels of H3K9me2 (dimethylated lysine 9 of histone 3) and HP1α (heterochromatin protein 1α)[79].

In AD and to a lesser extent PSP, dysregulation of expression of multiple types of TEs has been observed [154]. For example, in AD patient brain tissues, increases of ERVK-C4 (4.5-fold), ERVK-22 (3.6-fold) and ERVK-11 (2.1-fold) have been observed as compared with controls [154], indicating that multiple loci of ERVK are active in AD. The LINE (L1) and SINE (Alu) groups of TEs exhibited the most robust changes in expression in AD, with select up or down-regulation pattern for a given loci [154]. Similarly, Guo et al. found enhanced ERV and LINE transcripts in AD brains, with select TE types correlated with NFT load [153]. Using blood-based TE load as a biomarker of cognitive decline and AD, Macciardi et al*.* demonstrated that a robust enhancement of TE transcripts from LINEs and ERVs occurs prior to clinical presentation of late-onset AD [163].

Murine modeling further confirms that pathogenic tau accelerates aging-associated expression of TEs. ERVs are transcriptionally enriched in *tau*^*P301L*^ rTg4510 mice, and an enhanced DNA copy load of TEs in brain tissue was observed at 9 months of age compared to 20-month wild-type mice [80]. The enhanced TE expression in AD and *tau* transgenic mice is present with neuroinflammation, as marked by increased detection of TE-derived dsRNA by the innate immune system [162]. Specifically, reactive astrocytes in human and murine tauopathy cases express the viral RNA sensor MDA5 (melanoma differentiation–associated protein 5), and have elevated levels of dsRNA [162].

In contrast, SH-SY5Y neurons overexpressing 4R tau isoform exhibited greater suppression of TEs, compared with the 3R tau isoform or controls [154], suggesting a constitutively active effect of tau on TE activity.

Therapeutically, there is a growing effort to target TEs to improve cognitive decline. The use of reverse transcriptase (RT) inhibitors suppresses TE activity and tau-induced neurotoxicity in *Drosophila *[159]. Further, Wahl et al*.* have demonstrated that the RT inhibitor 3TC can improve cognitive function and neuroinflammation in aged mice, as well as enhancing cognitive function in a mutant P301L htau mouse model [164]. These findings suggest a protective effect of targeting TE activity in aging and tauopathy.

#### Tau pathology may facilitate intercellular viral dispersal

First described by Rustom et al*.* in 2004 [165], tunneling nanotubes (TNTs) are filamentous-actin-containing structures surrounded by cell membrane, which serve to bridge neighbouring cells [166]. Like gap junctions, TNTs allow the exchange of cytoplasmic constituents, including larger entities such as organelles and vesicles, between connected cells [166]. These dynamic structures contain tau and can elicit the transfer of tau fibrils between neurons, through engagement with actin networks [167, 168]. While tau overexpression cannot induce TNT formation, the application of extracellular htau 1N4R fibrils led to the formation of TNTs in both murine CAD cells and rat primary embryonic cortical neurons, which ultimately facilitated the intercellular spread of pathological tau via TNTs [167]. Recent data demonstrate that microglia protect neurons from pathogenic tau accumulation by using TNT to extract toxic aggregates from neurons [169].

Moreover, viruses usurp tau-containing structures emanating from the cell membrane to facilitate virus transmission [170–173]. Indeed, HIV uses TNTs to facilitate cell-to-cell spread by increasing the overall TNT numbers on the infected cell surface, and using internal and external TNT structures to transport viral particles, completely avoiding the extracellular environment, or migrating along the external bridge structure [170, 174]. This mechanism can also bypass trophic barriers to infection, as TNTs allow viral invasion of non-permissive cells, as seen in co-cultures of SARS-CoV2-infected permissive epithelial cells and human neurons (which are non-permissive to infection), where TNTs provide a route for SARS-CoV-2 spreading to neurons [175]. This may explain neurotrophic infection in tissues devoid of known viral receptors.

Viral mimicry of tau by the nucleocapsid (N) protein of a neurotropic strain of mouse hepatitis virus (MHV-strain JHM) facilitates viral interaction with neuronal microtubules [176]. Microtubule inhibitors (noscapine and nocodazole) and actin depolymerizing agents (cytochalasin D and latrunculin A) limit MHV-JHM replication in neurons [177], suggesting that targeting TNTs may limit viral dispersal in the brain. Thus, a closer examination of how tau pathology (or viral mimicry of tau) facilitates viral transmission via TNTs is warranted in neurodegenerative conditions associated with virus activity.

Although there is no current evidence for SG transport along TNTs, there is however, documentation of RBP condensates being transported along axonal and dendrite microtubule networks [178], suggesting that a similar mechanism could be in place in TNT-mediated tau trafficking.

#### Tau impacts antiviral immunity

An additional intersection between tauopathies and viruses is the modulation of immunity. A recent study has demonstrated that type-1 interferons (IFN-I; IFN-α and IFN-β) can enhance tau aggregation via poly:C (a molecular pattern that resembles viral nucleic acid) signaling, leading to a moderate increase of seeded tau aggregation as assessed by staining for hyperphosphorylated tau [179]. The tauopathy was significantly reduced by four-fold in the brainstem of P301S-tau mice lacking *Ifnar1*, compared to those able to respond to IFN-I [179]. This indicates that IFN plays a crucial role in the buildup of tau pathology in the brain [179]. Conversely, tau aggregation is a key trigger of several antiviral signalling pathways, including inflammasome activation [180] and TRIM21-mediated clearance of antibody complexes.

##### Signal transducer and activator of transcription-1 (STAT1)

STAT (Fig. 2, purple node) exists in a dormant state within the cytoplasm, but moves to the nucleus upon activation [181]. The Janus kinase (JAK)/STAT pathway is a primary signaling mechanism that mediates the actions of interferons and various cytokines [182]. The buildup of tau inhibits NMDAR expression by activating the JAK2/STAT1 signaling pathway, which impairs synaptic function and memory, further exacerbating AD pathology [183]. Reducing STAT1 expression or preventing its activation effectively alleviates synaptic dysfunction and memory deficits induced by htau overexpression in mice [183]. Moreover, STAT1 activation is critical for the prompt establishment of the antiviral response, even in the absence of cytokines [184]. Interestingly, many viruses either cause the degradation of STAT1 or inhibit its activation as part of an immune evasion strategy [185]. Thus, viral depletion of STAT1 may be a beneficial aspect in tauopathy.

##### Tripartite motif (TRIM) family members

TRIM21 is an E3 ligase that tags internalized antibody-bound complexes for proteasomal degradation while trafficking along the cytoplasmic microtubule network (Fig. 2, purple node). In addition, TRIM21 negatively regulates the formation of SGs by catalyzing K63-linked ubiquitination of G3BP1 [186]. TRIM21 is effective in depleting misfolded tau [187, 188]. TRIM21 interacts with the tau–antibody complexes internalized into the cytosol of neurons, and prevents mutant tau seeding in organotypic hippocampal slice cultures prepared from P301S tau transgenic mice and in P301S tau transgenic mice in vivo, but not in TRIM21 knock-out P301S mice [189]. This has become the basis of the therapeutic RING-Bait strategy to deplete pathogenic tau aggregates [190]. TRIM21 is also essential for protecting cells from virus infection by binding to antibody-virus complexes and interacting with viral proteins [191]. Nevertheless, enveloped viruses can evade the actions of TRIM21 by shedding neutralizing antibodies during viral entry [192]. It remains unclear how (and if) tau internalization monopolizes TRIM21 activity to impact antiviral immunity in the context of infection, specifically downstream antiviral NF-κB and IRF signalling [192].

TRIM11 also has three significant functions related to tau [193]. Firstly, it facilitates the degradation of both mutant tau and excess non-mutant tau proteins [193]. Secondly, it serves as a molecular chaperone for the tau protein, preventing its misfolding and aggregation [193]. Lastly, TRIM11 acts as a disaggregase for tau, effectively dissolving existing tau deposits, including fibrillar aggregates [193]. In mice injected with tau fibrils to promote AD symptoms, the introduction of TRIM11 via an adeno-associated viral vector results in reductions of tau pathology and neuroinflammation, as well as improved cognitive and motor abilities [193].

##### DDX family proteins

DDX proteins have been implicated in antiviral immune responses. They act as receptors for viral nucleic acids, modulating the downstream of pattern recognition receptor signaling, and regulating signal transduction related to RIG-I-like receptors (RLRs) and MAVS adaptors [194]. DDX3, DDX6 and the DDX1-DDX21-DDX36 complex have been identified as receptors for viral nucleic acids, resulting in the induction of IFN-I [194]. Several DDX family members of RNA helicases mount intrinsic and innate immune responses against viruses by sensing viral nucleic acids. In addition, some DDX proteins also play a role in the formation of SGs [194]. During viral infection, RLRs localize in SGs with viral RNA and antiviral proteins [99]. During initiation of an antiviral response, DDX6 (Fig. 2, purple node) binds to activated RIG-I, resulting in IFN induction, indicating that DDX6 may serve as a signaling enhancer for RIG-I [195].

In contrast, recent findings indicate that SG formation competes with NLRP3 inflammasomes for DDX3X, thereby inhibiting NLRP3 inflammasome activation by sequestering DDX3X (Fig. 2, purple node) [196]. When incorporated into the inflammasomes, DDX3X promotes inflammasome activation [196]. In response to poly(I:C) (a synthetic analogue of viral dsRNA) stimulation in macrophages, the NLRP3 inflammasomes also sequester DHX33 and G3BP1 (Fig. 2, purple node), inhibiting SG assembly [194]. In contrast, tauopathy, specifically phosphorylated forms of tau that retain K18 acetyltransferase activity, induces inflammasome activation through NLRP3 acetylation [180], which promotes microglial activation and is associated with cognitive impairment in 3 × Tg‐AD mice [180]. Together, tauopathy and viral activity may put differential pressure on DDX proteins and G3BP1 usage, for either inflammasome activation or SG formation.

##### PACT: Protein ACTivator of the Interferon-induced protein kinase

PRKRA (protein activator of interferon-induced protein kinase EIF2AK2; also known as PACT) demonstrates prevalent activity in the integrated stress response (ISR), specifically involved in antiviral mechanisms through modulation of interferon-stimulating genes [197]. A hallmark function of PACT is to heterodimerize and activate PKR in response to cellular stress conditions, especially viral infection. PACT becomes activated upon binding dsRNAs during viral infection [197], where it associates with STAU1 and PKR in cytosolic condensates [198]. PKR activation subsequently inhibits cellular mRNA translation and drives a pro-inflammatory response via NFκB. Several viruses have developed the ability to block PACT activity or SG formation, thus limiting PKR-mediated responses [199, 200]. Yet, a counterexample is peste des petits ruminants virus (PPRV), which takes advantage of PACT’s ability to promote SG formation, thus promoting its own replication [201]. This dichotomy suggests that modulation of PACT, PKR, and SG formation may be virus-specific and requires evaluation on a case-by-case basis. Importantly, this feeds into the fact that PKR activation drives tau phosphorylation and may be linked with the observations of PACT overexpression in AD brains [202].

#### Tau phosphorylation as a protective response to viral replication

Tau phosphorylation acts as a protective mechanism against viral replication, as neurons expressing tau proteins showed a substantial reduction of cell death when infected with HSV-1, indicating that tau phosphorylation helps prevent cell death post-HSV-1 infection [203]. ICP27 is a crucial nuclear protein from HSV-1 that promotes the expression of viral early and late genes during infection [204]. Phosphorylated tau dramatically lowered ICP27 expression by 87.40%, while the wild-type tau protein reduced ICP27 expression by 54.40%, further demonstrating that tau phosphorylation inhibits the expression of this viral protein [203].

HSV-1 and other DNA viruses are primarily detected by cGAMP synthase (cGAS), a cytoplasmic sensor for viral DNA that activates the cGAS–STING (stimulator of interferon genes) pathway, leading to the production of pro-inflammatory and antiviral cytokines, chemokines, and interferons. This pathway is thought to be upregulated in neurodegenerative disorders like AD [205, 206]. Activation of the cGAS–STING pathway triggers the activation of TANK-binding kinase 1 (TBK1), a kinase known to phosphorylate tau protein [203, 207]. This suggests a causal link between TBK1 activation and tau phosphorylation [203]. Together, this suggests that tau phosphorylation is an essential step in antiviral immunity, potentially acting as a viral restriction factor.

### Viruses as a partner in tauopathy

There is abundant literature on the association between viruses and neurodegenerative disease [157, 208–212]; however, few have shown concrete causal relationships. The challenges in developing an understanding of pathogens as a trigger of nervous system degeneration stem from dissociation in time between the triggering events and demonstrable neuropathological changes. The prodromal triggering events and chronic subclinical viral activity may precede the diagnosis of tauopathies by years, if not decades. Notably, a recent longitudinal study indicated a strong correlation between neurodegeneration (including AD) and past viral infections, with the largest effect association between viral encephalitis and AD [213].

#### Viruses that can directly promote tau pathology

##### Herpesviruses

HSV-1 is a prevalent neurotropic virus that is known to induce encephalitis and has been consistently associated with AD [214]. A study found that untreated HSV-1 infection raises the risk of dementia by 2.56 times; however, this additional risk is significantly reduced if patients receive anti-herpetic treatment [215]. HSV-1 is detected in AD with greater frequency than in control brain tissues and is more predominantly associated with amyloid plaques [216]. While tau is not required for HSV-1 replication in neuronal cells, it does cause an increase in ptau in the nucleus [217]. Wozniak et al*.* demonstrated that HSV-1 infection in SH-SY5Y cells leads to increased pathological tau phosphorylation and promotes the abnormal folding of tau [218]. SH-SY5Y cells infected with HSV-1 have also shown retention of *MAPT* exon 10, resulting in increased levels of tau 4R protein, tau hyperphosphorylation, and tau oligomerization[219]. However, the S396 ptau increase in response to HSV-1 infection is transient in hippocampal neuronal cultures in early infection (days 1–3), and can be similarly induced by the antiviral chemical acyclovir in uninfected cells [220]. In addition, HSV-1 infection induces HSP90 nuclear translocation (Fig. 2, purple node), which assists with HSV-1 protein folding and transport, playing a critical role in the early stages of HSV-1 infection [221]. This highlights that selective SG proteins, like HSPs, may be an essential machinery for SG accumulation of both tau and viral proteins.

Murine models have shown that HSV-1 infection of the trigeminal ganglia and cerebral cortex results in increased levels of hyperphosphorylated and cleaved tau proteins [222]. The presence of these neurodegenerative markers (ptau and cleaved tau) prior to insoluble tau deposits indicates that tau dysfunction occurs early in the neurodegenerative process [222]. HSV-1 infection-induced tau pathology is independent of beta-amyloid plaques, further supporting that neurotropic viral infections drive tauopathies [222]. In addition, acyclovir treatment reduces ptau levels in the brains of HSV-infected mice [223]. Recent findings have shown that HSV-1 infection also promotes neuronal tau spreading through extracellular vesicles (EVs) [224]. Based on the findings supporting the role of HSV-1 in dementia, clinical trials of anti-HSV treatment in AD are underway [225].

##### Coronaviruses and SARS-CoV-2

The SARS-CoV-2 3CL protease (3CLpro) has been shown to promote the proteolytic cleavage of 2NR4 tau into fragments, leading to tau aggregation in vitro [226]. This effect has been confirmed in SH-SY5Y neuroblastoma cells with SARS-CoV-2 infection and ACE2-humanized mice, which show significant p262tau phosphorylation [227]. It has been further proposed that long COVID is a manifestation of virus-induced tauopathy [228]. G3BP2 is a widely recognized factor that assists in the assembly of SGs within the cytoplasm for the formation of viral replication complexes during infections by certain RNA viruses [229]. A 2019 study demonstrated that the replication of SARS-CoV-2 is notably heightened in cells that are deficient in both G3BP1 and G3BP2 compared to WT cells, suggesting that G3BP1 and G3BP2 proteins share overlapping antiviral roles against SARS-CoV-2, beyond their involvement in the SG pathway [230]. In another study, SARS-CoV-2 replication was increased in G3BP2-knockdown human bronchial epithelial cells (16HBE), while the expression of the IFN-β gene was suppressed [231]. Despite the upstream activation of the RIG-I, the presence of G3BP2 inhibited RIG-I signaling [231]. Thus, G3BP2 modulates the production of IFN-I, and its interaction with TRIM25 hinders the activation of the RIG-I-like receptor signaling pathway [231]. This suggests that SG proteins, like G3BP2, may have dual impacts on tauopathy and immunity under conditions of cellular stress, such as viral infection.

##### Flaviviruses

Zika virus (ZIKV) infection induces the cytoplasmic deposition of ptau in both primary neurons and mouse brains, which is associated with immune activation in vivo, with ptau^+^ neuron clusters surrounded by activated IBA1^+^ microglial cells [232]. Moreover, ZIKV infection in cerebral organoids triggers AD-related phenotypes, including PKR activation and ptau accumulation [233]. In vivo, ZIKV infection in immunocompetent mice resulted in neuronal ptau accumulation, surrounded by pockets of activated microglia [232].

Additionally, TIA-1 and the TIA-R-related protein (TIAR), both associated with SGs, interact with viral RNAs in cells infected by the tick-borne encephalitis virus (TBEV) [234]. During the TBEV infection cycle, TIA-1 and TIAR accumulate in the cytoplasm at sites of viral replication, resulting in their depletion from SGs [234]. Overproduction of TIA-1, and probably TIAR as well, negatively impacts the replication of TBEV, as the overexpression of TIA-1–GFP led to reduced virus production at 24 h post-infection [234]. TBEV infection leads to the formation of G3BP1 SGs, but competes with SGs for TIA-1, leading to TIA-1 positioning in the locations of viral replication [234]. This is a clear case of viral modulation of SG composition, as TBEV promotes the assembly of a subcomplex comprising viral RNA, TIA-1, and TIAR at the sites of viral replication [234]. How this impacts tau in the context of TBEV infection remains to be explored.

##### Exogenous retroviruses

Human exogenous retroviruses, HIV and Human T-lymphotropic virus (HTLV), have both been implicated in tau pathology. Alterations in total tau and ptau have been extensively studied in HIV-infected individuals [235, 236], including stratification by cognitive status for those presenting with HIV-associated neurocognitive disorders (HAND). The total tau in the CSF does not significantly differ between control and HIV patients, but shows an increase in the majority of patients with HAND [237–242]. Yet, no correlation has been observed between CSF tau levels and dementia severity or brain atrophy as measured by magnetic resonance imaging [242].

In contrast, similar CSF ptau levels among HIV^+^ individuals regardless of cognitive status, may be used to differentiate HAND from AD (as characterized by enhanced ptau levels) [240, 241, 243–245], although some studies found equivalent levels of ptau in HAND and AD [246]. Blood levels of ptau are also not associated with the degree of neurodegeneration and cognitive impairment in HIV infection [247]. Neuropathology has revealed only weak ptau levels in the prefrontal cortex of < 45% of HIV cases [248]. However, Anthony et al*.* found that many HIV cases displayed increased levels of hyperphosphorylated tau in the hippocampus compared with age-matched controls [249]. This increase in hyperphosphorylated tau was most notable in individuals receiving highly active antiretroviral therapy, suggesting that HIV infection and/or anti-retroviral therapy may accelerate neuroageing within the hippocampus. Observations from an HIV transgenic rat model further support the changes in the hippocampus in HIV infection, revealing that neurodegeneration occurred in the hippocampal CA1, CA3, and the dentate gyrus and was associated with tau hyperphosphorylation and phosphorylation of tau kinases CDK5, GSK-3αβ, JNK and p38 [250]. Pharmacologically, lithium was effective in reducing ptau and pGSK-3β in a mouse model of HIV encephalitis, where HIV-infected monocyte-derived macrophages were injected into the basal ganglia of severe combined immunodeficient mice to cause neuropathology [251].

Mechanistically, the HIV protein Tat modulates tau expression [236]. In SH-SY5Y neuron cultures, HIV Tat overexpression increases ptau levels [252]. This effect can be blocked with caffeine treatment [252]. Similarly, HIV Tat transgenic mice display pathologic ptau accumulation and significant synaptodendritic degeneration at 2 months of age [253]. Several mechanisms have been proposed. One is through Tat-induced calcium dysregulation, which leads to hyperactivation and cytoplasmic retention of CDK5, resulting in aberrant phosphorylation of several downstream targets including tau and cell toxicity [254]. Another pathway involves Tat-induced upregulation of 3R tau through alterations of SC35-dependent alternative splicing of *MAPT* exon 10 caused by Tat-dependent increase of DYRK1A, a SC35 kinase [255].

HIV infection may further impact tauopathy by enhancing disease severity through several other viral proteins, including the envelope protein (gp120) and accessory protein Nef. As observed with HIV Tat, HIV gp120 can also promote hyperphosphorylation of tau and its extracellular secretion via activation of kinase regulator cGKII [256]. This is consistent with a study showing that intracerebroventricularly injected HIV gp-120 accelerated cognitive decline in transgenic tau (P301L) mice compared to wild-type controls, as measured by behavioural performance using a Y-maze [257]. Together, this suggests that HIV gp120 may accelerate tau pathology during HIV infection. In contrast, HIV Nef overexpression appears to decrease the ptau levels in primary neurons, while simultaneously enhancing the total tau load [258]. Moreover, HIV protein Nef secreted in EVs is rapidly taken up by neural cells, causes an increase in total tau, and redistribution of tau to lipid rafts in plasma membranes of SH-SY5Y cells [259]. This accumulation of tau within plasma membrane could be disrupted with methyl-β-cyclodextrin, a non-specific cholesterol acceptor that abrogates the formation of lipid rafts.

The association between HTLV-1 and tauopathy is less established and more controversial. Sousa et al*.* have demonstrated that total tau in the CSF did not differ among control, HTLV-1-infected and HTLV-1-associated myelopathy/tropical spastic paraparesis (HAM/TSP) patients [260]. Conversely, Maldonado et al*.* reported decreased total tau in the CSF of HAM/TSP patients, with concomitant increase of p181tau, with no changes in other tau phosphorylation sites after using a panel of ptau reactive antibodies [261]. Consistently, SH-SY5Y neurons treated with culture supernatant from HTLV-1-infected MT-2 cells (which secrete the viral protein Tax) showed an increase in p181Tau, along with CDK5 activation and neurite retraction [261, 262].

##### Endogenous retroviruses (ERVs)

Enhanced expression of ERVs has been repeatedly described to be associated with several tauopathies, including AD, ALS, and FTD [212, 263–266]. Upregulation of ERVs promotes the dissemination of protein aggregates between cells, including tau [267]. Similar to the HSV-1 context, the pathogenic protein aggregate spread appears to be through the capture and dissemination of these protein condensates by EVs and viral particles [267]. In a murine Moloney leukemia virus model, retroviral envelope proteins coating these vesicles facilitate EV spread and antibodies targeting the envelope proteins can inhibit intercellular transmission of protein aggregates [267]. However, the tau proteopathic seed spreading was most effective when donor cells produced active viral particles and expressed all core retroviral gene products [267]. Further experiments showed that in HEK cell cocultures, donor cell expression of ERVW Env Syncytin-1 or ERVK Env can facilitate intercellular spreading of tau aggregates, which was dependent on the association of the viral envelope proteins with their respective cellular receptors [267].

**Fig. 3:**
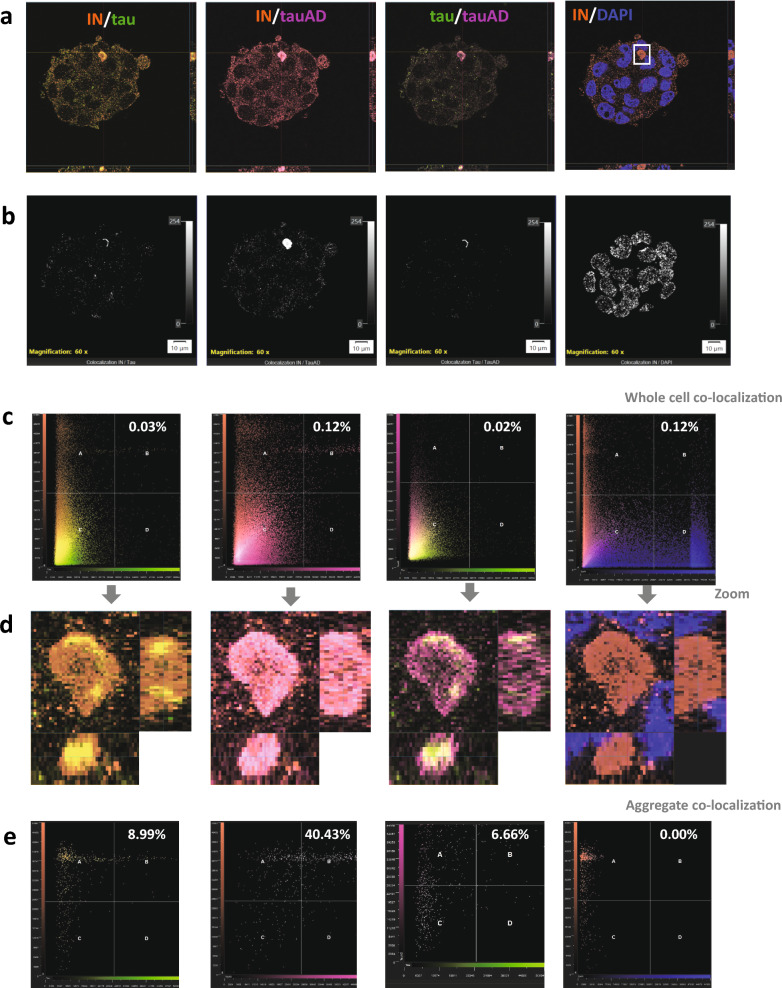
Endogenous retrovirus-K (ERVK) integrase forms cellular protein deposits with Alzheimer’s disease (AD)-associated misfolded tau. ERVK proteins are produced by teratocarcinoma NCCIT cells. **a** Representative images showing colocalization of ERVK integrase enzyme (IN), total tau, and misfolded tau in the cytoplasmic inclusion body (highlighted by a square box) of NCCIT cells. Nuclei are stained with DAPI. **b** Scatter plot analysis of fluorescence intensity depicting colocalization distribution across whole NCCIT cells. **c** ERVK IN predominantly co-localizes with AD-tau and native tau in cytoplasmic foci compared to the entire cell. **d** Magnified slice view of the cytoplasmic foci displaying colocalization of ERVK IN with tau isoforms. **e** Scatter plot analysis reveals a higher colocalization intensity of ERVK IN with misfolded AD-tau (40.43%) in cytoplasmic foci compared to ERVK IN with total tau (8.99%)

In ALS, ERVK viral protein aggregation is an observed but unexpected aspect of TDP-43 misregulation [268]. Recent data from our lab indicate that human ERVK viral proteins also form cellular condensates with both native tau and misfolded AD-associated tau (Fig. 3). The misfolded tau was co-localized with ERVK integrase protein in droplets in ERVK virion-producing NCCIT teratocarcinoma cells. Native tau was largely distributed throughout the cytoplasm, with some accumulation in the shell of the droplets. A strong accumulation of AD-associated tau could be observed in the core of the aggregates. This provides further evidence that both exogenous and endogenous viruses can modulate tau deposition and aggregation (as well as putatively other misfolded and aggregate-prone proteins like TDP-43 and FUS [269]), suggesting a possible involvement in various neurodegenerative conditions. Moreover, ERVK Env protein in the plasma-derived EVs may serve as a biomarker for ALS [270]. Together, growing evidence suggests that therapeutic intervention of TE activity may be part of an effective strategy to prevent neurodegenerative proteinopathies, specifically tauopathy [157, 212, 271]. Yet, to identify effective anti-TE therapeutics, careful stratification of patients based on TE expression (increased or not) will be necessary to address disease heterogeneity in clinical trials for tauopathies and other neurodegenerative conditions [272].

#### Molecular mechanisms underpinning viral modulation of tau-associated pathways

##### Potential viral modulation of tau isoforms

Tau-related intron retention is a developing focus in AD pathology [273]. Intron retention is regulated by various factors, specifically certain RBPs such as PABPC1 (discussed above). Intron retention can result in a truncated tau isoform (Tau11i), which forms the hallmark insoluble tau aggregates of AD pathology [128]. Tau isoforms formed due to retention of introns 3, 11, and 12 are upregulated in AD brains, providing insights into the mechanism of tauopathy [273]. At the intersection of these processes is the potential impact of virus-mediated proteolysis of PABPC1, which has been widely described for several retroviruses, and PABPC1 relocalization, in the case of herpesviruses and rotaviruses (reviewed in [125, 274]). For example, HIV-1 protease can cleave PABPC1 into fragments, leading to the removal of the first two RRMs from the C-terminal domain [275]. With substantial potential to alter cellular mRNA translation and NMD, this viral evasion strategy is necessary to maintain viral replication, as PABPC1 acts as an inhibitory factor binding to the HIV-1 *cis-*acting repressive sequences in viral mRNA, thus preventing viral protein expression [276]. Conversely, the interactions between ORF2 and PABPC1 are required by LINE-1 retrotransposons to interact with their viral mRNA during the process of retrotransposition [277]. Yet, what remains largely unknown is how modulation of PABPC1 by specific viruses impacts MAPT isoforms and mRNA stability in tauopathies.

##### Viruses can impact LLPS and SG formation

**Fig. 4:**
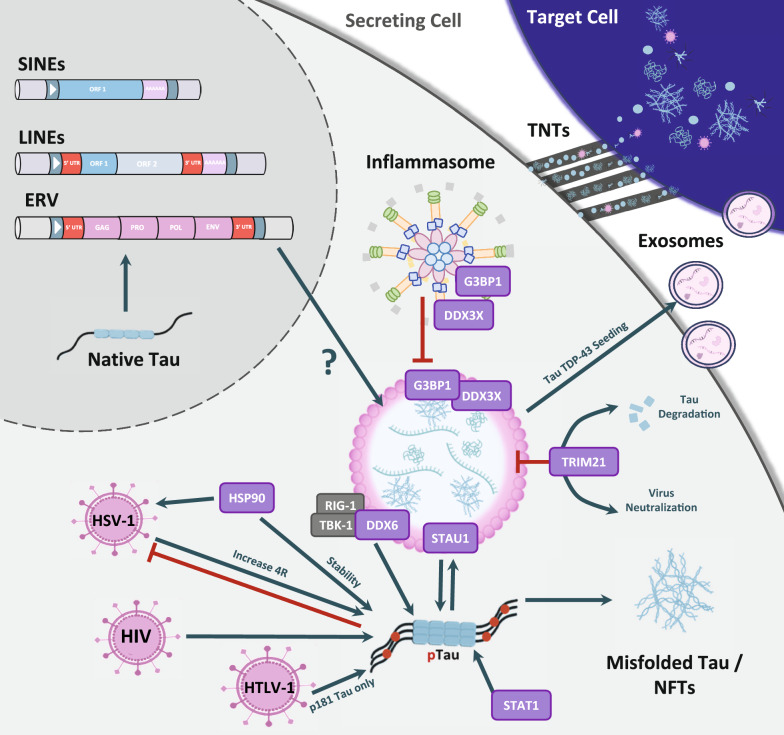
Stress granules as a hub for tau and virus interactions. The schematic depicts the molecular interactions and processes by which viruses and endogenous retroelements contribute to the dysregulation, aggregation, and intracellular propagation of tau. Stress granule (SG)-associated proteins (purple) that are predicted to influence both tau and viruses are shown in key pathways. In the secreting cell, native tau affects the expression of endogenous retroviruses (ERVs), long interspersed nuclear elements (LINEs), and short interspersed nuclear elements (SINEs) with ERVs possibly affecting SG dynamics. SGs serve as the center element where STAU1 and DDX6 interact directly with tau. Inflammasomes inhibit SG formation by sequestering DDX3X and G3BP1. Viral infections by HSV-1, HIV, and HTLV-1 further influence tau pathology by modifying the stability, splicing and pathways of degradation of tau. TRIM-21 is associated with tau degradation and neutralization of viruses. Conversely, HSP90 plays a role in the stabilization of tau and HSV-1 replication. STAT1 activity leads to tau phosphorylation. Aggregated or misfolded tau leads to the formation of neurofibrillary tangles (NFTs) and has the potential to initiate TDP-43 pathology. Viruses and pathogenic tau can be transferred to the target cell through exosomes or tunneling nanotubes (TNTs), contributing to the spread of disease pathology

Viruses can dramatically change how the host cell functions and the cellular architecture [278]. As described above, many exogenous viruses associated with tauopathies are capable of regulating viral condensates within the cell [278]. SGs and LLPS are often part of an antiviral response [279–281]; therefore, there may be an association between SG formation due to virus infection and tau aggregation (Fig. 4) [282].

Upon viral infection, SGs form and exert antiviral effects by halting the translation of many cellular proteins required for viral replication [282]. Viruses can counter-regulate the formation of SGs or form modified SGs, which are also sometimes referred to as inclusion bodies, viral replication compartments, viral factories, virosomes, or viroplasms [278]. These biomolecular condensates are composed of viral proteins, host cell proteins, and nucleic acids of both viral and cellular origins [278]. Notably, SGs that form in response to viral infection, as compared to those in response to heat shock or sodium arsenite, behave differently due to the strategies viruses use to modify SGs (reviewed in [278, 282]). For example, the SARS-CoV2 nucleocapsid (N) protein is predicted to interact with SG proteins [283, 284] and can precipitate LLPS when combined with a non-specific homopolymeric RNA [285]. Within these condensates, the N protein surrounds and associates with other cellular hnRNPs, such as TDP-43, FUS and hnRNPA2[285]. The RBPs may serve as a scaffold to facilitate the formation of N protein-RNA condensates, as a means to enable or accelerate viral replication [285]. Thus, RNA-containing multiprotein complexes like SGs may serve as an assembly machinery for viruses (with modifications for that purpose), favouring viral replication over host needs [286]. This may result in viral capture of tau, and the subsequent tau PTM or structural modification within the condensates. Nonetheless, interactions between SGs, LLPS, and viruses are complex and require investigations on a case-by-case basis, as tau-related mechanisms may be context- and disease-specific.

## Conclusions

It is becoming clear that viruses can interface with tau biology and potentially contribute to a transition towards tauopathy. This association suggests viruses as a potential target in ameliorating tau pathology and opens up a strategic toolkit of antiviral therapeutics for neurodegenerative disease. Although viral proteins and nucleic acids are not traditionally considered as integral components of SGs in tauopathies, there is ample evidence to explore this possibility in terms of rational therapeutic designs and as add-on biomarkers for clinical trials. Having highlighted the overlapping SG components between tau and virus-associated networks, it becomes clear that selective proteins (including G3BP1, DDX family proteins, STAU1, and PABPC1) may form intersecting hubs linking viral activity to tauopathy. Moreover, we provide a concrete example of a tauopathy-inducing virus, ERVK, whose activity is tied to viral protein deposits which phase separate with AD-associated misfolded tau in cytoplasmic inclusions. Together, accumulating evidence points to a need to further explore how viruses contribute to the development of tauopathies. Targeting viral activity may modulate the clinical outcomes of these neurodegenerative conditions.

## Supplementary Information

Additional file 1. **Table S1**. Stress granule-associated genes and their association with tau or viral pathways. **Table S2**. Viral pathways associated with the stress granule proteome. **Table S3**. Categorization of Mammalian Stress Granule Proteins. **Methodology**.

## Data Availability

The Mammalian Stress Granules Proteome (MSGP) database (https://msgp.pt/) and the BioGRID database (https://thebiogrid.org/) can be used to access proteins listed in the paper.

## Abbreviations

AD: Alzheimer’s disease
ALS: Amyotrophic Lateral Sclerosis
CSF: Cerebrospinal fluid
DDR: DNA damage response
DDX: DEAD-box
DSB: Double-stranded break
ERVs: Endogenous retroviruses
FTD: Frontotemporal dementia
HIV: Human immunodeficiency virus
HSPs: Heat shock proteins
HSV-1: Herpes simplex virus 1
HTLV: Human T-lymphotropic virus
LLPS: Liquid–liquid phase separation
MAPT: Microtubule-associated protein tau
MTBD: Microtubule-binding domain
NFT: Neurofibrillary tangles
PiD: Pick’s disease
PRD: Proline-rich domain
PTMs: Post-translational modifications
RBPs: RNA-binding proteins
RLR: RIG-I-like receptor
SG: Stress granule
TEs: Transposable elements
TNTs: Tunneling nanotubes
