# Supplementary material for: Implications of virus-induced stress granules in tauopathies
Source: Transl Neurodegener. 2026 Feb 12;15:4. doi: 10.1186/s40035-026-00538-4 (PMC12896018; doi:10.1186/s40035-026-00538-4)
Supplement: Supplementary file 1 — Additional file 1. Table S1. Stress granule-associated genes and their association with tau or viral pathways. Table S2. Viral pathways associated with the stress granule proteome. Table S3. Categorization of Mammalian Stress Granule Proteins. Methodology. [file 40035_2026_538_MOESM1_ESM.pdf]

Supplementary materials

Table S1: Stress granule-associated genes and their association with tau or viral pathways.

Uniprot ID	Entry name	Gene names	Protein names	Tau	Virus
Q562R1	ACTBL_HUMAN	ACTBL2	Beta-actin-like protein 2 (Kappa-actin)	●	
P61163	ACTZ_HUMAN	ACTR1A CTRN1	Alpha-centractin (Centractin) (ARP1) (Actin-RPV) (Centrosome-associated actin homolog)	●	
P42025	ACTY_HUMAN	ACTR1B CTRN2	Beta-centractin (Actin-related protein 1B) (ARP1B)	●	
P55265	DSRAD_HUMAN	ADAR ADAR1 DSRAD G1P1 IFI4	Double-stranded RNA-specific adenosine deaminase (DRADA) (EC 3.5.4.37) (136 kDa double-stranded RNA-binding protein) (p136) (Interferon-inducible protein 4) (IFI-4) (K88DSRBP)		●
Q9UL18	AGO1_HUMAN	AGO1 EIF2C1	Protein argonaute-1 (Argonaute1) (hAgo1) (Argonaute RISC catalytic component 1) (Eukaryotic translation initiation factor 2C 1) (eIF-2C 1) (eIF2C 1) (Putative RNA-binding protein Q99)	●	
Q9UKV8	AGO2_HUMAN	AGO2 EIF2C2	Protein argonaute-2 (Argonaute2) (hAgo2) (EC 3.1.26.n2) (Argonaute RISC catalytic component 2) (Eukaryotic translation initiation factor 2C 2) (eIF-2C 2) (eIF2C 2) (PAZ Piwi domain protein) (PPD) (Protein slicer)		
Q99996	AKAP9_HUMAN	AKAP9 AKAP350 AKAP450 KIAA0803	A-kinase anchor protein 9 (AKAP-9) (A-kinase anchor protein 350 kDa) (AKAP 350) (hgAKAP 350) (A-kinase anchor protein 450 kDa) (AKAP 450) (AKAP 120-like protein) (Centrosome- and Golgi-localized PKN-associated protein) (CG-NAP) (Protein hyperion) (Protein kinase A-anchoring protein 9) (PRKA9) (Protein yotiao)		
P54886	P5CS_HUMAN	ALDH18A1 GSAS P5CS PYCS	Delta-1-pyrroline-5-carboxylate synthase (P5CS) (Aldehyde dehydrogenase family 18 member A1) [Includes: Glutamate 5-kinase (GK) (EC 2.7.2.11) (Gamma-glutamyl kinase); Gamma-glutamyl phosphate reductase (GPR) (EC 1.2.1.41) (Glutamate-5-semialdehyde dehydrogenase) (Glutamyl-gamma-semialdehyde dehydrogenase)]		

P03950	ANGI_HUMAN	ANG RNASE5	Angiogenin (EC 3.1.27.-) (Ribonuclease 5) (RNase 5)		
Q9BTT0	AN32E_HUMAN	ANP32E	Acidic leucine-rich nuclear phosphoprotein 32 family member E (LANP-like protein) (LANP-L)		
P04083	ANXA1_HUMAN	ANXA1 ANX1 LPC1	Annexin A1 (Annexin I) (Annexin-1) (Calpactin II) (Calpactin-2) (Chromobindin-9) (Lipocortin I) (Phospholipase A2 inhibitory protein) (p35) [Cleaved into: Annexin Ac2-26]		
P08133	ANXA6_HUMAN	ANXA6 ANX6	Annexin A6 (67 kDa calelectrin) (Annexin VI) (Annexin-6) (Calphobindin-II) (CPB-II) (Chromobindin-20) (Lipocortin VI) (Protein III) (p68) (p70)	●	
P20073	ANXA7_HUMAN	ANXA7 ANX7 SNX OK/SW-cl.95	Annexin A7 (Annexin VII) (Annexin-7) (Synexin)		
P27695	APEX1_HUMAN	APEX1 APE APE1 APEX APX HAP1 REF1	DNA repair nuclease/redox regulator APEX1 (EC 3.1.11.2) (EC 3.1.21.-) (APEX nuclease) (APEN) (Apurinic-apyrimidinic endonuclease 1) (AP endonuclease 1) (APE-1) (DNA-(apurinic or apyrimidinic site) endonuclease) (Redox factor-1) (REF-1) [Cleaved into: DNA repair nuclease/redox regulator APEX1, mitochondrial]	●	
Q9HC16	ABC3G_HUMAN	APOBEC3G MDS019	DNA dC->dU-editing enzyme APOBEC-3G (EC 3.5.4.38) (APOBEC-related cytidine deaminase) (APOBEC-related protein) (ARCD) (APOBEC-related protein 9) (ARP-9) (CEM-15) (CEM15) (Deoxycytidine deaminase) (A3G)		●
O15143	ARC1B_HUMAN	ARPC1B ARC41	Actin-related protein 2/3 complex subunit 1B (Arp2/3 complex 41 kDa subunit) (p41-ARC)		
Q6PL18	ATAD2_HUMAN	ATAD2 L16 PRO2000	ATPase family AAA domain-containing protein 2 (EC 3.6.1.-) (AAA nuclear coregulator cancer-associated protein) (ANCCA)		
Q9NVI7	ATD3A_HUMAN	ATAD3A	ATPase family AAA domain-containing protein 3A (EC 3.6.1.-)		●
P98194	AT2C1_HUMAN	ATP2C1 KIAA1347 PMR1L HUSSY-28	Calcium-transporting ATPase type 2C member 1 (ATPase 2C1) (EC 7.2.2.10) (ATP-dependent Ca(2+) pump PMR1) (Ca(2+)/Mn(2+)-ATPase 2C1) (Secretory pathway Ca(2+)-transporting ATPase type 1) (SPCA1)		
P25705	ATPA_HUMAN	ATP5F1A ATP5A ATP5A1	ATP synthase subunit alpha, mitochondrial (ATP synthase F1 subunit alpha)		

		ATP5AL2 ATPM			
Q99700	ATX2_HUMAN	ATXN2 ATX2 SCA2 TNRC13	Ataxin-2 (Spinocerebellar ataxia type 2 protein) (Trinucleotide repeat-containing gene 13 protein)	●	
Q8WWM7	ATX2L_HUMAN	ATXN2L A2D A2LG A2LP A2RP	Ataxin-2-like protein (Ataxin-2 domain protein) (Ataxin-2-related protein)		
O95817	BAG3_HUMAN	BAG3 BIS	BAG family molecular chaperone regulator 3 (BAG-3) (Bcl-2-associated athanogene 3) (Bcl-2-binding protein Bis) (Docking protein CAIR-1)	●	
O75531	BAF_HUMAN	BANF1 BAF BCRG1	Barrier-to-autointegration factor (Breakpoint cluster region protein 1) [Cleaved into: Barrier-to-autointegration factor, N-terminally processed]		●
Q6PJG6	BRAT1_HUMAN	BRAT1 BAAT1 C7orf27	BRCA1-associated ATM activator 1 (BRCA1-associated protein required for ATM activation protein 1)		
Q92994	TF3B_HUMAN	BRF1 BRF GTF3B TAF3B2 TAF3C	Transcription factor IIIB 90 kDa subunit (TFIIIB90) (hTFIIIB90) (B-related factor 1) (BRF-1) (hBRF) (TAF3B2) (TATA box-binding protein-associated factor, RNA polymerase III, subunit 2)		
Q9HAW0	BRF2_HUMAN	BRF2 BRFU PRO1470	Transcription factor IIIB 50 kDa subunit (TFIIIB50) (hTFIIIB50) (B-related factor 2) (BRF-2) (hBRFU)		
Q96LT7	CI072_HUMAN	C9orf72 DENND9 DENNL72	Guanine nucleotide exchange factor C9orf72		
Q9NZT1	CALL5_HUMAN	CALML5 CLSP	Calmodulin-like protein 5 (Calmodulin-like skin protein)		
Q96L12	CALR3_HUMAN	CALR3 CRT2	Calreticulin-3 (Calreticulin-2) (Calsperin)		
Q01518	CAP1_HUMAN	CAP1 CAP	Adenylyl cyclase-associated protein 1 (CAP 1)	●	
Q14444	CAPR1_HUMAN	CAPRIN1 GPIAP1 GPIP137 M11S1 RNG105	Caprin-1 (Cell cycle-associated protein 1) (Cytoplasmic activation- and proliferation-associated protein 1) (GPI-anchored membrane protein 1) (GPI-anchored protein p137) (GPI-p137) (p137GPI) (Membrane component chromosome 11 surface marker 1) (RNA granule protein 105)	●	
P47755	CAZA2_HUMAN	CAPZA2	F-actin-capping protein subunit alpha-2 (CapZ alpha-2)		
Q9Y2V2	CHSP1_HUMAN	CARHSP1	Calcium-regulated heat-stable protein 1 (Calcium-regulated heat-stable protein of 24 kDa) (CRHSP-24)	●	

O15234	CASC3_HUMAN	CASC3 MLN51	Protein CASC3 (Cancer susceptibility candidate gene 3 protein) (Metastatic lymph node gene 51 protein) (MLN 51) (Protein barentsz) (Btz)		
Q13951	PEBB_HUMAN	CBFB	Core-binding factor subunit beta (CBF-beta) (Polyomavirus enhancer-binding protein 2 beta subunit) (PEA2-beta) (PEBP2-beta) (SL3-3 enhancer factor 1 subunit beta) (SL3/AKV core-binding factor beta subunit)		
P83916	CBX1_HUMAN	CBX1 CBX	Chromobox protein homolog 1 (HP1Hsbeta) (Heterochromatin protein 1 homolog beta) (HP1 beta) (Heterochromatin protein p25) (M31) (Modifier 1 protein) (p25beta)		
Q8IX12	CCAR1_HUMAN	CCAR1 CARP1 DIS	Cell division cycle and apoptosis regulator protein 1 (Cell cycle and apoptosis regulatory protein 1) (CARP-1) (Death inducer with SAP domain)		
Q6ZUT6	CCD9B_HUMAN	CCDC9B C15orf52	Coiled-coil domain-containing protein 9B		
B4DUR8	B4DUR8_HUMAN	CCT3	T-complex protein 1 subunit gamma	●	
P40227	TCPZ_HUMAN	CCT6A CCT6 CCTZ	T-complex protein 1 subunit zeta (TCP-1-zeta) (Acute morphine dependence-related protein 2) (CCT-zeta-1) (Chaperonin containing T-complex polypeptide 1 subunit 6A) (HTR3) (Tcp20)		
P25063	CD24_HUMAN	CD24 CD24A	Signal transducer CD24 (Small cell lung carcinoma cluster 4 antigen) (CD antigen CD24)		
Q99459	CDC5L_HUMAN	CDC5L KIAA0432 PCDC5RP	Cell division cycle 5-like protein (Cdc5-like protein) (Pombe cdc5-related protein)		
Q6P1J9	CDC73_HUMAN	CDC73 C1orf28 HRPT2	Parafibromin (Cell division cycle protein 73 homolog) (Hyperparathyroidism 2 protein)		
P06493	CDK1_HUMAN	CDK1 CDC2 CDC28A CDKN1 P34CDC2	Cyclin-dependent kinase 1 (CDK1) (EC 2.7.11.22) (EC 2.7.11.23) (Cell division control protein 2 homolog) (Cell division protein kinase 1) (p34 protein kinase)	●	●
P24941	CDK2_HUMAN	CDK2 CDKN2	Cyclin-dependent kinase 2 (EC 2.7.11.22) (Cell division protein kinase 2) (p33 protein kinase)	●	
Q92879	CELF1_HUMAN	CELF1 BRUNOL2 CUGBP CUGBP1 NAB50	CUGBP Elav-like family member 1 (CELF-1) (50 kDa nuclear polyadenylated RNA-binding protein) (Bruno-like protein 2) (CUG triplet repeat RNA-binding protein 1) (CUG-BP1) (CUG-BP- and ETR-3-like factor 1) (Deadenylation factor CUG-BP)		

			(Embryo deadenylation element-binding protein homolog) (EDEN-BP homolog) (RNA-binding protein BRUNOL-2)		
Q49MI3	CERKL_HUMAN	CERKL	Ceramide kinase-like protein		
P23528	COF1_HUMAN	CFL1 CFL	Cofilin-1 (18 kDa phosphoprotein) (p18) (Cofilin, non-muscle isoform)	●	
Q9NX63	MIC19_HUMAN	CHCHD3 MIC19 MINOS3	MICOS complex subunit MIC19 (Coiled-coil-helix-coiled-coil-helix domain-containing protein 3)		
Q9UHD1	CHRD1_HUMAN	CHORDC1 CHP1	Cysteine and histidine-rich domain-containing protein 1 (CHORD domain-containing protein 1) (CHORD-containing protein 1) (CHP-1) (Protein morgana)		
Q99653	CHP1_HUMAN	CHP1 CHP	Calcineurin B homologous protein 1 (Calcineurin B-like protein) (Calcium-binding protein CHP) (Calcium-binding protein p22) (EF-hand calcium-binding domain-containing protein p22)		
Q14011	CIRBP_HUMAN	CIRBP A18HNRNP CIRP	Cold-inducible RNA-binding protein (A18 hnRNP) (Glycine-rich RNA-binding protein CIRP)	●	
O14578	CTRO_HUMAN	CIT CRIK KIAA0949 STK21	Citron Rho-interacting kinase (CRIK) (EC 2.7.11.1) (Serine/threonine-protein kinase 21)		
Q9Y696	CLIC4_HUMAN	CLIC4	Chloride intracellular channel protein 4 (Glutaredoxin-like oxidoreductase CLIC4) (EC 1.8.-.-) (Intracellular chloride ion channel protein p64H1)	●	
P62633	CNBP_HUMAN	CNBP RNF163 ZNF9	CCHC-type zinc finger nucleic acid binding protein (Cellular nucleic acid-binding protein) (CNBP) (Zinc finger protein 9)		
Q15417	CNN3_HUMAN	CNN3	Calponin-3 (Calponin, acidic isoform)	●	
A5YKK6	CNOT1_HUMAN	CNOT1 CDC39 KIAA1007 NOT1 AD-005	CCR4-NOT transcription complex subunit 1 (CCR4-associated factor 1) (Negative regulator of transcription subunit 1 homolog) (NOT1H) (hNOT1)		
Q9BR76	COR1B_HUMAN	CORO1B	Coronin-1B (Coronin-2)		
Q9BZB8	CPEB1_HUMAN	CPEB1 CPEB	Cytoplasmic polyadenylation element-binding protein 1 (CPE-BP1) (CPE-binding protein 1) (h-CPEB) (hCPEB-1)		
Q9UKF6	CPSF3_HUMAN	CPSF3 CPSF73	Cleavage and polyadenylation specificity factor subunit 3 (EC 3.1.27.-) (Cleavage and polyadenylation specificity factor 73 kDa subunit) (CPSF 73 kDa		

			subunit) (mRNA 3'-end-processing endonuclease CPSF-73)		
Q16630	CPSF6_HUMAN	CPSF6 CFIM68	Cleavage and polyadenylation specificity factor subunit 6 (Cleavage and polyadenylation specificity factor 68 kDa subunit) (CPSF 68 kDa subunit) (Cleavage factor Im complex 68 kDa subunit) (CFIm68) (Pre-mRNA cleavage factor Im 68 kDa subunit) (Protein HPBRII-4/7)		
Q8N684	CPSF7_HUMAN	CPSF7	Cleavage and polyadenylation specificity factor subunit 7 (Cleavage and polyadenylation specificity factor 59 kDa subunit) (CPSF 59 kDa subunit) (Cleavage factor Im complex 59 kDa subunit) (CFIm59) (Pre-mRNA cleavage factor Im 59 kDa subunit)	●	
P02511	CRYAB_HUMAN	CRYAB CRYA2 HSPB5	Alpha-crystallin B chain (Alpha(B)-crystallin) (Heat shock protein beta-5) (HspB5) (Heat shock protein family B member 5) (Renal carcinoma antigen NY-REN-27) (Rosenthal fiber component)		
O75534	CSDE1_HUMAN	CSDE1 D1S155E KIAA0885 NRU UNR	Cold shock domain-containing protein E1 (N-ras upstream gene protein) (Protein UNR)		●
P55060	XPO2_HUMAN	CSE1L CAS XPO2	Exportin-2 (Exp2) (Cellular apoptosis susceptibility protein) (Chromosome segregation 1-like protein) (Importin-alpha re-exporter)		
Q05048	CSTF1_HUMAN	CSTF1	Cleavage stimulation factor subunit 1 (CF-1 50 kDa subunit) (Cleavage stimulation factor 50 kDa subunit) (CSTF 50 kDa subunit) (CstF-50)		
P26232	CTNA2_HUMAN	CTNNA2 CAPR	Catenin alpha-2 (Alpha N-catenin) (Alpha-catenin-related protein)	●	
O60716	CTND1_HUMAN	CTNND1 KIAA0384	Catenin delta-1 (Cadherin-associated Src substrate) (CAS) (p120 catenin) (p120(ctn)) (p120(cas))	●	
Q9P2B4	CT2NL_HUMAN	CTTNBP2NL KIAA1433	CTTNBP2 N-terminal-like protein		
Q9HCG8	CWC22_HUMAN	CWC22 KIAA1604 NCM	Pre-mRNA-splicing factor CWC22 homolog (Nucampholin homolog) (fSAPb)		
Q96EP5	DAZP1_HUMAN	DAZAP1	DAZ-associated protein 1 (Deleted in azoospermia-associated protein 1)		

Q15038	DAZP2_HUMAN	DAZAP2 KIAA0058 PRTB	DAZ-associated protein 2 (Deleted in azoospermia-associated protein 2) (Proline-rich transcript in brain protein)		
Q92904	DAZL_HUMAN	DAZL DAZH DAZL1 DAZLA SPGYLA	Deleted in azoospermia-like (DAZ homolog) (DAZ-like autosomal) (Deleted in azoospermia-like 1) (SPGY-like-autosomal)		
P81605	DCD_HUMAN	DCD AIDD DSEP	Dermcidin (EC 3.4.-.-) (Preproteolysin) [Cleaved into: Survival-promoting peptide; DCD-1]		
Q9NPI6	DCP1A_HUMAN	DCP1A SMIF	mRNA-decapping enzyme 1A (EC 3.6.1.62) (Smad4-interacting transcriptional co-activator) (Transcription factor SMIF)		
Q14203	DCTN1_HUMAN	DCTN1	Dynactin subunit 1 (150 kDa dynein-associated polypeptide) (DAP-150) (DP-150) (p135) (p150-glued)	●	
Q92499	DDX1_HUMAN	DDX1	ATP-dependent RNA helicase DDX1 (EC 3.6.4.13) (DEAD box protein 1) (DEAD box protein retinoblastoma) (DBP-RB)	●	●
Q9NUU7	DD19A_HUMAN	DDX19A DDX19L	ATP-dependent RNA helicase DDX19A (EC 3.6.4.13) (DDX19-like protein) (DEAD box protein 19A)		
Q9NR30	DDX21_HUMAN	DDX21	Nucleolar RNA helicase 2 (EC 3.6.4.13) (DEAD box protein 21) (Gu-alpha) (Nucleolar RNA helicase Gu) (Nucleolar RNA helicase II) (RH II/Gu)	●	●
O00571	DDX3X_HUMAN	DDX3X DBX DDX3	ATP-dependent RNA helicase DDX3X (EC 3.6.4.13) (CAP-Rf) (DEAD box protein 3, X-chromosomal) (DEAD box, X isoform) (DBX) (Helicase-like protein 2) (HLP2)	●	●
Q9H0S4	DDX47_HUMAN	DDX47	Probable ATP-dependent RNA helicase DDX47 (EC 3.6.4.13) (DEAD box protein 47)		
Q9BQ39	DDX50_HUMAN	DDX50	ATP-dependent RNA helicase DDX50 (EC 3.6.4.13) (DEAD box protein 50) (Gu-beta) (Nucleolar protein Gu2)		
O95786	RIGI_HUMAN	RIGI DDX58	Antiviral innate immune response receptor RIG-I (ATP-dependent RNA helicase DDX58) (EC 3.6.4.13) (DEAD box protein 58) (RIG-I-like receptor 1) (RLR-1) (RNA sensor RIG-I) (Retinoic acid-inducible gene 1 protein) (RIG-1) (Retinoic acid-inducible gene I protein) (RIG-I)		●
P26196	DDX6_HUMAN	DDX6 HLR2 RCK	Probable ATP-dependent RNA helicase DDX6 (EC 3.6.4.13) (ATP-dependent RNA helicase p54) (DEAD box protein 6) (Oncogene RCK)	●	●

Q9Y315	DEOC_HUMAN	DERA CGI-26	Deoxyribose-phosphate aldolase (DERA) (EC 4.1.2.4) (2-deoxy-D-ribose 5-phosphate aldolase) (Phosphodeoxyriboaldolase) (Deoxyriboaldolase)		
Q7L2E3	DHX30_HUMAN	DHX30 DDX30 KIAA0890	ATP-dependent RNA helicase DHX30 (EC 3.6.4.13) (DEAH box protein 30)		
Q9H2U1	DHX36_HUMAN	DHX36 DDX36 KIAA1488 MLEL1 RHAU	ATP-dependent DNA/RNA helicase DHX36 (EC 3.6.4.12) (EC 3.6.4.13) (DEAD/H box polypeptide 36) (DEAH-box protein 36) (G4-resolvase-1) (G4R1) (MLE-like protein 1) (RNA helicase associated with AU-rich element protein)		●
Q9NRI5	DISC1_HUMAN	DISC1 KIAA0457	Disrupted in schizophrenia 1 protein		
O60832	DKC1_HUMAN	DKC1 NOLA4	H/ACA ribonucleoprotein complex subunit DKC1 (EC 5.4.99.-) (CBF5 homolog) (Dyskerin) (Nopp140-associated protein of 57 kDa) (Nucleolar protein NAP57) (Nucleolar protein family A member 4) (snoRNP protein DKC1)		
P31689	DNJA1_HUMAN	DNAJA1 DNAJ2 HDJ2 HSJ2 HSPF4	DnaJ homolog subfamily A member 1 (DnaJ protein homolog 2) (HSDJ) (Heat shock 40 kDa protein 4) (Heat shock protein J2) (HSJ-2) (Human DnaJ protein 2) (hDj-2)	●	
O75937	DNJC8_HUMAN	DNAJC8 SPF31 HSPC315 HSPC331	DnaJ homolog subfamily C member 8 (Splicing protein spf31)	●	
Q13409	DC1I2_HUMAN	DYNC1I2 DNCI2 DNCIC2	Cytoplasmic dynein 1 intermediate chain 2 (Cytoplasmic dynein intermediate chain 2) (Dynein intermediate chain 2, cytosolic) (DH IC-2)		
Q16555	DPYL2_HUMAN	DPYSL2 CRMP2 ULIP2	Dihydropyrimidinase-related protein 2 (DRP-2) (Collapsin response mediator protein 2) (CRMP-2) (N2A3) (Unc-33-like phosphoprotein 2) (ULIP-2)	●	
Q14195	DPYL3_HUMAN	DPYSL3 CRMP4 DRP3 ULIP ULIP1	Dihydropyrimidinase-related protein 3 (DRP-3) (Collapsin response mediator protein 4) (CRMP-4) (Unc-33-like phosphoprotein 1) (ULIP-1)	●	
P15924	DESP_HUMAN	DSP	Desmoplakin (DP) (250/210 kDa paraneoplastic pemphigus antigen)		
Q03001	DYST_HUMAN	DST BP230 BP240 BPAG1 DMH DT KIAA0728	Dystonin (230 kDa bullous pemphigoid antigen) (230/240 kDa bullous pemphigoid antigen) (Bullous pemphigoid antigen 1) (BPA) (Bullous pemphigoid antigen) (Dystonia musculorum protein) (Hemidesmosomal plaque protein)		

P60981	DEST_HUMAN	DSTN ACTDP DSN	Destrin (Actin-depolymerizing factor) (ADF)	●	
Q8TDB6	DTX3L_HUMAN	DTX3L BBAP	E3 ubiquitin-protein ligase DTX3L (EC 2.3.2.27) (B-lymphoma- and BAL-associated protein) (Protein deltex-3-like) (RING-type E3 ubiquitin transferase DTX3L) (Rhysin-2) (Rhysin2)		●
Q14204	DYHC1_HUMAN	DYNC1H1 DHC1 DNCH1 DNCL DNECL DYHC KIAA0325	Cytoplasmic dynein 1 heavy chain 1 (Cytoplasmic dynein heavy chain 1) (Dynein heavy chain, cytosolic)	●	
Q96FJ2	DYL2_HUMAN	DYNLL2 DLC2	Dynein light chain 2, cytoplasmic (8 kDa dynein light chain b) (DLC8b) (Dynein light chain LC8-type 2)		
O43781	DYRK3_HUMAN	DYRK3	Dual specificity tyrosine-phosphorylation-regulated kinase 3 (EC 2.7.12.1) (Regulatory erythroid kinase) (REDK)		
Q86YF9	DZIP1_HUMAN	DZIP1 DZIP DZIP2 KIAA0996	Cilium assembly protein DZIP1 (DAZ-interacting protein 1/2) (DAZ-interacting zinc finger protein 1)		
Q6P2E9	EDC4_HUMAN	EDC4 HEDLS	Enhancer of mRNA-decapping protein 4 (Autoantigen Ge-1) (Autoantigen RCD-8) (Human enhancer of decapping large subunit) (Hedls)		
Q9BY44	EIF2A_HUMAN	EIF2A CDA02 MSTP004 MSTP089	Eukaryotic translation initiation factor 2A (eIF-2A) (65 kDa eukaryotic translation initiation factor 2A) [Cleaved into: Eukaryotic translation initiation factor 2A, N-terminally processed]	●	
Q9BQI3	E2AK1_HUMAN	EIF2AK1 HRI KIAA1369 PRO1362	Eukaryotic translation initiation factor 2-alpha kinase 1 (EC 2.7.11.1) (Heme-controlled repressor) (HCR) (Heme-regulated eukaryotic initiation factor eIF-2-alpha kinase) (Heme-regulated inhibitor) (hHRI) (Hemin-sensitive initiation factor 2-alpha kinase)		
P19525	E2AK2_HUMAN	EIF2AK2 PKR PRKR	Interferon-induced, double-stranded RNA-activated protein kinase (EC 2.7.11.1) (Eukaryotic translation initiation factor 2-alpha kinase 2) (eIF-2A protein kinase 2) (Interferon-inducible RNA-dependent protein kinase) (P1/eIF-2A protein kinase) (Protein kinase RNA-activated) (PKR) (Protein kinase R) (Tyrosine-protein kinase EIF2AK2) (EC 2.7.10.2) (p68 kinase)		●

P20042	IF2B_HUMAN	EIF2S2 EIF2B	Eukaryotic translation initiation factor 2 subunit 2 (Eukaryotic translation initiation factor 2 subunit beta) (eIF2-beta)	●	
Q14152	EIF3A_HUMAN	EIF3A EIF3S10 KIAA0139	Eukaryotic translation initiation factor 3 subunit A (eIF3a) (Eukaryotic translation initiation factor 3 subunit 10) (eIF-3-theta) (eIF3 p167) (eIF3 p180) (eIF3 p185)	●	●
P55884	EIF3B_HUMAN	EIF3B EIF3S9	Eukaryotic translation initiation factor 3 subunit B (eIF3b) (Eukaryotic translation initiation factor 3 subunit 9) (Prt1 homolog) (hPrt1) (eIF-3-eta) (eIF3 p110) (eIF3 p116)		●
O15371	EIF3D_HUMAN	EIF3D EIF3S7	Eukaryotic translation initiation factor 3 subunit D (eIF3d) (Eukaryotic translation initiation factor 3 subunit 7) (eIF-3-zeta) (eIF3 p66)		●
P60228	EIF3E_HUMAN	EIF3E EIF3S6 INT6	Eukaryotic translation initiation factor 3 subunit E (eIF3e) (Eukaryotic translation initiation factor 3 subunit 6) (Viral integration site protein INT-6 homolog) (eIF-3 p48)		
O00303	EIF3F_HUMAN	EIF3F EIF3S5	Eukaryotic translation initiation factor 3 subunit F (eIF3f) (Deubiquitinating enzyme eIF3f) (EC 3.4.19.12) (Eukaryotic translation initiation factor 3 subunit 5) (eIF-3-epsilon) (eIF3 p47)	●	●
O75821	EIF3G_HUMAN	EIF3G EIF3S4	Eukaryotic translation initiation factor 3 subunit G (eIF3g) (Eukaryotic translation initiation factor 3 RNA-binding subunit) (eIF-3 RNA-binding subunit) (Eukaryotic translation initiation factor 3 subunit 4) (eIF-3-delta) (eIF3 p42) (eIF3 p44)		●
O15372	EIF3H_HUMAN	EIF3H EIF3S3	Eukaryotic translation initiation factor 3 subunit H (eIF3h) (Eukaryotic translation initiation factor 3 subunit 3) (eIF-3-gamma) (eIF3 p40 subunit)		
Q13347	EIF3I_HUMAN	EIF3I EIF3S2 TRIP1	Eukaryotic translation initiation factor 3 subunit I (eIF3i) (Eukaryotic translation initiation factor 3 subunit 2) (TGF-beta receptor-interacting protein 1) (TRIP-1) (eIF-3-beta) (eIF3 p36)		
O75822	EIF3J_HUMAN	EIF3J EIF3S1 PRO0391	Eukaryotic translation initiation factor 3 subunit J (eIF3j) (Eukaryotic translation initiation factor 3 subunit 1) (eIF-3-alpha) (eIF3 p35)		
Q9UBQ5	EIF3K_HUMAN	EIF3K EIF3S12 ARG134 HSPC029	Eukaryotic translation initiation factor 3 subunit K (eIF3k) (Eukaryotic translation initiation factor 3 subunit 12) (Muscle-specific gene M9 protein) (PLAC-24) (eIF-3 p25) (eIF-3 p28)		

		MSTP001 PTD001			
Q9Y262	EIF3L_HUMAN	EIF3L EIF3EIP EIF3S6IP HSPC021 HSPC025 MSTP005	Eukaryotic translation initiation factor 3 subunit L (eIF3l) (Eukaryotic translation initiation factor 3 subunit 6-interacting protein) (Eukaryotic translation initiation factor 3 subunit E-interacting protein)		●
Q7L2H7	EIF3M_HUMAN	EIF3M HFLB5 PCID1 GA17 PNAS-125	Eukaryotic translation initiation factor 3 subunit M (eIF3m) (Fetal lung protein B5) (hFL-B5) (PCI domain-containing protein 1)		
P60842	IF4A1_HUMAN	EIF4A1 DDX2A EIF4A	Eukaryotic initiation factor 4A-I (eIF-4A-I) (eIF4A-I) (EC 3.6.4.13) (ATP-dependent RNA helicase eIF4A-1)	●	
P23588	IF4B_HUMAN	EIF4B	Eukaryotic translation initiation factor 4B (eIF-4B)	●	
P06730	IF4E_HUMAN	EIF4E EIF4EL1 EIF4F	Eukaryotic translation initiation factor 4E (eIF-4E) (eIF4E) (eIF-4F 25 kDa subunit) (mRNA cap-binding protein)		
Q04637	IF4G1_HUMAN	EIF4G1 EIF4F EIF4G EIF4GI	Eukaryotic translation initiation factor 4 gamma 1 (eIF-4-gamma 1) (eIF-4G 1) (eIF-4G1) (p220)		
P78344	IF4G2_HUMAN	EIF4G2 DAP5 OK/SW-cl.75	Eukaryotic translation initiation factor 4 gamma 2 (eIF-4-gamma 2) (eIF-4G 2) (eIF4G 2) (Death-associated protein 5) (DAP-5) (p97)	●	
Q15056	IF4H_HUMAN	EIF4H KIAA0038 WBSCR1 WSCR1	Eukaryotic translation initiation factor 4H (eIF-4H) (Williams-Beuren syndrome chromosomal region 1 protein)	●	
Q15717	ELAV1_HUMAN	ELAVL1 HUR	ELAV-like protein 1 (Hu-antigen R) (HuR)		
Q12926	ELAV2_HUMAN	ELAVL2 HUB	ELAV-like protein 2 (ELAV-like neuronal protein 1) (Hu-antigen B) (HuB) (Nervous system-specific RNA-binding protein Hel-N1)		
P26378	ELAV4_HUMAN	ELAVL4 HUD PNEM	ELAV-like protein 4 (Hu-antigen D) (HuD) (Paraneoplastic encephalomyelitis antigen HuD)	●	
P58107	EPIPL_HUMAN	EPPK1 EPIPL	Epiplakin (450 kDa epidermal antigen)		
P62495	ERF1_HUMAN	ETF1 ERF1 RF1 SUP45L1	Eukaryotic peptide chain release factor subunit 1 (Eukaryotic release factor 1) (eRF1) (Protein Cl1) (TB3-1)		
Q01844	EWS_HUMAN	EWSR1 EWS	RNA-binding protein EWS (EWS oncogene) (Ewing sarcoma breakpoint region 1 protein)	●	
Q05397	FAK1_HUMAN	PTK2 FAK FAK1	Focal adhesion kinase 1 (FADK 1) (EC 2.7.10.2) (Focal adhesion kinase-related nonkinase) (FRNK)		

			(Protein phosphatase 1 regulatory subunit 71) (PPP1R71) (Protein-tyrosine kinase 2) (p125FAK) (pp125FAK)		
Q9NZB2	F120A_HUMAN	FAM120A C9orf10 KIAA0183 OSSA	Constitutive coactivator of PPAR-gamma-like protein 1 (Oxidative stress-associated SRC activator) (Protein FAM120A)		
Q9BUT9	MCRI2_HUMAN	MCRIP2 C16orf14 FAM195A	MAPK regulated corepressor interacting protein 2 (Protein FAM195A)		
C9JLW8	MCRI1_HUMAN	MCRIP1 FAM195B GRAN2	Mapk-regulated corepressor-interacting protein 1 (Granulin-2) (Protein FAM195B)		
Q8NCA5	FA98A_HUMAN	FAM98A	Protein FAM98A		
Q14296	FASTK_HUMAN	FASTK	Fas-activated serine/threonine kinase (FAST kinase) (EC 2.7.11.1) (EC 2.7.11.8)		
P22087	FBRL_HUMAN	FBL FIB1 FLRN	rRNA 2'-O-methyltransferase fibrillarin (EC 2.1.1.-) (34 kDa nucleolar scleroderma antigen) (Histone-glutamine methyltransferase) (U6 snRNA 2'-O-methyltransferase fibrillarin)		
P09467	F16P1_HUMAN	FBP1 FBP	Fructose-1,6-bisphosphatase 1 (FBPase 1) (EC 3.1.3.11) (D-fructose-1,6-bisphosphate 1-phosphohydrolase 1) (Liver FBPase)		
O00757	F16P2_HUMAN	FBP2	Fructose-1,6-bisphosphatase isozyme 2 (FBPase 2) (EC 3.1.3.11) (D-fructose-1,6-bisphosphate 1-phosphohydrolase 2) (Muscle FBPase)		
Q13642	FHL1_HUMAN	FHL1 SLIM1	Four and a half LIM domains protein 1 (FHL-1) (Skeletal muscle LIM-protein 1) (SLIM) (SLIM-1)	●	
O75369	FLNB_HUMAN	FLNB FLN1L FLN3 TABP TAP	Filamin-B (FLN-B) (ABP-278) (ABP-280 homolog) (Actin-binding-like protein) (Beta-filamin) (Filamin homolog 1) (Fh1) (Filamin-3) (Thyroid autoantigen) (Truncated actin-binding protein) (Truncated ABP)		
Q06787	FMR1_HUMAN	FMR1	Fragile X messenger ribonucleoprotein 1 (Fragile X messenger ribonucleoprotein) (FMRP) (Protein FMR-1)		●
Q53EP0	FND3B_HUMAN	FNDC3B FAD104 NS5ABP37 UNQ2421/PRO 4979/PRO34274	Fibronectin type III domain-containing protein 3B (Factor for adipocyte differentiation 104) (HCV NS5A-binding protein 37)		

Q16658	FSCN1_HUMAN	FSCN1 FAN1 HSN SNL	Fascin (55 kDa actin-bundling protein) (Singed-like protein) (p55)	●	
Q8IY81	SPB1_HUMAN	FTSJ3 SB92	pre-rRNA 2'-O-ribose RNA methyltransferase FTSJ3 (EC 2.1.1.-) (Protein ftsJ homolog 3) (Putative rRNA methyltransferase 3)		
Q96I24	FUBP3_HUMAN	FUBP3 FBP3	Far upstream element-binding protein 3 (FUSE-binding protein 3)		
P35637	FUS_HUMAN	FUS TLS	RNA-binding protein FUS (75 kDa DNA-pairing protein) (Oncogene FUS) (Oncogene TLS) (POMp75) (Translocated in liposarcoma protein)	●	
P51114	FXR1_HUMAN	FXR1	RNA-binding protein FXR1 (FMR1 autosomal homolog 1) (hFXR1p)	●	
P51116	FXR2_HUMAN	FXR2 FMR1L2	RNA-binding protein FXR2 (FXR2P) (FMR1 autosomal homolog 2)		
Q13283	G3BP1_HUMAN	G3BP1 G3BP	Ras GTPase-activating protein-binding protein 1 (G3BP-1) (EC 3.6.4.12) (EC 3.6.4.13) (ATP-dependent DNA helicase VIII) (hDH VIII) (GAP SH3 domain-binding protein 1)	●	●
Q9UN86	G3BP2_HUMAN	G3BP2 KIAA0660	Ras GTPase-activating protein-binding protein 2 (G3BP-2) (GAP SH3 domain-binding protein 2)	●	
Q06210	GFPT1_HUMAN	GFPT1 GFAT GFPT	Glutamine--fructose-6-phosphate aminotransferase [isomerizing] 1 (EC 2.6.1.16) (D-fructose-6-phosphate amidotransferase 1) (Glutamine:fructose-6-phosphate amidotransferase 1) (GFAT 1) (GFAT1) (Hexosephosphate aminotransferase 1)	●	
Q53GS7	GLE1_HUMAN	GLE1 GLE1L	mRNA export factor GLE1 (hGLE1) (GLE1 RNA export mediator) (GLE1-like protein) (Nucleoporin GLE1)		
P62879	GBB2_HUMAN	GNB2	Guanine nucleotide-binding protein G(I)/G(S)/G(T) subunit beta-2 (G protein subunit beta-2) (Transducin beta chain 2)	●	
Q14451	GRB7_HUMAN	GRB7	Growth factor receptor-bound protein 7 (B47) (Epidermal growth factor receptor GRB-7) (GRB7 adapter protein)		
P15170	ERF3A_HUMAN	GSPT1 ERF3A	Eukaryotic peptide chain release factor GTP-binding subunit ERF3A (Eukaryotic peptide chain release factor subunit 3a) (eRF3a) (EC 3.6.5.-) (G1 to S phase transition protein 1 homolog)	●	
P07305	H10_HUMAN	H1-0 H1F0 H1FV	Histone H1.0 (Histone H1') (Histone H1(0)) [Cleaved into: Histone H1.0, N-terminally processed]		
Q92522	H1X_HUMAN	H1-10 H1FX	Histone H1.10 (Histone H1x)		

Q71UI9	H2AV_HUMAN	H2AZ2 H2AFV H2AV	Histone H2A.V (H2A.F/Z) (H2A.Z variant histone 2)		
Q5JVS0	HABP4_HUMAN	HABP4	Intracellular hyaluronan-binding protein 4 (IHABP-4) (IHABP4) (Hyaluronan-binding protein 4) (Ki-1/57 intracellular antigen)	●	
Q9UBN7	HDAC6_HUMAN	HDAC6 KIAA0901 JM21	Histone deacetylase 6 (HD6) (EC 3.5.1.98) (Protein deacetylase HDAC6) (EC 3.5.1.-) (Tubulin-lysine deacetylase HDAC6) (EC 3.5.1.-)	●	
P42694	HELZ_HUMAN	HELZ DRHC KIAA0054	Probable helicase with zinc finger domain (EC 3.6.4.-) (Down-regulated in human cancers protein)		
Q9BYK8	HELZ2_HUMAN	HELZ2 KIAA1769 PRIC285	3'-5' exoribonuclease HELZ2 (EC 3.1.13.1) (ATP-dependent RNA helicase PRIC285) (EC 3.6.4.13) (Helicase with zinc finger 2, transcriptional coactivator) (Helicase with zinc finger domain 2) (PPAR-alpha-interacting complex protein 285) (PPAR-gamma DNA-binding domain-interacting protein 1) (PDIP1) (PPAR-gamma DBD-interacting protein 1) (Peroxisomal proliferator-activated receptor A-interacting complex 285 kDa protein)		
P17096	HMGA1_HUMAN	HMGA1 HMGIY	High mobility group protein HMG-I/HMG-Y (HMG-I(Y)) (High mobility group AT-hook protein 1) (High mobility group protein A1) (High mobility group protein R)		
O15347	HMGB3_HUMAN	HMGB3 HMG2A HMG4	High mobility group protein B3 (High mobility group protein 2a) (HMG-2a) (High mobility group protein 4) (HMG-4)		
P05114	HMGN1_HUMAN	HMGN1 HMG14	Non-histone chromosomal protein HMG-14 (High mobility group nucleosome-binding domain-containing protein 1)		
P22626	ROA2_HUMAN	HNRNPA2B1 HNRPA2B1	Heterogeneous nuclear ribonucleoproteins A2/B1 (hnRNP A2/B1)	●	
P51991	ROA3_HUMAN	HNRNPA3 HNRPA3	Heterogeneous nuclear ribonucleoprotein A3 (hnRNP A3)	●	
Q99729	ROAA_HUMAN	HNRNPAB ABBP1 HNRPAB	Heterogeneous nuclear ribonucleoprotein A/B (hnRNP A/B) (APOBEC1-binding protein 1) (ABBP-1)	●	
Q14103	HNRPD_HUMAN	HNRNPD AUF1 HNRPD	Heterogeneous nuclear ribonucleoprotein D0 (hnRNP D0) (AU-rich element RNA-binding protein 1)	●	
P55795	HNRH2_HUMAN	HNRNPH2 FTP3 HNRPH2	Heterogeneous nuclear ribonucleoprotein H2 (hnRNP H2) (FTP-3) (Heterogeneous nuclear ribonucleoprotein H') (hnRNP H') [Cleaved into:	●	

			Heterogeneous nuclear ribonucleoprotein H2, N-terminally processed]		
P61978	HNRPK_HUMAN	HNRNPK HNRPK	Heterogeneous nuclear ribonucleoprotein K (hnRNP K) (Transformation up-regulated nuclear protein) (TUNP)	●	
Q9BUJ2	HNRL1_HUMAN	HNRNPUL1 E1BAP5 HNRPUL1	Heterogeneous nuclear ribonucleoprotein U-like protein 1 (Adenovirus early region 1B-associated protein 5) (E1B-55 kDa-associated protein 5) (E1B-AP5)	●	
P09651	ROA1_HUMAN	HNRNPA1 HNRPA1	Heterogeneous nuclear ribonucleoprotein A1 (hnRNP A1) (Helix-destabilizing protein) (Single-strand RNA-binding protein) (hnRNP core protein A1) [Cleaved into: Heterogeneous nuclear ribonucleoprotein A1, N-terminally processed]	●	
O60506	HNRPQ_HUMAN	SYNCRIP HNRPQ NSAP1	Heterogeneous nuclear ribonucleoprotein Q (hnRNP Q) (Glycine- and tyrosine-rich RNA-binding protein) (GRY-RBP) (NS1-associated protein 1) (Synaptotagmin-binding, cytoplasmic RNA-interacting protein)	●	
Q00613	HSF1_HUMAN	HSF1 HSTF1	Heat shock factor protein 1 (HSF 1) (Heat shock transcription factor 1) (HSTF 1)		
P07900	HS90A_HUMAN	HSP90AA1 HSP90A HSPC1 HSPCA	Heat shock protein HSP 90-alpha (EC 3.6.4.10) (Heat shock 86 kDa) (HSP 86) (HSP86) (Heat shock protein family C member 1) (Lipopolysaccharide-associated protein 2) (LAP-2) (LPS-associated protein 2) (Renal carcinoma antigen NY-REN-38)	●	●
P34932	HSP74_HUMAN	HSPA4 APG2 HSPH2	Heat shock 70 kDa protein 4 (HSP70RY) (Heat shock 70-related protein APG-2) (Heat shock protein family H member 2)	●	
P38646	GRP75_HUMAN	HSPA9 GRP75 HSPA9B mt-HSP70	Stress-70 protein, mitochondrial (75 kDa glucose-regulated protein) (GRP-75) (Heat shock 70 kDa protein 9) (Heat shock protein family A member 9) (Mortalin) (MOT) (Peptide-binding protein 74) (PBP74)		
P04792	HSPB1_HUMAN	HSPB1 HSP27 HSP28	Heat shock protein beta-1 (HspB1) (28 kDa heat shock protein) (Estrogen-regulated 24 kDa protein) (Heat shock 27 kDa protein) (HSP 27) (Heat shock protein family B member 1) (Stress-responsive protein 27) (SRP27)	●	●
P10809	CH60_HUMAN	HSPD1 HSP60	60 kDa heat shock protein, mitochondrial (EC 5.6.1.7) (60 kDa chaperonin) (Chaperonin 60) (CPN60) (Heat	●	

			shock protein 60) (HSP-60) (Hsp60) (Heat shock protein family D member 1) (HuCHA60) (Mitochondrial matrix protein P1) (P60 lymphocyte protein)		
Q9BYX4	IFIH1_HUMAN	IFIH1 MDA5 RH116	Interferon-induced helicase C domain-containing protein 1 (EC 3.6.4.13) (Clinically amyopathic dermatomyositis autoantigen 140 kDa) (CADM-140 autoantigen) (Helicase with 2 CARD domains) (Helicard) (Interferon-induced with helicase C domain protein 1) (Melanoma differentiation-associated protein 5) (MDA-5) (Murabutide down-regulated protein) (RIG-I-like receptor 2) (RLR-2) (RNA helicase-DEAD box protein 116)		●
Q9NZI8	IF2B1_HUMAN	IGF2BP1 CRDBP VICKZ1 ZBP1	Insulin-like growth factor 2 mRNA-binding protein 1 (IGF2 mRNA-binding protein 1) (IMP-1) (IMP1) (Coding region determinant-binding protein) (CRD-BP) (IGF-II mRNA-binding protein 1) (VICKZ family member 1) (Zipcode-binding protein 1) (ZBP-1)		
Q9Y6M1	IF2B2_HUMAN	IGF2BP2 IMP2 VICKZ2	Insulin-like growth factor 2 mRNA-binding protein 2 (IGF2 mRNA-binding protein 2) (IMP-2) (Hepatocellular carcinoma autoantigen p62) (IGF-II mRNA-binding protein 2) (VICKZ family member 2)		
O00425	IF2B3_HUMAN	IGF2BP3 IMP3 KOC1 VICKZ3	Insulin-like growth factor 2 mRNA-binding protein 3 (IGF2 mRNA-binding protein 3) (IMP-3) (IGF-II mRNA-binding protein 3) (KH domain-containing protein overexpressed in cancer) (hKOC) (VICKZ family member 3)		
Q9H8X2	IPPK_HUMAN	IPPK C9orf12	Inositol-pentakisphosphate 2-kinase (EC 2.7.1.158) (IPK1 homolog) (Inositol-1,3,4,5,6-pentakisphosphate 2-kinase) (Ins(1,3,4,5,6)P5 2-kinase) (InsP5 2-kinase)		
O95373	IPO7_HUMAN	IPO7 RANBP7	Importin-7 (Imp7) (Ran-binding protein 7) (RanBP7)		
O15397	IPO8_HUMAN	IPO8 RANBP8	Importin-8 (Imp8) (Ran-binding protein 8) (RanBP8)		
P05556	ITB1_HUMAN	ITGB1 FNRB MDF2 MSK12	Integrin beta-1 (Fibronectin receptor subunit beta) (Glycoprotein IIa) (GPIIA) (VLA-4 subunit beta) (CD antigen CD29)		●
Q63ZY3	KANK2_HUMAN	KANK2 ANKRD25 KIAA1518 MXRA3 SIP	KN motif and ankyrin repeat domain-containing protein 2 (Ankyrin repeat domain-containing protein 25) (Matrix-remodeling-associated protein 3) (SRC-1-interacting protein) (SIP) (SRC-interacting protein) (SRC1-interacting protein)		

Q07666	KHDR1_HUMAN	KHDRBS1 SAM68	KH domain-containing, RNA-binding, signal transduction-associated protein 1 (GAP-associated tyrosine phosphoprotein p62) (Src-associated in mitosis 68 kDa protein) (Sam68) (p21 Ras GTPase-activating protein-associated p62) (p68)		
O75525	KHDR3_HUMAN	KHDRBS3 SALP SLM2	KH domain-containing, RNA-binding, signal transduction-associated protein 3 (RNA-binding protein T-Star) (Sam68-like mammalian protein 2) (SLM-2) (Sam68-like phosphotyrosine protein)		
Q92945	FUBP2_HUMAN	KHSRP FUBP2	Far upstream element-binding protein 2 (FUSE-binding protein 2) (KH type-splicing regulatory protein) (KSRP) (p75)		
Q8TCG1	CIP2A_HUMAN	CIP2A KIAA1524	Protein CIP2A (Cancerous inhibitor of PP2A) (p90 autoantigen)		
Q02241	KIF23_HUMAN	KIF23 KNSL5 MKLP1	Kinesin-like protein KIF23 (Kinesin-like protein 5) (Mitotic kinesin-like protein 1)		
P33176	KINH_HUMAN	KIF5B KNS KNS1	Kinesin-1 heavy chain (Conventional kinesin heavy chain) (Ubiquitous kinesin heavy chain) (UKHC)		
Q07866	KLC1_HUMAN	KLC1 KLC KNS2	Kinesin light chain 1 (KLC 1)	●	
P52294	IMA5_HUMAN	KPNA1 RCH2	Importin subunit alpha-5 (Karyopherin subunit alpha-1) (Nucleoprotein interactor 1) (NPI-1) (RAG cohort protein 2) (SRP1-beta) [Cleaved into: Importin subunit alpha-5, N-terminally processed]	●	
P52292	IMA1_HUMAN	KPNA2 RCH1 SRP1	Importin subunit alpha-1 (Karyopherin subunit alpha-2) (RAG cohort protein 1) (SRP1-alpha)		●
O00505	IMA4_HUMAN	KPNA3 QIP2	Importin subunit alpha-4 (Importin alpha Q2) (Qip2) (Karyopherin subunit alpha-3) (SRP1-gamma)		●
O60684	IMA7_HUMAN	KPNA6 IPOA7	Importin subunit alpha-7 (Karyopherin subunit alpha-6)		●
Q14974	IMB1_HUMAN	KPNB1 NTF97	Importin subunit beta-1 (Importin-90) (Karyopherin subunit beta-1) (Nuclear factor p97) (Pore targeting complex 97 kDa subunit) (PTAC97)	●	
Q9UN81	LORF1_HUMAN	L1RE1 LRE1	LINE-1 retrotransposable element ORF1 protein (L1ORF1p) (LINE retrotransposable element 1) (LINE1 retrotransposable element 1)		
Q6PKG0	LARP1_HUMAN	LARP1 KIAA0731 LARP	La-related protein 1 (La ribonucleoprotein domain family member 1)		●
Q71RC2	LARP4_HUMAN	LARP4 PP13296	La-related protein 4 (La ribonucleoprotein domain family member 4)		

Q14739	LBR_HUMAN	LBR	Delta(14)-sterol reductase LBR (Delta-14-SR) (EC 1.3.1.70) (3-beta-hydroxysterol Delta (14)-reductase) (C-14 sterol reductase) (C14SR) (Integral nuclear envelope inner membrane protein) (LMN2R) (Lamin-B receptor) (Sterol C14-reductase)		
Q9H492	MLP3A_HUMAN	MAP1LC3A	Microtubule-associated protein 1 light chain 3 alpha (Autophagy-related protein LC3 A) (Autophagy-related ubiquitin-like modifier LC3 A) (MAP1 light chain 3-like protein 1) (Microtubule-associated proteins 1A/1B light chain 3A) (MAP1A/MAP1B LC3 A) (MAP1A/MAP1B light chain 3 A)		
Q9Y2U8	MAN1_HUMAN	LEMD3 MAN1	Inner nuclear membrane protein Man1 (LEM domain-containing protein 3)		
Q9H9Z2	LN28A_HUMAN	LIN28A CSDD1 LIN28 ZCCHC1	Protein lin-28 homolog A (Lin-28A) (Zinc finger CCHC domain-containing protein 1)		
P02545	LMNA_HUMAN	LMNA LMN1	Prelamin-A/C [Cleaved into: Lamin-A/C (70 kDa lamin) (Renal carcinoma antigen NY-REN-32)]		
Q93052	LPP_HUMAN	LPP	Lipoma-preferred partner (LIM domain-containing preferred translocation partner in lipoma)	●	
Q8ND56	LS14A_HUMAN	LSM14A C19orf13 FAM61A RAP55 RAP55A	Protein LSM14 homolog A (Protein FAM61A) (Protein SCD6 homolog) (Putative alpha-synuclein-binding protein) (AlphaSNBP) (RNA-associated protein 55A) (hRAP55) (hRAP55A)		●
Q9BX40	LS14B_HUMAN	LSM14B C20orf40 FAM61B RAP55B	Protein LSM14 homolog B (RNA-associated protein 55B) (hRAP55B)		
P62310	LSM3_HUMAN	LSM3 MDS017	U6 snRNA-associated Sm-like protein LSm3		
Q9NQ29	LUC7L_HUMAN	LUC7L LUC7L1	Putative RNA-binding protein Luc7-like 1 (Putative SR protein LUC7B1) (SR+89)		
Q86V48	LUZP1_HUMAN	LUZP1	Leucine zipper protein 1 (Filamin mechanobinding actin cross-linking protein) (Fimbacin)		
Q9UPN3	MACF1_HUMAN	MACF1 ABP620 ACF7 KIAA0465 KIAA0754 KIAA1251	Microtubule-actin cross-linking factor 1, isoforms 1/2/3/4/5 (620 kDa actin-binding protein) (ABP620) (Actin cross-linking family protein 7) (Macrophin-1) (Trabeculin-alpha)		
Q96JY0	MAEL_HUMAN	MAEL	Protein maelstrom homolog		
P43358	MAGA4_HUMAN	MAGEA4 MAGE4	Melanoma-associated antigen 4 (Cancer/testis antigen 1.4) (CT1.4) (MAGE-4 antigen) (MAGE-41 antigen) (MAGE-X2 antigen)	●	

Q9Y5V3	MAGD1_HUMAN	MAGED1 NRAGE PP2250 PRO2292	Melanoma-associated antigen D1 (MAGE tumor antigen CCF) (MAGE-D1 antigen) (Neurotrophin receptor-interacting MAGE homolog)		
Q9UNF1	MAGD2_HUMAN	MAGED2 BCG1	Melanoma-associated antigen D2 (11B6) (Breast cancer-associated gene 1 protein) (BCG-1) (Hepatocellular carcinoma-associated protein JCL-1) (MAGE-D2 antigen)		
Q96A72	MGN2_HUMAN	MAGOHB MAGOH2	Protein mago nashi homolog 2		
O14733	MP2K7_HUMAN	MAP2K7 JNKK2 MEK7 MKK7 PRKMK7 SKK4	Dual specificity mitogen-activated protein kinase kinase 7 (MAP kinase kinase 7) (MAPKK 7) (EC 2.7.12.2) (JNK-activating kinase 2) (MAPK/ERK kinase 7) (MEK 7) (Stress-activated protein kinase kinase 4) (SAPK kinase 4) (SAPKK-4) (SAPKK4) (c-Jun N-terminal kinase kinase 2) (JNK kinase 2) (JNKK 2)	●	
P27816	MAP4_HUMAN	MAP4	Microtubule-associated protein 4 (MAP-4)	●	
O95819	M4K4_HUMAN	MAP4K4 HGK KIAA0687 NIK	Mitogen-activated protein kinase kinase kinase kinase 4 (EC 2.7.11.1) (HPK/GCK-like kinase HGK) (MAPK/ERK kinase kinase kinase 4) (MEK kinase kinase 4) (MEKKK 4) (Nck-interacting kinase)	●	
P45983	MK08_HUMAN	MAPK8 JNK1 PRKM8 SAPK1 SAPK1C	Mitogen-activated protein kinase 8 (MAP kinase 8) (MAPK 8) (EC 2.7.11.24) (JNK-46) (Stress-activated protein kinase 1c) (SAPK1c) (Stress-activated protein kinase JNK1) (c-Jun N-terminal kinase 1)	●	
Q9UPT6	JIP3_HUMAN	MAPK8IP3 JIP3 KIAA1066	C-Jun-amino-terminal kinase-interacting protein 3 (JIP-3) (JNK-interacting protein 3) (JNK MAP kinase scaffold protein 3) (Mitogen-activated protein kinase 8-interacting protein 3)		
Q15691	MARE1_HUMAN	MAPRE1	Microtubule-associated protein RP/EB family member 1 (APC-binding protein EB1) (End-binding protein 1) (EB1)	●	
P56192	SYMC_HUMAN	MARS1 MARS	Methionine--tRNA ligase, cytoplasmic (EC 6.1.1.10) (Methionyl-tRNA synthetase) (MetRS)		
Q9NR56	MBNL1_HUMAN	MBNL1 EXP KIAA0428 MBNL	Muscleblind-like protein 1 (Triplet-expansion RNA-binding protein)		
P33991	MCM4_HUMAN	MCM4 CDC21	DNA replication licensing factor MCM4 (EC 3.6.4.12) (CDC21 homolog) (P1-CDC21)		

P33992	MCM5_HUMAN	MCM5 CDC46	DNA replication licensing factor MCM5 (EC 3.6.4.12) (CDC46 homolog) (P1-CDC46)		
P33993	MCM7_HUMAN	MCM7 CDC47 MCM2	DNA replication licensing factor MCM7 (EC 3.6.4.12) (CDC47 homolog) (P1.1-MCM3)		
P53582	MAP11_HUMAN	METAP1 KIAA0094	Methionine aminopeptidase 1 (MAP 1) (MetAP 1) (EC 3.4.11.18) (Peptidase M 1)		
A1L020	MEX3A_HUMAN	MEX3A RKHD4	RNA-binding protein MEX3A (RING finger and KH domain-containing protein 4)		
Q6ZN04	MEX3B_HUMAN	MEX3B KIAA2009 RKHD3 RNF195	RNA-binding protein MEX3B (RING finger and KH domain-containing protein 3) (RING finger protein 195)		
Q5U5Q3	MEX3C_HUMAN	MEX3C RKHD2 RNF194 BM-013	RNA-binding E3 ubiquitin-protein ligase MEX3C (EC 2.3.2.27) (RING finger and KH domain-containing protein 2) (RING finger protein 194) (RING-type E3 ubiquitin transferase MEX3C)		
P55081	MFAP1_HUMAN	MFAP1	Microfibrillar-associated protein 1 (Spliceosome B complex protein MFAP1)		
P46013	KI67_HUMAN	MKI67	Proliferation marker protein Ki-67 (Antigen identified by monoclonal antibody Ki-67) (Antigen KI-67) (Antigen Ki67)		
Q9HCE1	MOV10_HUMAN	MOV10 KIAA1631	Helicase MOV-10 (EC 3.6.4.13) (Armitage homolog) (Moloney leukemia virus 10 protein)		●
P52701	MSH6_HUMAN	MSH6 GTBP	DNA mismatch repair protein Msh6 (hMSH6) (G/T mismatch-binding protein) (GTBP) (GTMBP) (MutS protein homolog 6) (MutS-alpha 160 kDa subunit) (p160)		
O43347	MSI1H_HUMAN	MSI1	RNA-binding protein Musashi homolog 1 (Musashi-1)	●	
Q2M296	MTHSD_HUMAN	MTHFSD	Methenyltetrahydrofolate synthase domain-containing protein		
Q96DH6	MSI2H_HUMAN	MSI2	RNA-binding protein Musashi homolog 2 (Musashi-2)		
P11586	C1TC_HUMAN	MTHFD1 MTHFC MTHFD	C-1-tetrahydrofolate synthase, cytoplasmic (C1-THF synthase) (Epididymis secretory sperm binding protein) [Cleaved into: C-1-tetrahydrofolate synthase, cytoplasmic, N-terminally processed] [Includes: Methylenetetrahydrofolate dehydrogenase (EC 1.5.1.5); Methenyltetrahydrofolate cyclohydrolase (EC 3.5.4.9); Formyltetrahydrofolate synthetase (EC 6.3.4.3)]	●	

P42345	MTOR_HUMAN	MTOR FRAP FRAP1 FRAP2 RAFT1 RAPT1	Serine/threonine-protein kinase mTOR (EC 2.7.11.1) (FK506-binding protein 12-rapamycin complex-associated protein 1) (FKBP12-rapamycin complex-associated protein) (Mammalian target of rapamycin) (mTOR) (Mechanistic target of rapamycin) (Rapamycin and FKBP12 target 1) (Rapamycin target protein 1) (Tyrosine-protein kinase mTOR) (EC 2.7.10.2)		
Q9UM54	MYO6_HUMAN	MYO6 KIAA0389	Unconventional myosin-VI (Unconventional myosin-6)	●	
Q9Y6Q9	NCOA3_HUMAN	NCOA3 AIB1 BHLHE42 RAC3 TRAM1	Nuclear receptor coactivator 3 (NCoA-3) (EC 2.3.1.48) (ACTR) (Amplified in breast cancer 1 protein) (AIB-1) (CBP-interacting protein) (pCIP) (Class E basic helix-loop-helix protein 42) (bHLHe42) (Receptor-associated coactivator 3) (RAC-3) (Steroid receptor coactivator protein 3) (SRC-3) (Thyroid hormone receptor activator molecule 1) (TRAM-1)		
Q0ZGT2	NEXN_HUMAN	NEXN	Nexilin (F-actin-binding protein) (Nelin)		
Q15233	NONO_HUMAN	NONO NRB54	Non-POU domain-containing octamer-binding protein (NonO protein) (54 kDa nuclear RNA- and DNA-binding protein) (p54(nrb)) (p54nrb) (55 kDa nuclear protein) (NMT55) (DNA-binding p52/p100 complex, 52 kDa subunit)	●	
Q9Y2X3	NOP58_HUMAN	NOP58 NOL5 NOP5 HSPC120	Nucleolar protein 58 (Nucleolar protein 5)		
Q9Y314	NOSIP_HUMAN	NOSIP CGI-25	Nitric oxide synthase-interacting protein (E3 ubiquitin-protein ligase NOSIP) (EC 2.3.2.27) (RING-type E3 ubiquitin transferase NOSIP) (eNOS-interacting protein)		
Q08J23	NSUN2_HUMAN	NSUN2 SAKI TRM4	RNA cytosine C(5)-methyltransferase NSUN2 (EC 2.1.1.-) (Myc-induced SUN domain-containing protein) (Misu) (NOL1/NOP2/Sun domain family member 2) (Substrate of AIM1/Aurora kinase B) (mRNA cytosine C(5)-methyltransferase) (EC 2.1.1.-) (tRNA cytosine C(5)-methyltransferase) (EC 2.1.1.-, EC 2.1.1.203) (tRNA methyltransferase 4 homolog) (hTrm4)	●	
Q9BV86	NTM1A_HUMAN	NTMT1 C9orf32 METTL11A	N-terminal Xaa-Pro-Lys N-methyltransferase 1 (EC 2.1.1.244) (Alpha N-terminal protein methyltransferase 1A) (Methyltransferase-like protein 11A) (N-terminal RCC1 methyltransferase) (X-Pro-		

		NRMT NRMT1 AD-003	Lys N-terminal protein methyltransferase 1A) (NTM1A) [Cleaved into: N-terminal Xaa-Pro-Lys N-methyltransferase 1, N-terminally processed]		
Q9Y266	NUDC_HUMAN	NUDC	Nuclear migration protein nudC (Nuclear distribution protein C homolog)	●	
Q7Z417	NUFP2_HUMAN	NUFIP2 KIAA1321 PIG1	FMR1-interacting protein NUFIP2 (82 kDa FMRP-interacting protein) (82-FIP) (Cell proliferation-inducing gene 1 protein) (FMRP-interacting protein 2) (Nuclear FMR1-interacting protein 2)	●	
Q92621	NU205_HUMAN	NUP205 C7orf14 KIAA0225	Nuclear pore complex protein Nup205 (205 kDa nucleoporin) (Nucleoporin Nup205)		
P52948	NUP98_HUMAN	NUP98 ADAR2	Nuclear pore complex protein Nup98-Nup96 (EC 3.4.21.-) [Cleaved into: Nuclear pore complex protein Nup98 (98 kDa nucleoporin) (Nucleoporin Nup98) (Nup98); Nuclear pore complex protein Nup96 (96 kDa nucleoporin) (Nucleoporin Nup96) (Nup96)]		
Q9UBU9	NXF1_HUMAN	NXF1 TAP	Nuclear RNA export factor 1 (Tip-associated protein) (Tip-associating protein) (mRNA export factor TAP)		
Q8N543	OGFD1_HUMAN	OGFOD1 KIAA1612 TPA1	Prolyl 3-hydroxylase OGFOD1 (EC 1.14.11.-) (2-oxoglutarate and iron-dependent oxygenase domain-containing protein 1) (Termination and polyadenylation 1 homolog) (uS12 prolyl 3-hydroxylase)		
O15527	OGG1_HUMAN	OGG1 MMH MUTM OGH1	N-glycosylase/DNA lyase [Includes: 8-oxoguanine DNA glycosylase (EC 3.2.2.-); DNA-(apurinic or apyrimidinic site) lyase (AP lyase) (EC 4.2.99.18)]		
Q96CV9	OPTN_HUMAN	OPTN FIP2 GLC1E HIP7 HYPL NRP	Optineurin (E3-14.7K-interacting protein) (FIP-2) (Huntingtin yeast partner L) (Huntingtin-interacting protein 7) (HIP-7) (Huntingtin-interacting protein L) (NEMO-related protein) (Optic neuropathy-inducing protein) (Transcription factor IIIA-interacting protein) (TFIIIA-IntP)		
Q9UQ80	PA2G4_HUMAN	PA2G4 EBP1	Proliferation-associated protein 2G4 (Cell cycle protein p38-2G4 homolog) (hG4-1) (ErbB3-binding protein 1)	●	
P11940	PABP1_HUMAN	PABPC1 PAB1 PABP PABP1 PABPC2	Polyadenylate-binding protein 1 (PABP-1) (Poly(A)-binding protein 1)	●	●

Q13310	PABP4_HUMAN	PABPC4 APP1 PABP4	Polyadenylate-binding protein 4 (PABP-4) (Poly(A)-binding protein 4) (Activated-platelet protein 1) (APP-1) (Inducible poly(A)-binding protein) (iPABP)		
Q9H361	PABP3_HUMAN	PABPC3 PABP3 PABPL3	Polyadenylate-binding protein 3 (PABP-3) (Poly(A)-binding protein 3) (Testis-specific poly(A)-binding protein)		
O96013	PAK4_HUMAN	PAK4 KIAA1142	Serine/threonine-protein kinase PAK 4 (EC 2.7.11.1) (p21-activated kinase 4) (PAK-4)		
Q8WX93	PALLD_HUMAN	PALLD KIAA0992 CGI-151	Palladin (SIH002) (Sarcoma antigen NY-SAR-77)		
Q86W56	PARG_HUMAN	PARG	Poly(ADP-ribose) glycohydrolase (EC 3.2.1.143)		
P09874	PARP1_HUMAN	PARP1 ADPRT PPOL	Poly [ADP-ribose] polymerase 1 (PARP-1) (EC 2.4.2.30) (ADP-ribosyltransferase diphtheria toxin-like 1) (ARTD1) (DNA ADP-ribosyltransferase PARP1) (EC 2.4.2.-) (NAD(+) ADP-ribosyltransferase 1) (ADPRT 1) (Poly[ADP-ribose] synthase 1) (Protein poly-ADP-ribosyltransferase PARP1) (EC 2.4.2.-) [Cleaved into: Poly [ADP-ribose] polymerase 1, processed C-terminus (Poly [ADP-ribose] polymerase 1, 89-kDa form); Poly [ADP-ribose] polymerase 1, processed N-terminus (NT-PARP-1) (Poly [ADP-ribose] polymerase 1, 24-kDa form) (Poly [ADP-ribose] polymerase 1, 28-kDa form)]	●	
Q9H0J9	PAR12_HUMAN	PARP12 ZC3HDC1	Protein mono-ADP-ribosyltransferase PARP12 (EC 2.4.2.-) (ADP-ribosyltransferase diphtheria toxin-like 12) (ARTD12) (Poly [ADP-ribose] polymerase 12) (PARP-12) (Zinc finger CCCH domain-containing protein 1)		
Q460N5	PAR14_HUMAN	PARP14 BAL2 KIAA1268	Protein mono-ADP-ribosyltransferase PARP14 (EC 2.4.2.-) (ADP-ribosyltransferase diphtheria toxin-like 8) (ARTD8) (B aggressive lymphoma protein 2) (Poly [ADP-ribose] polymerase 14) (PARP-14)		
Q460N3	PAR15_HUMAN	PARP15 BAL3	Protein mono-ADP-ribosyltransferase PARP15 (EC 2.4.2.-) (ADP-ribosyltransferase diphtheria toxin-like 7) (ARTD7) (B-aggressive lymphoma protein 3) (Poly [ADP-ribose] polymerase 15) (PARP-15)		
Q96IZ0	PAWR_HUMAN	PAWR PAR4	PRKC apoptosis WT1 regulator protein (Prostate apoptosis response 4 protein) (Par-4)		

Q15366	PCBP2_HUMAN	PCBP2	Poly(rC)-binding protein 2 (Alpha-CP2) (Heterogeneous nuclear ribonucleoprotein E2) (hnRNP E2)		●
P12004	PCNA_HUMAN	PCNA	Proliferating cell nuclear antigen (PCNA) (Cyclin)		
Q8WUM4	PDC6I_HUMAN	PDCD6IP AIP1 ALIX KIAA1375	Programmed cell death 6-interacting protein (PDCD6-interacting protein) (ALG-2-interacting protein 1) (ALG-2-interacting protein X) (Hp95)		●
O00151	PDLI1_HUMAN	PDLIM1 CLIM1 CLP36	PDZ and LIM domain protein 1 (C-terminal LIM domain protein 1) (Elfin) (LIM domain protein CLP-36)		
P50479	PDLI4_HUMAN	PDLIM4 RIL	PDZ and LIM domain protein 4 (LIM protein RIL) (Reversion-induced LIM protein)	●	
Q96HC4	PDLI5_HUMAN	PDLIM5 ENH L9	PDZ and LIM domain protein 5 (Enigma homolog) (Enigma-like PDZ and LIM domains protein)		
Q9NTI5	PDS5B_HUMAN	PDS5B APRIN AS3 KIAA0979	Sister chromatid cohesion protein PDS5 homolog B (Androgen-induced proliferation inhibitor) (Androgen-induced prostate proliferative shutoff-associated protein AS3)		
Q9BRX2	PELO_HUMAN	PELO CGI-17	Protein pelota homolog (hPelota) (Protein Dom34 homolog)		
Q9NQP4	PFD4_HUMAN	PFDN4 PFD4	Prefoldin subunit 4 (Protein C-1)		
P07737	PROF1_HUMAN	PFN1	Profilin-1 (Epididymis tissue protein Li 184a) (Profilin I)		
P35080	PROF2_HUMAN	PFN2	Profilin-2 (Profilin II)	●	
Q96HS1	PGAM5_HUMAN	PGAM5	Serine/threonine-protein phosphatase PGAM5, mitochondrial (EC 3.1.3.16) (Bcl-XL-binding protein v68) (Phosphoglycerate mutase family member 5)		
Q99623	PHB2_HUMAN	PHB2 BAP REA	Prohibitin-2 (B-cell receptor-associated protein BAP37) (D-prohibitin) (Repressor of estrogen receptor activity)		●
Q86SQ0	PHLB2_HUMAN	PHLDB2 LL5B	Pleckstrin homology-like domain family B member 2 (Protein LL5-beta)		
P17252	KPCA_HUMAN	PRKCA PKCA PRKACA	Protein kinase C alpha type (PKC-A) (PKC-alpha) (EC 2.7.11.13)	●	
Q13835	PKP1_HUMAN	PKP1	Plakophilin-1 (Band 6 protein) (B6P)		
Q99959	PKP2_HUMAN	PKP2	Plakophilin-2		
Q9Y446	PKP3_HUMAN	PKP3	Plakophilin-3		
P30876	RPB2_HUMAN	POLR2B	DNA-directed RNA polymerase II subunit RPB2 (EC 2.7.7.6) (3'-5' exoribonuclease) (EC 3.1.13.-) (DNA-directed RNA polymerase II 140 kDa polypeptide)		

			(DNA-directed RNA polymerase II subunit B) (RNA polymerase II subunit 2) (RNA polymerase II subunit B2) (RNA-directed RNA polymerase II subunit RPB2) (EC 2.7.7.48)		
Q6PFW1	VIP1_HUMAN	PPIP5K1 HISPPD2A IP6K IPS1 KIAA0377 VIP1	Inositol hexakisphosphate and diphosphoinositol-pentakisphosphate kinase 1 (EC 2.7.4.24) (Diphosphoinositol pentakisphosphate kinase 1) (Histidine acid phosphatase domain-containing protein 2A) (IP6 kinase) (Inositol pyrophosphate synthase 1) (InsP6 and PP-IP5 kinase 1) (VIP1 homolog) (hsVIP1)		
Q9Y570	PPME1_HUMAN	PPME1 PME1 PP2593 PRO0750	Protein phosphatase methylesterase 1 (PME-1) (EC 3.1.1.89)		
Q96QC0	PP1RA_HUMAN	PPP1R10 CAT53 FB19 PNUTS	Serine/threonine-protein phosphatase 1 regulatory subunit 10 (MHC class I region proline-rich protein CAT53) (PP1-binding protein of 114 kDa) (Phosphatase 1 nuclear targeting subunit) (Protein FB19) (p99)		
Q6NYC8	PPR18_HUMAN	PPP1R18 HKMT1098 KIAA1949	Phostensin (Protein phosphatase 1 F-actin cytoskeleton-targeting subunit) (Protein phosphatase 1 regulatory subunit 18)		
P30153	2AAA_HUMAN	PPP2R1A	Serine/threonine-protein phosphatase 2A 65 kDa regulatory subunit A alpha isoform (PP2Aa) (Medium tumor antigen-associated 61 kDa protein) (PP2A subunit A isoform PR65-alpha) (PP2A subunit A isoform R1-alpha)	●	
O60828	PQBP1_HUMAN	PQBP1 NPW38 JM26	Polyglutamine-binding protein 1 (PQBP-1) (38 kDa nuclear protein containing a WW domain) (Npw38) (Polyglutamine tract-binding protein 1)		●
Q06830	PRDX1_HUMAN	PRDX1 PAGA PAGB TDPX2	Peroxiredoxin-1 (EC 1.11.1.24) (Natural killer cell-enhancing factor A) (NKEF-A) (Proliferation-associated gene protein) (PAG) (Thioredoxin peroxidase 2) (Thioredoxin-dependent peroxide reductase 2) (Thioredoxin-dependent peroxiredoxin 1)	●	
P30041	PRDX6_HUMAN	PRDX6 AOP2 KIAA0106	Peroxiredoxin-6 (EC 1.11.1.27) (1-Cys peroxiredoxin) (1-Cys PRX) (24 kDa protein) (Acidic calcium-independent phospholipase A2) (aiPLA2) (EC 3.1.1.4) (Antioxidant protein 2) (Glutathione-dependent peroxiredoxin) (Liver 2D page spot 40) (Lysophosphatidylcholine acyltransferase 5) (LPC acyltransferase 5) (LPCAT-5) (Lyso-PC	●	

			acyltransferase 5) (EC 2.3.1.23) (Non-selenium glutathione peroxidase) (NSGPx) (Red blood cells page spot 12)		
O75569	PRKRA_HUMAN	PRKRA PACT RAX HSD-14 HSD14	Interferon-inducible double-stranded RNA-dependent protein kinase activator A (PKR-associated protein X) (PKR-associating protein X) (Protein activator of the interferon-induced protein kinase) (Protein kinase, interferon-inducible double-stranded RNA-dependent activator)	●	●
Q99873	ANM1_HUMAN	PRMT1 HMT2 HRMT1L2 IR1B4	Protein arginine N-methyltransferase 1 (EC 2.1.1.319) (Histone-arginine N-methyltransferase PRMT1) (Interferon receptor 1-bound protein 4)		●
O14744	ANM5_HUMAN	PRMT5 HRMT1L5 IBP72 JBP1 SKB1	Protein arginine N-methyltransferase 5 (PRMT5) (EC 2.1.1.320) (72 kDa ICln-binding protein) (Histone-arginine N-methyltransferase PRMT5) (Jak-binding protein 1) (Shk1 kinase-binding protein 1 homolog) (SKB1 homolog) (SKB1Hs) [Cleaved into: Protein arginine N-methyltransferase 5, N-terminally processed]		
P48634	PRC2A_HUMAN	PRRC2A BAT2 G2	Protein PRRC2A (HLA-B-associated transcript 2) (Large proline-rich protein BAT2) (Proline-rich and coiled-coil-containing protein 2A) (Protein G2)		
Q9Y520	PRC2C_HUMAN	PRRC2C BAT2D1 BAT2L2 KIAA1096 XTP2	Protein PRRC2C (BAT2 domain-containing protein 1) (HBV X-transactivated gene 2 protein) (HBV XAg-transactivated protein 2) (HLA-B-associated transcript 2-like 2) (Proline-rich and coiled-coil-containing protein 2C)		
O95758	PTBP3_HUMAN	PTBP3 ROD1	Polypyrimidine tract-binding protein 3 (Regulator of differentiation 1) (Rod1)		
Q15185	TEBP_HUMAN	PTGES3 P23 TEBP	Prostaglandin E synthase 3 (EC 5.3.99.3) (Cytosolic prostaglandin E2 synthase) (cPGES) (Hsp90 co-chaperone) (Progesterone receptor complex p23) (Telomerase-binding protein p23)	●	
Q05397	FAK1_HUMAN	PTK2 FAK FAK1	Focal adhesion kinase 1 (FADK 1) (EC 2.7.10.2) (Focal adhesion kinase-related nonkinase) (FRNK) (Protein phosphatase 1 regulatory subunit 71) (PPP1R71) (Protein-tyrosine kinase 2) (p125FAK) (pp125FAK)		
Q14671	PUM1_HUMAN	PUM1 KIAA0099 PUMH1	Pumilio homolog 1 (HsPUM) (Pumilio-1)		

Q8TB72	PUM2_HUMAN	PUM2 KIAA0235 PUMH2	Pumilio homolog 2 (Pumilio-2)		
Q00577	PURA_HUMAN	PURA PUR1	Transcriptional activator protein Pur-alpha (Purine-rich single-stranded DNA-binding protein alpha)	●	
Q96QR8	PURB_HUMAN	PURB	Transcriptional regulator protein Pur-beta (Purine-rich element-binding protein B)		
A1KZ92	PXDNL_HUMAN	PXDNL VPO2	Probable oxidoreductase PXDNL (EC 1.-.-.-) (Cardiac peroxidase) (Inactive peroxidasin-like protein) (Polysomal ribonuclease 1) (PRM1) (Vascular peroxidase 2)		
P32322	P5CR1_HUMAN	PYCR1	Pyrroline-5-carboxylate reductase 1, mitochondrial (P5C reductase 1) (P5CR 1) (EC 1.5.1.2)		
Q96PU8	QKI_HUMAN	QKI HKQ	KH domain-containing RNA-binding protein QKI (Protein quaking) (Hqk) (HqkI)		
P62820	RAB1A_HUMAN	RAB1A RAB1	Ras-related protein Rab-1A (EC 3.6.5.2) (YPT1-related protein)	●	●
Q9H0H5	RGAP1_HUMAN	RACGAP1 KIAA1478 MGCRACGAP	Rac GTPase-activating protein 1 (Male germ cell RacGap) (MgcRacGAP) (Protein CYK4 homolog) (CYK4) (HsCYK-4)		
P63244	RACK1_HUMAN	RACK1 GNB2L1 HLC7 PIG21	Small ribosomal subunit protein RACK1 (Cell proliferation-inducing gene 21 protein) (Guanine nucleotide-binding protein subunit beta-2-like 1) (Guanine nucleotide-binding protein subunit beta-like protein 12.3) (Human lung cancer oncogene 7 protein) (HLC-7) (Receptor for activated C kinase) (Receptor of activated protein C kinase 1) [Cleaved into: Small ribosomal subunit protein RACK1, N-terminally processed (Guanine nucleotide-binding protein subunit beta-2-like 1, N-terminally processed) (Receptor of activated protein C kinase 1, N-terminally processed)]		
O60216	RAD21_HUMAN	RAD21 HR21 KIAA0078 NXP1 SCC1	Double-strand-break repair protein rad21 homolog (hHR21) (Nuclear matrix protein 1) (NXP-1) (SCC1 homolog) [Cleaved into: 64-kDa C-terminal product (64-kDa carboxy-terminal product) (65-kDa carboxy-terminal product)]		
P43487	RANG_HUMAN	RANBP1	Ran-specific GTPase-activating protein (Ran-binding protein 1) (RanBP1)	●	
Q8N122	RPTOR_HUMAN	RPTOR KIAA1303 RAPTOR	Regulatory-associated protein of mTOR (Raptor) (p150 target of rapamycin (TOR)-scaffold protein)		

Q09028	RBBP4_HUMAN	RBBP4 RBAP48	Histone-binding protein RBBP4 (Chromatin assembly factor 1 subunit C) (CAF-1 subunit C) (Chromatin assembly factor I p48 subunit) (CAF-I 48 kDa subunit) (CAF-I p48) (Nucleosome-remodeling factor subunit RBAP48) (Retinoblastoma-binding protein 4) (RBBP-4) (Retinoblastoma-binding protein p48)		
Q9NWB1	RFOX1_HUMAN	RBFOX1 A2BP A2BP1 FOX1 HRNBP1	RNA binding protein fox-1 homolog 1 (Ataxin-2-binding protein 1) (Fox-1 homolog A) (Hexaribonucleotide-binding protein 1)		
O43251	RFOX2_HUMAN	RBFOX2 FOX2 HRNBP2 RBM9 RTA	RNA binding protein fox-1 homolog 2 (Fox-1 homolog B) (Hexaribonucleotide-binding protein 2) (RNA-binding motif protein 9) (RNA-binding protein 9) (Repressor of tamoxifen transcriptional activity)		
Q8IXT5	RB12B_HUMAN	RBM12B	RNA-binding protein 12B (RNA-binding motif protein 12B)		
Q5T8P6	RBM26_HUMAN	RBM26 C13orf10 PRO1777	RNA-binding protein 26 (CTCL tumor antigen se70-2) (RNA-binding motif protein 26)		
Q9BTD8	RBM42_HUMAN	RBM42	RNA-binding protein 42 (RNA-binding motif protein 42)		
P29558	RBMS1_HUMAN	RBMS1 C2orf12 MSSP MSSP1 SCR2	RNA-binding motif, single-stranded-interacting protein 1 (Single-stranded DNA-binding protein MSSP-1) (Suppressor of CDC2 with RNA-binding motif 2)		
Q15434	RBMS2_HUMAN	RBMS2 SCR3	RNA-binding motif, single-stranded-interacting protein 2 (Suppressor of CDC2 with RNA-binding motif 3)		
Q93062	RBPMS_HUMAN	RBPMS HERMES	RNA-binding protein with multiple splicing (RBP-MS) (RBPMS) (Heart and RRM expressed sequence) (Hermes)		
P18754	RCC1_HUMAN	RCC1 CHC1	Regulator of chromosome condensation (Cell cycle regulatory protein) (Chromosome condensation protein 1)		●
Q9P258	RCC2_HUMAN	RCC2 KIAA1470 TD60	Protein RCC2 (RCC1-like protein TD-60) (Telophase disk protein of 60 kDa)		
Q13123	RED_HUMAN	IK RED RER	Protein Red (Cytokine IK) (IK factor) (Protein RER)		
Q9HAU5	RENT2_HUMAN	UPF2 KIAA1408 RENT2	Regulator of nonsense transcripts 2 (Up-frameshift suppressor 2 homolog) (hUpf2)		

P40938	RFC3_HUMAN	RFC3	Replication factor C subunit 3 (Activator 1 38 kDa subunit) (A1 38 kDa subunit) (Activator 1 subunit 3) (Replication factor C 38 kDa subunit) (RF-C 38 kDa subunit) (RFC38)		
P35249	RFC4_HUMAN	RFC4	Replication factor C subunit 4 (Activator 1 37 kDa subunit) (A1 37 kDa subunit) (Activator 1 subunit 4) (Replication factor C 37 kDa subunit) (RF-C 37 kDa subunit) (RFC37)		
A6NKT7	RGPD3_HUMAN	RGPD3 RGP3	RanBP2-like and GRIP domain-containing protein 3		
P61586	RHOA_HUMAN	RHOA ARH12 ARHA RHO12	Transforming protein RhoA (EC 3.6.5.2) (Rho cDNA clone 12) (h12)		
Q8ND24	RN214_HUMAN	RNF214	RING finger protein 214		
P13489	RINI_HUMAN	RNH1 PRI RNH	Ribonuclease inhibitor (Placental ribonuclease inhibitor) (Placental RNase inhibitor) (Ribonuclease/angiogenin inhibitor 1) (RAI)		
Q13464	ROCK1_HUMAN	ROCK1	Rho-associated protein kinase 1 (EC 2.7.11.1) (Renal carcinoma antigen NY-REN-35) (Rho-associated, coiled-coil-containing protein kinase 1) (Rho-associated, coiled-coil-containing protein kinase I) (ROCK-I) (p160 ROCK-1) (p160ROCK)		
Q5TC82	RC3H1_HUMAN	RC3H1 KIAA2025 RNF198	Roquin-1 (Roquin) (EC 2.3.2.27) (RING finger and C3H zinc finger protein 1) (RING finger and CCCH-type zinc finger domain-containing protein 1) (RING finger protein 198)		
P39019	RS19_HUMAN	RPS19	Small ribosomal subunit protein eS19 (40S ribosomal protein S19)	●	
P15880	RS2_HUMAN	RPS2 RPS4	Small ribosomal subunit protein uS5 (40S ribosomal protein S2) (40S ribosomal protein S4) (Protein LLRep3)		
P23396	RS3_HUMAN	RPS3 OK/SW-cl.26	Small ribosomal subunit protein uS3 (40S ribosomal protein S3) (EC 4.2.99.18)	●	
P61247	RS3A_HUMAN	RPS3A FTE1 MFTL	Small ribosomal subunit protein eS1 (40S ribosomal protein S3a) (v-fos transformation effector protein) (Fte-1)	●	
P62753	RS6_HUMAN	RPS6 OK/SW-cl.2	Small ribosomal subunit protein eS6 (40S ribosomal protein S6) (Phosphoprotein NP33)	●	
P51812	KS6A3_HUMAN	RPS6KA3 ISPK1 MAPKAPK1B RSK2	Ribosomal protein S6 kinase alpha-3 (S6K-alpha-3) (EC 2.7.11.1) (90 kDa ribosomal protein S6 kinase 3) (p90-RSK 3) (p90RSK3) (Insulin-stimulated protein kinase 1) (ISPK-1) (MAP kinase-activated protein kinase 1b) (MAPK-activated protein kinase 1b)	●	

			(MAPKAP kinase 1b) (MAPKAPK-1b) (Ribosomal S6 kinase 2) (RSK-2) (pp90RSK2)		
O76021	RL1D1_HUMAN	RSL1D1 CATX11 CSIG PBK1 L12	Ribosomal L1 domain-containing protein 1 (CATX-11) (Cellular senescence-inhibited gene protein) (Protein PBK1)		
Q13200	PSMD2_HUMAN	PSMD2 TRAP2	26S proteasome non-ATPase regulatory subunit 2 (26S proteasome regulatory subunit RPN1) (26S proteasome regulatory subunit S2) (26S proteasome subunit p97) (Protein 55.11) (Tumor necrosis factor type 1 receptor-associated protein 2)	●	
Q9Y3I0	RTCB_HUMAN	RTCB C22orf28 HSPC117	RNA-splicing ligase RtcB homolog (EC 6.5.1.8) (3'-phosphate/5'-hydroxy nucleic acid ligase)		
Q9Y224	RTRAF_HUMAN	RTRAF C14orf166 CGI-99	RNA transcription, translation and transport factor protein (CLE7 homolog) (CLE) (hCLE)		
Q86SG5	S1A7A_HUMAN	S100A7A S100A15 S100A7L1	Protein S100-A7A (S100 calcium-binding protein A15) (S100 calcium-binding protein A7-like 1) (S100 calcium-binding protein A7A)		
P06702	S10A9_HUMAN	S100A9 CAGB CFAG MRP14	Protein S100-A9 (Calgranulin-B) (Calprotectin L1H subunit) (Leukocyte L1 complex heavy chain) (Migration inhibitory factor-related protein 14) (MRP-14) (p14) (S100 calcium-binding protein A9)		
Q14151	SAFB2_HUMAN	SAFB2 KIAA0138	Scaffold attachment factor B2 (SAF-B2)		
P53992	SC24C_HUMAN	SEC24C KIAA0079	Protein transport protein Sec24C (SEC24-related protein C)	●	
Q8NC51	SERB1_HUMAN	SERBP1 PAIRBP1 CGI-55	SERPINE1 mRNA-binding protein 1 (PAI1 RNA-binding protein 1) (PAI-RBP1) (Plasminogen activator inhibitor 1 RNA-binding protein)	●	
P31947	1433S_HUMAN	SFN HME1	14-3-3 protein sigma (Epithelial cell marker protein 1) (Stratifin)	●	
P23246	SFPQ_HUMAN	SFPQ PSF	Splicing factor, proline- and glutamine-rich (100 kDa DNA-pairing protein) (hPOMp100) (DNA-binding p52/p100 complex, 100 kDa subunit) (Polypyrimidine tract-binding protein-associated-splicing factor) (PSF) (PTB-associated-splicing factor)	●	
P84103	SRSF3_HUMAN	SRSF3 SFRS3 SRP20	Serine/arginine-rich splicing factor 3 (Pre-mRNA-splicing factor SRP20) (Splicing factor, arginine/serine-rich 3)		

Q9NUQ6	SPS2L_HUMAN	SPATS2L DNAPTP6 SP1224	SPATS2-like protein (DNA polymerase-transactivated protein 6) (Stress granule and nucleolar protein) (SGNP)		
O43166	SI1L1_HUMAN	SIPA1L1 E6TP1 KIAA0440	Signal-induced proliferation-associated 1-like protein 1 (SIPA1-like protein 1) (High-risk human papilloma viruses E6 oncoproteins targeted protein 1) (E6-targeted protein 1)		
Q8N6T7	SIR6_HUMAN	SIRT6 SIR2L6	NAD-dependent protein deacylase sirtuin-6 (EC 2.3.1.-) (NAD-dependent protein deacetylase sirtuin-6) (EC 2.3.1.286) (Protein mono-ADP-ribosyltransferase sirtuin-6) (EC 2.4.2.-) (Regulatory protein SIR2 homolog 6) (hSIRT6) (SIR2-like protein 6)		
P48029	SC6A8_HUMAN	SLC6A8	Sodium- and chloride-dependent creatine transporter 1 (CT1) (Creatine transporter 1) (Solute carrier family 6 member 8)	●	
P28370	SMCA1_HUMAN	SMARCA1 SNF2L SNF2L1	Probable global transcription activator SNF2L1 (EC 3.6.4.-) (ATP-dependent helicase SMARCA1) (Nucleosome-remodeling factor subunit SNF2L) (SWI/SNF-related matrix-associated actin-dependent regulator of chromatin subfamily A member 1)		
Q9UPU9	SMAG1_HUMAN	SAMD4A KIAA1053 SAMD4 SMAUG1	Protein Smaug homolog 1 (Smaug 1) (hSmaug1) (Sterile alpha motif domain-containing protein 4A) (SAM domain-containing protein 4A)		
Q9NTJ3	SMC4_HUMAN	SMC4 CAPC SMC4L1	Structural maintenance of chromosomes protein 4 (SMC protein 4) (SMC-4) (Chromosome-associated polypeptide C) (hCAP-C) (XCAP-C homolog)		
Q96Q15	SMG1_HUMAN	SMG1 ATX KIAA0421 LIP	Serine/threonine-protein kinase SMG1 (SMG-1) (hSMG-1) (EC 2.7.11.1) (Lambda/iota protein kinase C-interacting protein) (Lambda-interacting protein) (Nonsense mediated mRNA decay-associated PI3K-related kinase SMG1)		
Q16637	SMN_HUMAN	SMN1 SMN SMNT; SMN2 SMNC	Survival motor neuron protein (Component of gems 1) (Gemin-1)		
Q2TAY7	SMU1_HUMAN	SMU1	WD40 repeat-containing protein SMU1 (Smu-1 suppressor of mec-8 and unc-52 protein homolog) [Cleaved into: WD40 repeat-containing protein SMU1, N-terminally processed]	●	
Q7KZF4	SND1_HUMAN	SND1 TDRD11	Staphylococcal nuclease domain-containing protein 1 (EC 3.1.31.1) (100 kDa coactivator) (EBNA2		

			coactivator p100) (Tudor domain-containing protein 11) (p100 co-activator)		
P62306	RUXF_HUMAN	SNRPF PBSCF	Small nuclear ribonucleoprotein F (snRNP-F) (Sm protein F) (Sm-F) (SmF)		
Q13425	SNTB2_HUMAN	SNTB2 D16S2531E SNT2B2 SNTL	Beta-2-syntrophin (59 kDa dystrophin-associated protein A1 basic component 2) (Syntrophin-3) (SNT3) (Syntrophin-like) (SNTL)		
Q9BX66	SRBS1_HUMAN	SORBS1 KIAA0894 KIAA1296 SH3D5	Sorbin and SH3 domain-containing protein 1 (Ponsin) (SH3 domain protein 5) (SH3P12) (c-Cbl-associated protein) (CAP)	●	
O60504	VINEX_HUMAN	SORBS3 SCAM1	Vinexin (SH3-containing adapter molecule 1) (SCAM-1) (Sorbin and SH3 domain-containing protein 3)		
Q96R06	SPAG5_HUMAN	SPAG5	Sperm-associated antigen 5 (Astrin) (Deepest) (Mitotic spindle-associated protein p126) (MAP126)		
Q69YQ0	CYTSA_HUMAN	SPECC1L CYTSA KIAA0376	Cytospin-A (Renal carcinoma antigen NY-REN-22) (Sperm antigen with calponin homology and coiled-coil domains 1-like) (SPECC1-like protein)		
Q13501	SQSTM_HUMAN	SQSTM1 ORCA OSIL	Sequestosome-1 (EBI3-associated protein of 60 kDa) (EBIAP) (p60) (Phosphotyrosine-independent ligand for the Lck SH2 domain of 62 kDa) (Ubiquitin-binding protein p62) (p62)	●	
P30626	SORCN_HUMAN	SRI	Sorcin (22 kDa protein) (CP-22) (CP22) (V19)	●	
P37108	SRP14_HUMAN	SRP14	Signal recognition particle 14 kDa protein (SRP14) (18 kDa Alu RNA-binding protein)	●	
P49458	SRP09_HUMAN	SRP9	Signal recognition particle 9 kDa protein (SRP9)		
Q9BXP5	SRRT_HUMAN	SRRT ARS2 ASR2	Serrate RNA effector molecule homolog (Arsenite-resistance protein 2)		
Q07955	SRSF1_HUMAN	SRSF1 ASF SF2 SF2P33 SFRS1 OK/SW-cl.3	Serine/arginine-rich splicing factor 1 (Alternative-splicing factor 1) (ASF-1) (Splicing factor, arginine/serine-rich 1) (pre-mRNA-splicing factor SF2, P33 subunit)		
Q08170	SRSF4_HUMAN	SRSF4 SFRS4 SRP75	Serine/arginine-rich splicing factor 4 (Pre-mRNA-splicing factor SRP75) (SRP001LB) (Splicing factor, arginine/serine-rich 4)	●	
P42224	STAT1_HUMAN	STAT1	Signal transducer and activator of transcription 1-alpha/beta (Transcription factor ISGF-3 components p91/p84)	●	●

O95793	STAU1_HUMAN	STAU1 STAU	Double-stranded RNA-binding protein Staufen homolog 1	●	●
Q9NUL3	STAU2_HUMAN	STAU2	Double-stranded RNA-binding protein Staufen homolog 2		
P31948	STIP1_HUMAN	STIP1	Stress-induced-phosphoprotein 1 (STI1) (Hsc70/Hsp90-organizing protein) (Hop) (Renal carcinoma antigen NY-REN-11) (Transformation-sensitive protein IEF SSP 3521)	●	
Q9Y3F4	STRAP_HUMAN	STRAP MAWD UNRIP	Serine-threonine kinase receptor-associated protein (MAP activator with WD repeats) (UNR-interacting protein) (WD-40 repeat protein PT-WD)		
Q8IX01	SUGP2_HUMAN	SUGP2 KIAA0365 SFRS14	SURP and G-patch domain-containing protein 2 (Arginine/serine-rich-splicing factor 14) (Splicing factor, arginine/serine-rich 14)		
O94901	SUN1_HUMAN	SUN1 KIAA0810 UNC84A	SUN domain-containing protein 1 (Protein unc-84 homolog A) (Sad1/unc-84 protein-like 1)		
Q8IZU3	SYCP3_HUMAN	SYCP3 SCP3	Synaptonemal complex protein 3 (SCP-3)		
P43405	KSYK_HUMAN	SYK	Tyrosine-protein kinase SYK (EC 2.7.10.2) (Spleen tyrosine kinase) (p72-Syk)	●	
Q8NF91	SYNE1_HUMAN	SYNE1 C6orf98 KIAA0796 KIAA1262 KIAA1756 MYNE1	Nesprin-1 (Enaptin) (KASH domain-containing protein 1) (KASH1) (Myocyte nuclear envelope protein 1) (Myne-1) (Nuclear envelope spectrin repeat protein 1) (Synaptic nuclear envelope protein 1) (Syne-1)		
Q92804	RBP56_HUMAN	TAF15 RBP56 TAF2N	TATA-binding protein-associated factor 2N (68 kDa TATA-binding protein-associated factor) (TAF(II)68) (TAFII68) (RNA-binding protein 56)		
P23193	TCEA1_HUMAN	TCEA1 GTF2S TFIIS	Transcription elongation factor A protein 1 (Transcription elongation factor S-II protein 1) (Transcription elongation factor TFIIS.o)		
P17987	TCPA_HUMAN	TCP1 CCT1 CCTA	T-complex protein 1 subunit alpha (TCP-1-alpha) (CCT-alpha) (Chaperonin containing T-complex polypeptide 1 subunit 1)		
Q13148	TADBP_HUMAN	TARDBP TDP43	TAR DNA-binding protein 43 (TDP-43)	●	
Q9H7E2	TDRD3_HUMAN	TDRD3	Tudor domain-containing protein 3		
O14746	TERT_HUMAN	TERT EST2 TCS1 TRT	Telomerase reverse transcriptase (EC 2.7.7.49) (HEST2) (Telomerase catalytic subunit) (Telomerase-associated protein 2) (TP2)		

P31483	TIA1_HUMAN	TIA1	Cytotoxic granule associated RNA binding protein TIA1 (Nucleolysin TIA-1 isoform p40) (RNA-binding protein TIA-1) (T-cell-restricted intracellular antigen-1) (TIA-1) (p40-TIA-1)	●	
Q01085	TIAR_HUMAN	TIAL1	Nucleolysin TIAR (TIA-1-related protein)		
Q9NYL9	TMOD3_HUMAN	TMOD3	Tropomodulin-3 (Ubiquitous tropomodulin) (U-Tmod)	●	
O95271	TNKS1_HUMAN	TNKS PARP5A PARPL TIN1 TINF1 TNKS1	Poly [ADP-ribose] polymerase tankyrase-1 (EC 2.4.2.30) (ADP-ribosyltransferase diphtheria toxin-like 5) (ARTD5) (Poly [ADP-ribose] polymerase 5A) (Protein poly-ADP-ribosyltransferase tankyrase-1) (EC 2.4.2.-) (TNKS-1) (TRF1-interacting ankyrin-related ADP-ribose polymerase) (Tankyrase I) (Tankyrase-1) (TANK1)		
Q9C0C2	TB182_HUMAN	TNKS1BP1 KIAA1741 TAB182	182 kDa tankyrase-1-binding protein		
Q92973	TNPO1_HUMAN	TNPO1 KPNB2 MIP1 TRN	Transportin-1 (Importin beta-2) (Karyopherin beta-2) (M9 region interaction protein) (MIP)		
O14787	TNPO2_HUMAN	TNPO2	Transportin-2 (Karyopherin beta-2b)	●	
Q9UPQ9	TNR6B_HUMAN	TNRC6B KIAA1093	Trinucleotide repeat-containing gene 6B protein	●	
Q15785	TOM34_HUMAN	TOMM34 URCC3	Mitochondrial import receptor subunit TOM34 (hTom34) (Translocase of outer membrane 34 kDa subunit)		
P09493	TPM1_HUMAN	TPM1 C15orf13 TMSA	Tropomyosin alpha-1 chain (Alpha-tropomyosin) (Tropomyosin-1)	●	
P07951	TPM2_HUMAN	TPM2 TMSB	Tropomyosin beta chain (Beta-tropomyosin) (Tropomyosin-2)		
P13693	TCTP_HUMAN	TPT1	Translationally-controlled tumor protein (TCTP) (Fortilin) (Histamine-releasing factor) (HRF) (p23)	●	
Q12933	TRAF2_HUMAN	TRAF2 TRAP3	TNF receptor-associated factor 2 (EC 2.3.2.27) (E3 ubiquitin-protein ligase TRAF2) (RING-type E3 ubiquitin transferase TRAF2) (Tumor necrosis factor type 2 receptor-associated protein 3)	●	
O14717	TRDMT_HUMAN	TRDMT1 DNMT2	tRNA (cytosine(38)-C(5))-methyltransferase (EC 2.1.1.204) (DNA (cytosine-5)-methyltransferase-like protein 2) (Dnmt2) (DNA methyltransferase homolog HsaIIP) (DNA MTase homolog HsaIIP) (M.HsaIIP) (PuMet)		

P19474	RO52_HUMAN	TRIM21 RNF81 RO52 SSA1	E3 ubiquitin-protein ligase TRIM21 (EC 2.3.2.27) (52 kDa Ro protein) (52 kDa ribonucleoprotein autoantigen Ro/SS-A) (RING finger protein 81) (Ro(SS-A)) (Sjoegren syndrome type A antigen) (SS-A) (Tripartite motif-containing protein 21)	●	●
Q14258	TRI25_HUMAN	TRIM25 EFP RNF147 ZNF147	E3 ubiquitin/ISG15 ligase TRIM25 (EC 6.3.2.n3) (Estrogen-responsive finger protein) (RING finger protein 147) (RING-type E3 ubiquitin transferase) (EC 2.3.2.27) (RING-type E3 ubiquitin transferase TRIM25) (Tripartite motif-containing protein 25) (Ubiquitin/ISG15-conjugating enzyme TRIM25) (Zinc finger protein 147)		●
Q9BRZ2	TRI56_HUMAN	TRIM56 RNF109	E3 ubiquitin-protein ligase TRIM56 (EC 2.3.2.27) (RING finger protein 109) (Tripartite motif-containing protein 56)		●
Q15654	TRIP6_HUMAN	TRIP6 OIP1	Thyroid receptor-interacting protein 6 (TR-interacting protein 6) (TRIP-6) (Opa-interacting protein 1) (OIP-1) (Zyxin-related protein 1) (ZRP-1)		
Q92574	TSC1_HUMAN	TSC1 KIAA0243 TSC	Hamartin (Tuberous sclerosis 1 protein)		
P26651	TTP_HUMAN	ZFP36 G0S24 NUP475 RNF162A TIS11A TTP	mRNA decay activator protein ZFP36 (G0/G1 switch regulatory protein 24) (Growth factor-inducible nuclear protein NUP475) (Tristetraprolin) (Zinc finger protein 36) (Zfp-36)		
Q9BQE3	TBA1C_HUMAN	TUBA1C TUBA6	Tubulin alpha-1C chain (EC 3.6.5.-) (Alpha-tubulin 6) (Tubulin alpha-6 chain) [Cleaved into: Detyrosinated tubulin alpha-1C chain]		
P0DPH7	TBA3C_HUMAN	TUBA3C TUBA2	Tubulin alpha-3C chain (EC 3.6.5.-) (Alpha-tubulin 2) (Alpha-tubulin 3C) (Tubulin alpha-2 chain) [Cleaved into: Detyrosinated tubulin alpha-3C chain]		
P68366	TBA4A_HUMAN	TUBA4A TUBA1	Tubulin alpha-4A chain (EC 3.6.5.-) (Alpha-tubulin 1) (Testis-specific alpha-tubulin) (Tubulin H2-alpha) (Tubulin alpha-1 chain)	●	
Q13509	TBB3_HUMAN	TUBB3 TUBB4	Tubulin beta-3 chain (Tubulin beta-4 chain) (Tubulin beta-III)	●	
Q3ZCM7	TBB8_HUMAN	TUBB8	Tubulin beta-8 chain (Tubulin beta 8 class VIII)		
P49411	EFTU_HUMAN	TUFM	Elongation factor Tu, mitochondrial (EF-Tu) (P43)	●	
P10599	THIO_HUMAN	TXN TRDX TRX TRX1	Thioredoxin (Trx) (ATL-derived factor) (ADF) (Surface-associated sulphydryl protein) (SASP) (allergen Hom s Trx)		

Q01081	U2AF1_HUMAN	U2AF1 U2AF35 U2AFBP FP793	Splicing factor U2AF 35 kDa subunit (U2 auxiliary factor 35 kDa subunit) (U2 small nuclear RNA auxiliary factor 1) (U2 snRNP auxiliary factor small subunit)		
P22314	UBA1_HUMAN	UBA1 A1S9T UBE1	Ubiquitin-like modifier-activating enzyme 1 (EC 6.2.1.45) (Protein A1S9) (Ubiquitin-activating enzyme E1)	●	
Q5T6F2	UBAP2_HUMAN	UBAP2 KIAA1491	Ubiquitin-associated protein 2 (UBAP-2) (RNA polymerase II degradation factor UBAP2)		
Q14157	UBP2L_HUMAN	UBAP2L KIAA0144 NICE4	Ubiquitin-associated protein 2-like (Protein NICE-4) (RNA polymerase II degradation factor UBAP2L)		
P11441	UBL4A_HUMAN	UBL4A DXS254E GDX UBL4	Ubiquitin-like protein 4A (Ubiquitin-like protein GDX)		
Q92900	RENT1_HUMAN	UPF1 KIAA0221 RENT1	Regulator of nonsense transcripts 1 (EC 3.6.4.12) (EC 3.6.4.13) (ATP-dependent helicase RENT1) (Nonsense mRNA reducing factor 1) (NORF1) (Up-frameshift suppressor 1 homolog) (hUpf1)		
Q14694	UBP10_HUMAN	USP10 KIAA0190	Ubiquitin carboxyl-terminal hydrolase 10 (EC 3.4.19.12) (Deubiquitinating enzyme 10) (Ubiquitin thioesterase 10) (Ubiquitin-specific-processing protease 10)	●	
P45974	UBP5_HUMAN	USP5 ISOT	Ubiquitin carboxyl-terminal hydrolase 5 (EC 3.4.19.12) (Deubiquitinating enzyme 5) (Isopeptidase T) (Ubiquitin thioesterase 5) (Ubiquitin-specific-processing protease 5)	●	
P50552	VASP_HUMAN	VASP	Vasodilator-stimulated phosphoprotein (VASP)		
P55072	TERA_HUMAN	VCP HEL-220 HEL-S-70	Transitional endoplasmic reticulum ATPase (TER ATPase) (EC 3.6.4.6) (15S Mg(2+)-ATPase p97 subunit) (Valosin-containing protein) (VCP)		●
O43379	WDR62_HUMAN	WDR62 C19orf14	WD repeat-containing protein 62		
Q8IZH2	XRN1_HUMAN	XRN1 SEP1	5'-3' exoribonuclease 1 (EC 3.1.13.-) (Strand-exchange protein 1 homolog)		
P54577	SYYC_HUMAN	YARS1 YARS	Tyrosine--tRNA ligase, cytoplasmic (EC 6.1.1.1) (Tyrosyl-tRNA synthetase) (TyrRS) [Cleaved into: Tyrosine--tRNA ligase, cytoplasmic, N-terminally processed]		
P67809	YBOX1_HUMAN	YBX1 NSEP1 YB1	Y-box-binding protein 1 (YB-1) (CCAAT-binding transcription factor I subunit A) (CBF-A) (DNA-	●	

			binding protein B) (DBPB) (Enhancer factor I subunit A) (EFI-A) (Nuclease-sensitive element-binding protein 1) (Y-box transcription factor)		
P16989	YBOX3_HUMAN	YBX3 CSDA DBPA	Y-box-binding protein 3 (Cold shock domain-containing protein A) (DNA-binding protein A) (Single-strand DNA-binding protein NF-GMB)		
P07947	YES_HUMAN	YES1 YES	Tyrosine-protein kinase Yes (EC 2.7.10.2) (Proto-oncogene c-Yes) (p61-Yes)		
Q9BYJ9	YTHD1_HUMAN	YTHDF1 C20orf21	YTH domain-containing family protein 1 (DF1) (Dermatomyositis associated with cancer putative autoantigen 1) (DACA-1)	●	
Q9Y5A9	YTHD2_HUMAN	YTHDF2 HGRG8	YTH domain-containing family protein 2 (DF2) (CLL-associated antigen KW-14) (High-glucose-regulated protein 8) (Renal carcinoma antigen NY-REN-2)		
Q7Z739	YTHD3_HUMAN	YTHDF3	YTH domain-containing family protein 3 (DF3)		
P31946	1433B_HUMAN	YWHAB	14-3-3 protein beta/alpha (Protein 1054) (Protein kinase C inhibitor protein 1) (KCIP-1) [Cleaved into: 14-3-3 protein beta/alpha, N-terminally processed]	●	
Q04917	1433F_HUMAN	YWHAH YWHA1	14-3-3 protein eta (Protein AS1)	●	
P27348	1433T_HUMAN	YWHAQ	14-3-3 protein theta (14-3-3 protein T-cell) (14-3-3 protein tau) (Protein HS1)	●	
Q9H171	ZBP1_HUMAN	ZBP1 C20orf183 DLM1	Z-DNA-binding protein 1 (DNA-dependent activator of IFN-regulatory factors) (DAI) (Tumor stroma and activated macrophage protein DLM-1)		●
Q5D1E8	ZC12A_HUMAN	ZC3H12A MCPIP MCPIP1	Endoribonuclease ZC3H12A (EC 3.1.-.-) (Monocyte chemotactic protein-induced protein 1) (MCP-induced protein 1) (MCPIP-1) (Regnase-1) (Reg1) (Zinc finger CCCH domain-containing protein 12A)		●
Q6PJT7	ZC3HE_HUMAN	ZC3H14	Zinc finger CCCH domain-containing protein 14 (Mammalian suppressor of tau pathology-2) (MSUT-2) (Renal carcinoma antigen NY-REN-37)		
Q8IWR0	Z3H7A_HUMAN	ZC3H7A ZC3H7 ZC3HDC7 HSPC055	Zinc finger CCCH domain-containing protein 7A		
Q9UGR2	Z3H7B_HUMAN	ZC3H7B KIAA1031	Zinc finger CCCH domain-containing protein 7B (Rotavirus 'X'-associated non-structural protein) (RoXaN)		

Q7Z2W4	ZCCHV_HUMAN	ZC3HAV1 ZC3HDC2 PRO1677	Zinc finger CCCH-type antiviral protein 1 (ADP-ribosyltransferase diphtheria toxin-like 13) (ARTD13) (Inactive Poly [ADP-ribose] polymerase 13) (PARP13) (Zinc finger CCCH domain-containing protein 2) (Zinc finger antiviral protein) (ZAP)		●
Q9HA38	ZMAT3_HUMAN	ZMAT3 PAG608 WIG1	Zinc finger matrin-type protein 3 (Zinc finger protein WIG-1) (p53-activated gene 608 protein)		
Q14966	ZN638_HUMAN	ZNF638 NP220 ZFML	Zinc finger protein 638 (Cutaneous T-cell lymphoma-associated antigen se33-1) (CTCL-associated antigen se33-1) (Nuclear protein 220) (Zinc finger matrin-like protein)		

Table S2: Viral pathways associated with the stress granule proteome

Biological Process (Gene Ontology)	Count in network	Signal	False discovery rate
**Stress granule assembly**	11/24	2.9	3.01e-12
Response to virus	32/356	2.57	1.78e-18
Response to virus	8/15	2.23	3.32e-09
Defense response to virus	23/252	2.12	3.32e-13
Viral process	20/242	1.78	9.77e-11
Regulation of viral life cycle	15/139	1.76	2.25e-09
Regulation of viral process	16/161	1.74	1.50e-09
IRES-dependent viral translational initiation	6/11	1.64	7.80e-07
Viral translational termination-reinitiation	5/5	1.58	1.77e-06
Viral gene expression	9/52	1.51	5.44e-07
Positive regulation of viral process	9/62	1.36	1.95e-06
Regulation of viral genome replication	10/86	1.3	2.11e-06
Positive regulation of defense response to virus by host	7/34	1.28	8.40e-06
Cellular response to virus	9/87	1.08	2.29e-05
Regulation of defense response to virus	8/76	0.97	8.45e-05
Cytoplasmic pattern recognition receptor signaling pathway in response to virus	4/11	0.87	0.00060
Viral life cycle	11/178	0.85	0.00011
Negative regulation of viral process	8/94	0.82	0.00033
Viral penetration into host nucleus	3/4	0.78	0.0016
Positive regulation of viral genome replication	5/32	0.75	0.0013
Positive regulation of viral life cycle	4/22	0.62	0.0045

Negative regulation of viral genome replication	5/56	0.5	0.0108
Entry of viral genome into host nucleus through nuclear pore complex via importin	2/2	0.47	0.0204
Viral genome replication	3/20	0.37	0.0424

Table S3: Categorization of Mammalian Stress Granule Proteins

Mammalian Stress Granule Proteins	n=458
Virus-associated Proteins	n=50
Neuronal-associated Proteins	n=396
tau Interactors	n=138
Common Viral and Neuronal Proteins	n=47
Common Viral Proteins and tau Interactors	n=16
Common Viral, Neuronal Proteins and tau Interactors	n=15

Methodology

Data Collection

Using a computational biology approach, a comprehensive list of proteins identified in mammalian stress granules was compiled, using the Mammalian Stress Granules Proteome (MSGP) database (https://msgp.pt/). Data was collected from database version 12.0 on March/20/2025. The full listing of proteins is presented in **Table S1**. Categorization of stress granule proteins based on their expression in neurons, predicted interactions with tau and evidence of involvement in viral pathways is listed in **Table S2**.

STRING network analysis

The 458 stress granule proteins identified in the MSGP database were input into STRING (https://string-db.org/, version 12.0), to predict a protein-protein interaction network. Network settings were set to *Homo sapiens* for organism, with a full network was presented based on database sources "textmining", "experiments" and "databases", and a minimum required interaction score of 0.900 (highest confidence) and the addition of a maximum 10 first shell interactors. The network edges represent confidence in data support. Network statistics were: number of nodes (182), number of edges (411), average node degree (4.52), average local clustering coefficient (0.489), expected number of edges (87) and a PPI enrichment p-value of < 1.0e-16.

Through evaluation of the Analysis section of the STRING output, a table of functional enrichments within the network was presented (**Figure 1**). A more detailed analysis of the Biological Process – Gene Ontology (GO) was conducted by assessing the GO terms for specific search terms. A table was created by identifying GO-Terms containing the term 'Virus' or 'Viral' (**Table S3**) and their associated proteins (**Tables S1, S2**). This same methodology was applied to the STRING output section of Cellular Components – Gene Ontology (GO). There were 132 cellular components presented in the STRING output, and 32 were identified as being neuronal. The list of associated proteins for those 32 cellular components was compiled with duplicates removed to create the master list of stress granule proteins associated with neuronal cellular components (n=396). The BioGRID database (MAPT Result Summary | BioGRID; version 3.5, accessed June/18/2025) was used to compile a list of proteins known to interact with tau (n=1,112).

The previously compiled list of the n=458 stress granule proteins (**Table S1**) was then cross-referenced against the 1,112 known tau interactors, identifying 138 Tau-associated proteins among the stress granule proteome. Ultimately, this categorization identified a total of 15 proteins which share the commonality of being viral associated, located neuronally, and are also known tau interactors (**Table S1**, purple dots).

The final compiled list of proteins from **Table S1** was presented in a STRING network, with a payload depicting the categorized proteins based on their respective associations. Tau-associated nodes were coloured blue (Hex#64b5f6). Nodes for viral-associated proteins were coloured light red (#64b5f6). The nodes representing both viral and tau association were coloured with the additive colour purple (Hex #6a1b9a). Any stress granule proteins not related to the previous categories were coloured gray (Hex #e0e0e0). Clusters of nodes were grouped based on related cellular pathways as identified by GO labels.

NCCIT Cell Culture

The NCCIT cells were purchased from American Type Culture Collection. The NCCIT cell line (ATTC CRL-2073) is derived from human Pluripotent Embryonal Carcinoma, and was maintained in RPMI 1640 medium supplemented with 10% heat-inactivated fetal bovine serum (Hyclone, SH30396.03), in a humidified chamber containing 5% $CO2$ at 37°C. NCCIT cells were seeded into an 18-well microscope chamber slides (Cat: 81816, IBIDI) at a density of 5 $x10^4$ cells/mL, and grown untreated for 24 h.

Immunofluorescence

Cells seeded on chamber slides were washed in cold PBS (Cat: SH3025602, Cytiva HyClone™) and fixed with 4% paraformaldehyde (Cat: 15710, Electron Microscopy Science,) for 5 min and rinsed with 1× PBS. Cells were permeabilised with 100 µL of TBS-T (TBS with 0.05% TritonX-100) and blocked with 100 µL of TBS-T containing 5% donkey serum (Cat: D9663, Sigma-Aldrich) for 30 min. Cells were incubated in primary antibodies for two hours at 37ºC, followed by incubation in appropriate fluorophore-conjugated secondary antibodies (1:250 dilution) for one hour in dark on a rocker. Rabbit anti-ERVK IN-2 (1:200; custom antibody), mouse anti-tau AD [GT-38] Conformation-Specific monoclonal (1:500; Abcam, ab246808) and chicken anti-tau polyclonal (1:50; Abcam, ab75714) were used as primary antibodies. F(ab)2- goat

anti-rabbit IgG (H+L) cross-adsorbed secondary antibody Alexa Fluor 488 (A-11070), F(ab)2-goat anti-mouse IgG (H+L) cross-adsorbed secondary antibody Alexa Fluor 594 (A-11020) and Goat anti-Chicken IgY (H+L) secondary antibody, Alexa Fluor™ 647 (A21449) were from Thermofisher Scientific. Nuclei were counter-stained with DAPI (1:50,000 dilution; Molecular Probes, D1306). Controls were prepared by immunostaining without the primary antibodies. Cells were kept in 1x PBS until image capture.

Confocal microscopy

Confocal images were captured using a Fluoview FV1200 laser scanning confocal microscope (Olympus, Japan). The acquisition was controlled by FV10-ASW4.0 software. CellSens Dimension Microscope Imaging 4.2 software was utilized to process, detect, measure images, and create orthogonal views of viral inclusions.
